# An amber obligate active site-directed ligand evolution technique for phage display

**DOI:** 10.1038/s41467-020-15057-7

**Published:** 2020-03-13

**Authors:** Jeffery M. Tharp, J. Trae Hampton, Catrina A. Reed, Andreas Ehnbom, Peng-Hsun Chase Chen, Jared S. Morse, Yadagirri Kurra, Lisa M. Pérez, Shiqing Xu, Wenshe Ray Liu

**Affiliations:** 1 The Texas A&M Drug Discovery Laboratory, Department of Chemistry, Texas A&M University, College Station, TX 77843 USA; 2 Laboratory for Molecular Simulation, Department of Chemistry, Texas A&M University, College Station, TX 77843 USA; 3 Department of Biochemistry & Biophysics, Texas A&M University, College Station, TX 77843 USA

**Keywords:** Peptides, Screening, Biologics, Bacteriophages

## Abstract

Although noncanonical amino acids (ncAAs) were first incorporated into phage libraries through amber suppression nearly two decades ago, their application for use in drug discovery has been limited due to inherent library bias towards sense-containing phages. Here, we report a technique based on superinfection immunity of phages to enrich amber-containing clones, thus avoiding the observed bias that has hindered incorporation of ncAAs into phage libraries. We then take advantage of this technique for development of active site-directed ligand evolution of peptides, where the ncAA serves as an anchor to direct the binding of its peptides to the target’s active site. To demonstrate this, phage-displayed peptide libraries are developed that contain a genetically encoded butyryl lysine and are subsequently used to select for ligands that bind SIRT2. These ligands are then modified to develop low nanomolar inhibitors of SIRT2.

## Abstract (web summary)

Most epigenetic regulator inhibitors target tunnels of active sites, rather than the peptide binding groove, leading to concerns with low selectivity. Here the authors use an amber obligate phage library to rapidly identify isoform-selective inhibitors of SIRT2.

## Introduction

Incentivized by funding agencies and the pharmaceutical industry, the past two decades have witnessed a revolution in the drug discovery field. Many compound libraries and their high-throughput screening techniques have been developed successfully^1,2^. For an established biomacromolecule target, the identification of lead compounds that can serve as small-molecule ligands has become relatively straightforward. A bottleneck toward pushing a drug discovery effort to the clinic is the structure–activity relationship (SAR) study that provides essential information for optimizing the structure of a lead molecule to achieve improved potency and biological activity^3,4^. An SAR study involves varying functional groups in the original structure of a lead molecule, adding additional chemical moieties, or both. It could involve huge efforts of chemical synthesis and activity analysis, but still leads to attrition. Inspired by the phage display technique that allows for easy construction of a large peptide library (>10^9^) and creates a physical link between displayed peptides and their encoding DNA for efficient enrichment and identification of peptides that are bound to a particular target, we sought to combine a part of the SAR concept with the phage display technique to develop an amber-obligate phage-assisted, active site-directed ligand evolution technique for the rapid identification of potent ligands for epigenetic regulators. Here, we wish to report our progress on this exciting research front.

**Fig. 1:**
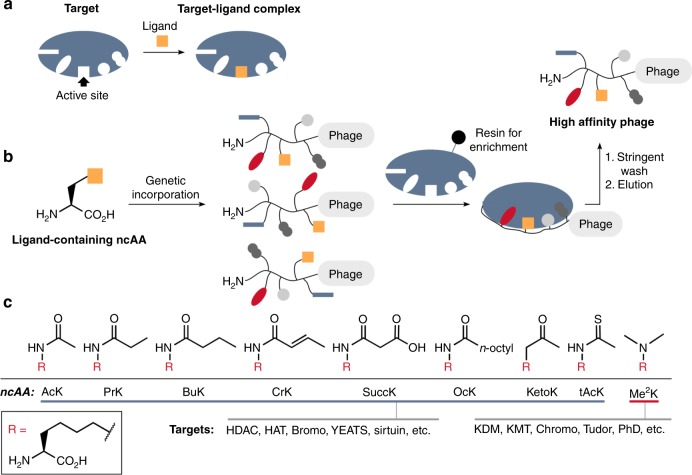
Overview of phage-assisted active site-directed ligand evolution. **a** A diagram that illustrates the interaction between a protein target and a ligand. **b** The genetic incorporation of a ligand-fused ncAA into phage-displayed peptides for active site-directed binding to a protein target that is followed by stringent wash and elution to select high-affinity and selective phages. **c** ncAAs that have been genetically incorporated and can potentially serve as ligands for epigenetic regulators (HAT histone acetyltransferase, KDM protein lysine demethylase, KMT protein lysine methyltransferase, Bromo, YEATS, chromo, Tudor, and PhD are epigenetic reader domains).

In eukaryotes, chromatin undergoes post-translational modifications to its scaffold histone proteins. These epigenetic modifications serve critical functions in cell development, proliferation, and differentiation. Aberrant modifications lead to diverse disorders that include many cancers^5,6^. As such, epigenetic regulators, such as writers, erasers, and readers, are important drug targets for disease intervention^7,8^. Successful epigenetics-regulating drugs include a number of histone deacetylase (HDAC) inhibitors that have been approved to treat hematopoietic cancers^9–11^. Most epigenetic regulators entail a binary mode to recognize their substrates. In this recognition mode, the active site of an epigenetic regulator binds selectively to the post-translationally modified or to-be-modified amino acid residue in a protein substrate, and an annexing peptide-binding groove provides selectivity, to some degree, toward the protein substrate’s amino acid sequence^12^. Many isofunctional epigenetic regulators share structural similarity in their active sites, but display structural variation in their peptide-binding grooves. A compound that interacts strongly with the peptide-binding groove of an epigenetic regulator is likely selective toward it over other isofunctional proteins. However, most inhibitors developed for epigenetic regulators primarily target their active sites, leading to significant concerns of low selectivity. In order to identify selective inhibitors for epigenetic regulators in a high-throughput fashion, we now propose a phage-assisted, active site-directed ligand evolution approach as shown in Fig. 1. A ligand that specifically binds to the active site of an epigenetic regulator can be integrated into a noncanonical amino acid. This ligand-fused ncAA is then genetically incorporated into a phage-displayed, sequence-randomized peptide library. In the phage-displayed peptides, the fused ligand is expected to serve as an anchor to bind to the active site of the epigenetic regulator, and amino acid residues flanking the ligand-fused ncAA provide additional interactions with the peptide-binding groove of the epigenetic regulator to improve both affinity and selectivity. This concept is similar to a general practice in SAR studies that add additional chemical moieties for improved affinity and selectivity. Doing selection against the epigenetic regulator under stringent washing and elution will quickly enrich high-affinity phages whose displayed peptides can be easily determined by sequencing their coding DNAs. In order to incorporate a ligand-fused ncAA into a phage-displayed peptide library, we adopt the well-established amber suppression-based ncAA mutagenesis method^13,14^. So far, a number of post-translationally modified lysines and their analogs have been genetically incorporated using the amber suppression method (Fig. 1c)^15–23^. These ncAAs can serve as binding anchors for a number of epigenetic regulators to quickly evolve their potent and selective ligands using our proposed technique.

In the following sections, we describe the generation of an amber-obligate phage library to display an *N* ^Ɛ^-butyryl-l-lysine (BuK) for rapid identification of selective ligands for SIRT2, an NAD-dependent deacetylase. Substitution of thiobutyryl (tBuK) and thiomyristoyl (tMyK) analogs into the selected peptides results in inhibitors of SIRT2 that are selective against other sirtuin isoforms and much more potent than previously reported small-molecule inhibitors, TB and TM. Given these results and current abilities to genetically encode post-translational modifications, this technique is useful in identifying inhibitors of various epigenetic readers, writers, and erasers.

## Results

### Construction of an amber-obligate phage library

**Fig. 2:**
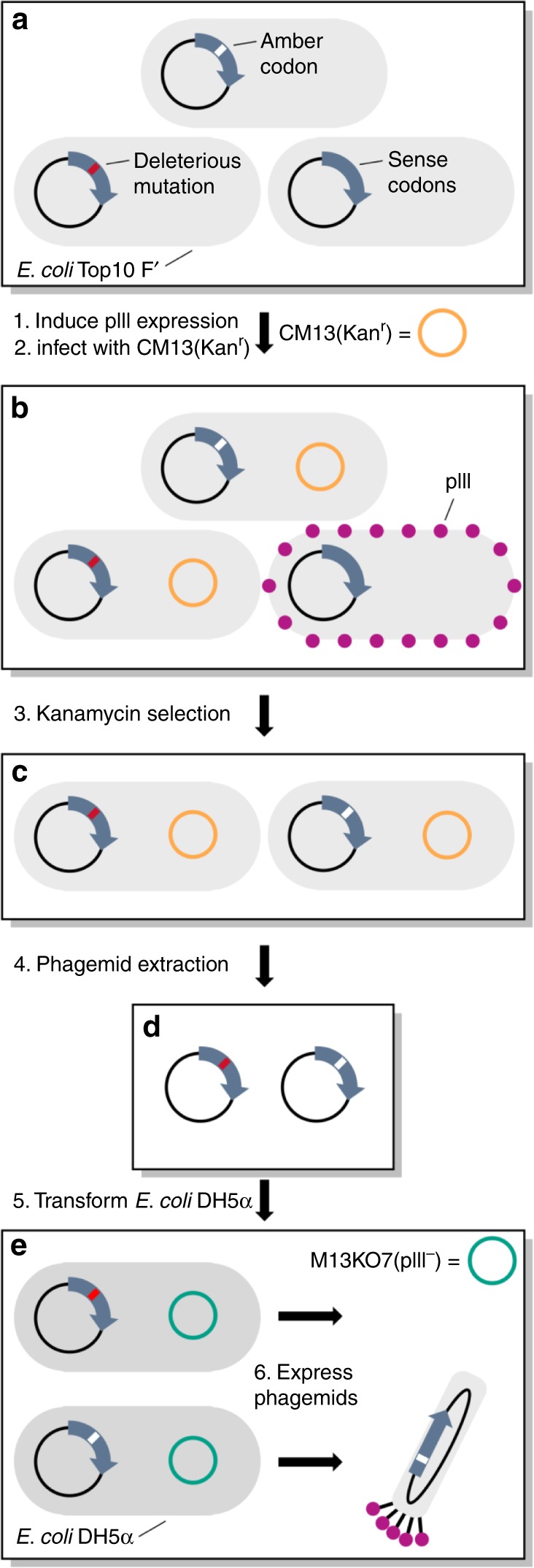
A schematic representation of a method for constructing an amber-obligate phage display library by superinfection-immunity-based selection. **a**, **b** Following cloning, the naive phagemid library is used to transform non-amber-suppressing *E. coli* bearing an F sex pilus. The expression of pIII is induced with IPTG, and shortly after, cells are superinfected with the CM13 helper phage (**a**). The expression of pIII in cells harboring a copy of the library that contains only sense codons renders these cells immune to superinfection (**b**). **c** Changing the media to one containing kanamycin allows for the selective growth of cells harboring a copy of the library that contains in-frame amber codons. Cells harboring a copy of the library with deleterious mutations also pass this selection. **d**, **e** The phagemid library is purified (**d**) and used to transform DH5α, an amber-suppressing strain of *E. coli* (**e**). Complementation of the phagemid with a pIII-knockout helper phage in *E. coli* DH5α allows for the production of phagemid particles only from cells harboring a copy of the peptide-pIII library containing in-frame amber codons.

In order to carry out our proposed phage-assisted, active site-directed ligand evolution, it is necessary that all clones in a phage display library express peptides that contain a ligand-fused ncAA. The use of this library will avoid, in principle, the enrichment of clones that do not bind to the active site of an epigenetic regulator. Therefore, the construction of an amber-obligate phage library in which every clone has at least one in-frame amber codon for coding the ligand-fused ncAA is essential. Previous methods for the construction of amber-obligate phage libraries utilized primer pairs in which the position of the amber codon is fixed; therefore, its coded ncAA was fixed, and all other positions were randomized^24^. We adopted this approach initially to construct a heptapeptide library based on a phagemid pADL-10b in which the amber codon was fixed at the seventh coding position and all other six were randomized. We noted that this library contained undesired clones that were either the original phagemid or clones caused by synthetic errors in DNA primers (Supplementary Fig. 1a). After six rounds of propagation in *E. coli* DH5α, an amber-suppressing *supE* strain^25^, and with a helper phage for phage production, undesired clones were heavily enriched due to their significant growth advantage over amber-containing clones (Supplementary Fig. 1b). Therefore, we abandoned this approach, and subsequently invented a technique, as illustrated in Fig. 2, to construct an amber obligate and more diverse phage library in which the position of the amber codon was randomly distributed throughout the peptide-coding DNA sequence. This technique exploits a phenomenon of filamentous (Ff) phage biology known as superinfection immunity. Briefly, superinfection immunity describes the resistance of an infected bacterium to further infection by the same type of phage. For *E. coli* infected with Ff phages, superinfection immunity is granted by the phage coat protein pIII that is expressed in the host from the resident phage^26^. Endogenously expressed pIII saturates TolA, an *E. coli* cell surface protein used by Ff phages as a receptor for host adsorption, blocking adsorption and therefore superinfection by competing phages^27^. Our technique involved two steps. In the first step, we constructed a naive library as a fusion to the phage coat protein pIII gene. The library was constructed using degenerate primers with NNK triplet nucleotide sets that encrypted randomly one amber codon and 31 sense codons for 20 amino acids. In the second step, we exploited the principle of superinfection immunity to select for amber-containing clones, removing those that contain only sense codons or deleterious mutations from the library pool.

To clone the initial library, we introduced seven NNK codons via site-saturation mutagenesis as a genetic fusion to the N terminus of pIII in pADL-10b. Following cloning, we used the purified library to transform Top10F’, a non-amber-suppressing *E. coli* strain yielding 1.4 × 10^9^ transformants. Next, we carried out superinfection-immunity-based selection to enrich clones that contained in-frame amber codons. We grew transformed Top10F’ cells to the early log phase at which point we induced the expression of pIII (Fig. 2a). Under these conditions, pIII is expressed in cells that harbor a library clone with only sense codons; however, translation termination prevents the expression of pIII in cells harboring an amber-containing clone (Fig. 2b). Due to superinfection immunity granted by endogenous pIII, the former is immune to superinfection and the latter is vulnerable to superinfection. We then infected the culture with the CM13 helper phage that bears a gene conferring kanamycin resistance. Following superinfection with CM13, we changed the media to one containing kanamycin, which allowed for the selective growth of cells susceptible to superinfection, i.e., those harboring an amber-containing clone (Fig. 2c).

**Fig. 3:**
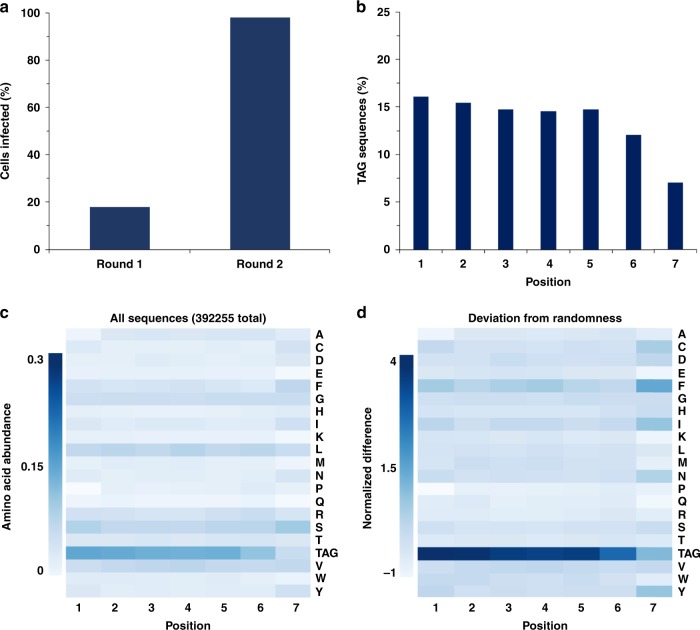
Characterization of the amber-obligate phage library generated from superinfection-immunity-based selection. **a** During two rounds of superinfection-immunity-based selection, the percentage of the population that was susceptible to superinfection by the CM13 helper phage (e.g., clones that contained amber codons) was monitored by growth on kanamycin. The increase in the number of cells that were susceptible to superinfection between rounds 1 and 2 is indicative of the successful selection of library clones containing amber codons. **b** The occurrence percentages of TAG codons across the seven randomized positions in the enriched amber- obligate phage library. **c** A heat map showing the occurrence abundance (amount at position/total sequences) of each amino acid or the TAG codon across all library positions. **d** A heat map showing deviation from theoretical randomization for each amino acid or the TAG codon at all library positions. Deviation is calculated by (observed abundance-expected abundance)/expected abundance.

We sequenced several clones after one round of superinfection-immunity-based selection. The sequences revealed that, along with amber-containing clones, a number of clones that contain deleterious mutations in the peptide or pIII-coding region were also enriched (Supplementary Fig. 2a). These mutations prevented the expression of functional pIII, allowing the clones to pass through the selection. To cull these unwanted clones, we purified the phagemid library for transforming amber-suppressing DH5α cells. Expressing the library in DH5α allowed pIII production in amber-containing clones, but not in those that contained deleterious mutations. Complementation with M13KO7(pIII^−^) (Supplementary Figs. 3, 4), a helper phage with a nonsense (TAA) mutation in gene III, and therefore unable to produce its own pIII, resulted in the selective production of phagemid particles from clones that contained amber codon(s) within the peptide-coding sequence (Fig. 2d, e). To confirm the successful removal of deleterious clones, we sequenced several of the resulting phagemids. None of them contained deleterious mutations that would prevent pIII expression (Supplementary Fig. 2b). Following passage through DH5α, we purified the phagemid particles and used them to infect Top10 F’ cells for an additional round of selection. Over two rounds of superinfection-immunity-based selection, we monitored amber codon enrichment by measuring the percentage of the population of phagemid-transformed *E. coli* Top10F’ cells that was susceptible to superinfection. In the first round, 18% of the population was susceptible to superinfection by the CM13 helper phage. This is consistent with our theoretical calculation that predicted 19.9% of the clones to contain at least one amber codon. After the second round of selection, greater than 98% of the population was susceptible to superinfection, suggesting a large increase in the number of clones that contained amber codon(s) between rounds 1 and 2 (Fig. 3a). Sequencing of the library after the second round of superinfection-immunity-based selection confirmed the successful selection as all of the sequenced clones (*n* = 10) contained an amber codon within the random sequence (Supplementary Fig. 5). To verify that the library diversity could sustain its propagation, we cultivated the amber-enriched phage library five times, and then subjected the library to deep sequencing. Several R scripts were written for data analysis (Supplementary R Scripts 1–3). Out of about 400,000 reads that were filtered for errors using paired-end processing, we confirmed that at least 93% of all clones still contained at least one in-frame amber codon. The analysis of all sequencing data showed some codon bias, but largely random distribution of non-amber codons and heavily enriched amber codons at most coding positions (Fig. 3b–d and Supplementary Figs. 6, 7), confirming the high quality of the amber-enriched phage library. The deep-sequencing data showed some enriched non-amber clones, indicating that additional superinfection-immunity-based selection might be necessary after rounds of library propagation.

### Genetic incorporation of ncAAs into phage libraries

**Fig. 4:**
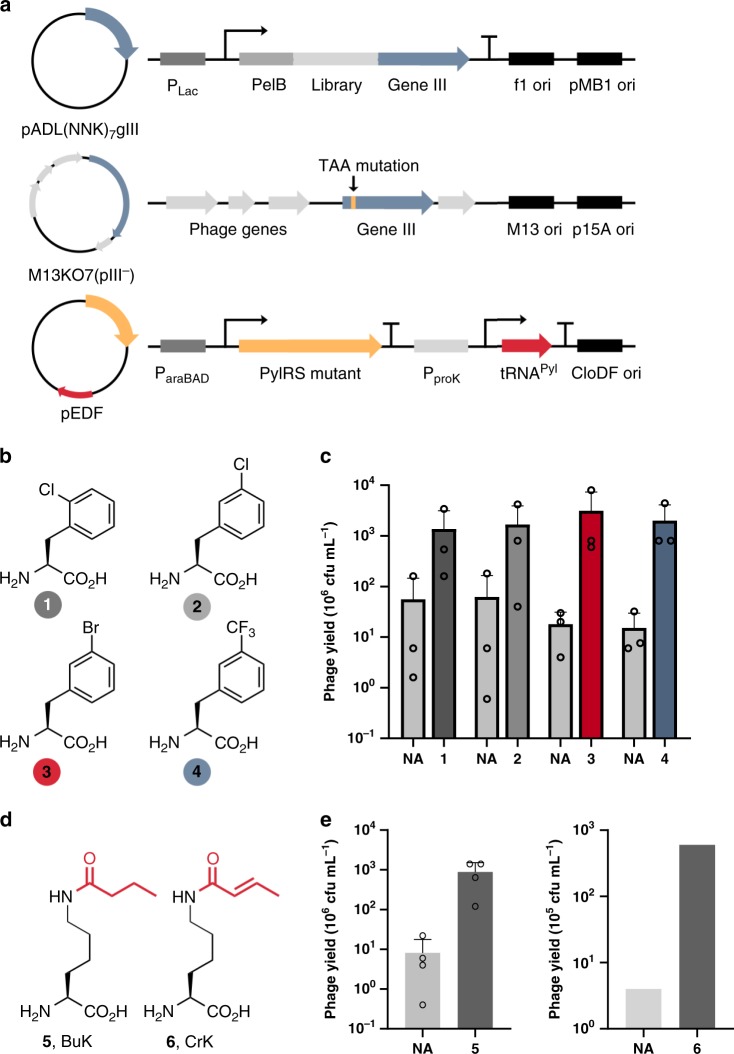
The genetic incorporation of ncAAs into a phage-displayed peptide library. **a** A schematic representation of the three-plasmid system used to incorporate ncAAs into the peptide library. **b** The structures of phenylalanine derivatives **1**–**4**. **c** Phage yield in the presence and absence (NA) of 4 mM ncAAs **1**–**4**. The yield is displayed in millions of colony-forming units per milliliter of culture media. Error bars represent one standard deviation of the mean of three independent experiments (*n* = 3). **d** The structures of two post-translationally modified lysines, **5** and **6**. **e** Phage yield in the presence and absence (NA) of **5** and **6**. The yield is displayed in millions or hundreds of thousands of colony-forming units for compounds **5** and **6**, respectively. Error bars represent one standard deviation of the mean of four independent experiments (*n* = 4). Data for the incorporation of ncAAs are available in the Source Data File.

After the construction of an amber-obligate phage library, we then tested its use to produce ncAA-containing phages. We first examined the incorporation of phenylalanine derivatives using PhdRS, a N346A/C348A mutant of pyrrolysyl-tRNA synthetase (PylRS)^28,29^. To produce phagemid particles, we transformed Top10 cells with three plasmids: (1) pADL(NNK)_7_gIII that encoded the amber-obligate phagemid library, (2) pEDF-PhdRS that contained genes encoding PhdRS and tRNA^Pyl^, and (3) M13KO7(pIII^−^) (Fig. 4a). Growing transformed cells in the presence of one of the ncAAs **1**–**4** (Fig. 4b) yielded a 36- to 83-fold increase in phagemid particle production compared with when no ncAA was present (Fig. 4c). The ncAA-dependent increase in particle yield is consistent with suppression of the amber codon by tRNA^Pyl^, and indicates the successful incorporation of the ncAAs into phage-displayed peptides. The low level of background phagemid production in the absence of an ncAA is likely due to phenylalanine incorporation, as we have shown that PhDRS mis-aminoacylates tRNA^Pyl^ with phenylalanine, albeit to a small degree^29^. We also tested the incorporation of two lysine derivatives, BuK (**5**) and *N*^Ɛ^-crotonyl-l-lysine (**6**, CrK) (Fig. 4d). Both ncAAs represent natural lysine post-translational modifications. Peptide libraries containing these ncAAs could be potentially used for active site-directed evolution of ligands for epigenetic regulators. To incorporate BuK and CrK, we used the previously reported BuKRS, a Y384W mutant of PylRS^19^. We expressed the phagemid library in Top10 cells containing pEDF-BuKRS and M13KO7(pIII^−^), which led to minimal particle production in the absence of an ncAA. Whereas, providing BuK or CrK resulted in an increase in the particle yield of nearly 100-fold, demonstrating the successful incorporation of these ncAAs into phage-displayed peptides (Fig. 4e).

Phage display-based directed evolution experiments demand robust libraries that can endure iterative rounds of amplification and target-directed selection. To investigate the integrity of our amber-obligate library in the face of iterative rounds of amplification and selection, we performed mock selections against both TEV protease (see Supplementary Fig. 8 for sodium dodecyl sulfate polyacrylamide gel electrophoresis (SDS-PAGE)) and streptavidin. We incubated the phage-displayed heptapeptide library (~5 × 10^10^ colony-forming units (cfu)) that had been expressed in the presence of **3** or **4** with biotin-conjugated TEV protease that was immobilized on streptavidin-coated beads, removed unbound phagemid particles by washing, and eluted with a pH 2 buffer. Following each selection round, we amplified the eluted phagemid particles in Top10 cells containing pEDF-PhdRS and M13KO7(pIII^−^) for further rounds of selection. We carried out selection against streptavidin by following similar procedures, except that only a phage library containing **3** was used, streptavidin-coated plates were used for selection, and the elution was done by competitive washing using 0.1 mM biotin. In total, we performed four rounds of amplification and selection. Phagemid clones were sequenced after the third and fourth rounds. Notably, even after four rounds of amplification and selection, all of the clones that were sequenced contained **3** or **4** within the peptide sequence, as indicated by the presence of an in-frame amber codon (Supplementary Figs. 9, 10). Both selections also showed significant convergence of finally selected clones.

### Phage-assisted identification of potent SIRT2 inhibitors

With a robust amber-obligate phage library available for the production of ncAA-containing phages, next we carried out the demonstration of our proposed phage-assisted, active site-directed ligand evolution concept using a model epigenetic regulator, SIRT2. SIRT2 is an NAD^+^-dependent lysine deacylase that catalytically removes post-translational lysine acylations from proteins^30^. Recently, SIRT2 inhibitors have been shown to have pronounced anticancer activity in cell and animal models of human cancer^31–33^. SIRT2 inhibitors also show promise in the treatment of age-related neurodegenerative disease^34,35^. Therefore, there is currently great interest in the development of potent and selective SIRT2 inhibitors. It has been found that SIRT2 catalytically removes lysine butyrylation. Therefore, we envisioned that a phage-displayed BuK-containing peptide library could be used for active site-directed ligand evolution to reveal peptide sequences that bind to the SIRT2 active site and its annexing peptide-binding groove. To this end, we used our amber-obligate phage library to produce a phage display library of greater than 300 million BuK-containing peptides, and selected for sequences within this library that bind SIRT2.

**Fig. 5:**
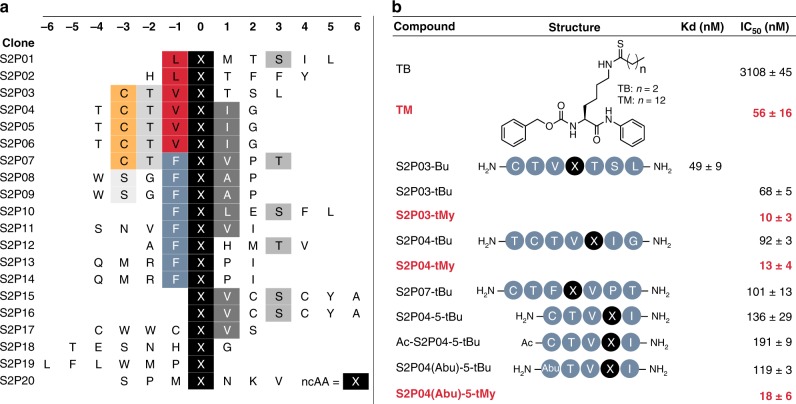
Phage-assisted identification of peptide inhibitors for SIRT2. **a** Amino acid sequences identified through phage selection of peptides that bind to SIRT2. X = N^Ɛ^-butyryl-l-lysine. **b** Binding and inhibition parameters of the selected peptides as N^Ɛ^-butyryl-l-lysine (BuK), N^Ɛ^-thiobutyryl-l-lysine (tBuK), or N^Ɛ^-thiomyristoyl-l-lysine (tMyK) derivatives, along with standard compounds TB and TM. IC_50_ values of tMyK derivatives are highlighted in red. *K*_d_ values are given as the mean ± standard deviation of three individual experiments (*n* = 3). IC_50_ values are given as the mean ± standard deviation of two individual experiments (*n* = 2). Source data are available in the Source Data file.

**Fig. 6:**
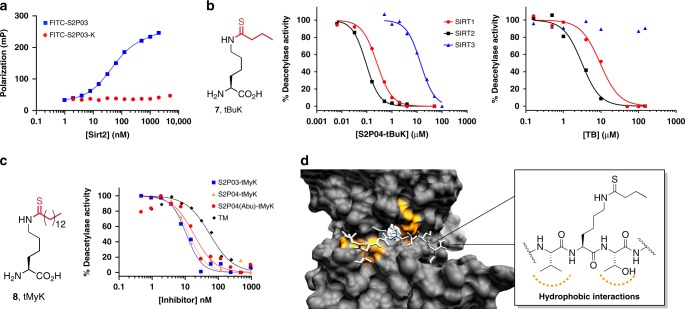
Binding and inhibition of human sirtuins by synthetic ligands derived from selected peptides. **a** Fluorescent polarization assay of SIRT2 binding to FITC-conjugated S2P03 and its deacylated counterpart S2P03-K. Values are reported as the mean of three independent experiments (*n* = 3). **b** Inhibition of human SIRT1–3 by S2P04-tBuK (left) and TB (right). Values are reported as the mean of two independent experiments (*n* = 2). **c** Inhibition of SIRT2 by four tMyK-containing peptides. Values are reported as the mean of two independent experiments (*n* = 2). **d** Interactions between S2P03-tBuK and SIRT2 predicted by molecular dynamic simulation. Data associated with all inhibition and binding curves can be found in the Source Data File.

We produced our BuK-containing phage display library by growing in Top10 cells harboring pEDF-BuKRS and M13KO7(pIII^−^) in the presence of BuK, and subjected the produced phage particles to affinity selection against recombinant human SIRT2 (see Supplementary Fig. 11 for SDS-PAGE). To avoid the catalytic removal of the butyryl modification during selection, we performed selections against apo SIRT2. We isolated phage clones that were eluted after the third round of affinity selection and sequenced their DNA (*n* = 20, Fig. 5a). The sequencing data revealed that the population had converged on consensus sequences primarily involving the residues immediately adjacent to the BuK residue. Consistent with two previous studies, we observed an enrichment of valine and leucine residues at the position immediately N-terminal to BuK (−1 position)^36,37^. We also observed a strong enrichment of phenylalanine at this position. Immediately C-terminal to BuK (+1 position), we observed a strong enrichment of amino acids with aliphatic sidechains with greater than 50% of the isolated clones having an isoleucine, valine, leucine, or alanine at this position. Together, these findings suggest a substrate preference of SIRT2 for sequences with hydrophobic residues next to the modified lysine. Consistent with previous findings, we also observed an enrichment of serine and threonine residues at the +3 position that has been shown to make hydrogen-bonding interactions with Q265 of SIRT2^38^. Of all of the sequenced clones, 25% contained the consensus motif Cys–Thr–^Val^/_Phe_–BuK–^Val^/_Ile_. To assess the contribution of BuK in directing the binding of selected peptides, we synthesized two FITC-conjugated peptides, S2P03 and its deacylated counterpart S2P03-K, and tested their binding to SIRT2 using the fluorescence polarization assay. S2P03 displayed a *K*_d_ value of 49 ± 9 nM that indicated a very tight binding of S2P03 to SIRT2, and was in stark contrast to almost no detectable *K*_d_ for S2P03-K, confirming the essential role of BuK in directing the binding of S2P03 to SIRT2, and also the concept that the active site-directed ligand evolution can conveniently identify potent ligands for a target (Figs. 5b and 6a).

Replacing the acyl-lysine amide moiety of substrates with a slowly hydrolyzing thioamide has proven to be an effective strategy for inhibiting the sirtuin enzymes^39,40^. As such, we chose three selected peptides (S2P03, S2P04, and S2P07) that contained the Cys–Thr–^Val^/_Phe_–BuK–^Val^/_Ile_ motif, and synthesized their counterparts (S2P03-tBuK, S2P04-tBuK, and S2P07-tBuK), in which the original BuK was replaced by *N*^Ɛ^-thiobutyryl-l-lysine (**7**, tBuK in Fig. 6b) and tested their inhibition of SIRT2. All synthetic methods and characterization of the peptides can be found in the Supplementary Notes, Supplementary Figs. 12–19, and Supplementary Table 1. As a reference compound, we utilized TB (its structure shown in Fig. 5b), a recently reported, potent SIRT2-selective inhibitor^31^. To determine IC_50_ values of all three synthesized peptides toward SIRT2, we adopted a recently developed, continuous assay that measured SIRT2-catalyzed hydrolysis of a commercially available fluorogenic substrate, SP-SubSir-01^41^. By fixing concentrations of SIRT2 and SP-SubSir-01, and varying the inhibitor concentration, we determined that all three peptides inhibited SIRT2 significantly better (up to 45-fold) than TB with IC_50_ values ranging from 68 to 101 nM (Fig. 5b, Supplementary Fig. 20). Since the consensus motif found in our selection included only five residues, we also synthesized a truncated version of S2P04 with five consensus residues (S2P04-5-tBuK). S2P04-5-tBuK inhibited SIRT2 with an IC_50_ value of 136 ± 29 nM, slightly higher than that of S2P04. This observation suggests that although the identity of residues distal to the BuK may not be critical, interactions, such as backbone hydrogen bonding, may still contribute to enzyme–ligand binding. Adding an acetyl group to the N terminus of S2P04-5-tBuK diminished the inhibition strength further to an IC_50_ value of 191 ± 9 nM. Due to the concern that the cysteine residue in S2P04-5-tBuK may initiate a dimer formation that complicates the IC_50_ measurement, we also replaced this cysteine residue in S2P04-5-tBuK with l-2-aminobutyric acid (Abu) to afford S2P04(Abu)-5-tBuK, and measured its inhibition of SIRT2. The determined IC_50_ value was 119 ± 3 nM, lower than that for S2P04-5-tBuK, but still higher than that measured for S2P04-tBuK. One advantage of our active site-directed ligand evolution technique is its potential to identify highly selective peptide ligands toward a target. To test this prospect, we characterized S2P04-tBuK selectivity for SIRT2 by measuring its inhibitory properties toward two other closely related sirtuin enzymes, SIRT1 and SIRT3^42^. Expression conditions and SDS-PAGE for SIRT1 and SIRT3 can be found in the “Methods” section and Supplementary information (Supplementary Fig. 21). Our results showed that S2P04-tBuK inhibited SIRT2 2.7- and 152-fold more potently than SIRT1 and SIRT3, respectively, a feature shared by TB (Fig. 6b, Supplementary Table 2). TM is a TB analog that contains *N*^*ε*^*-*thiomyristoyl-l-lysine (**8**, tMyK in Fig. 6c) instead of tBuK. A recent study indicated that TM is tenfold more potent than TB in inhibiting SIRT2 in vitro, and displayed anti-proliferation activity toward various human cancer cells and mouse xenograft models of human breast cancer^31^. TM has an elongated linear fatty acylation chain that penetrates deep into the active site of SIRT2 for improved binding. To explore whether we could integrate tMyK into our selected peptides for achieving stronger SIRT2 inhibition than tBuK-containing peptides, we synthesized S2P03-tMyK, S2P04-tMyK, and S2P04(Abu)-5-tMyK that contained tMyK in the original BuK position, and tested their inhibition on SIRT2 catalysis (see Supplementary Notes and Supplementary Figs. 22–27 for synthesis and characterization of these peptides). All three peptides displayed very high potency against SIRT2 activity with determined IC_50_ values as 10 ± 3 nM for S2P03-tMyK, 13 ± 4 nM for S2P04-tMyK, and 18 ± 6 nM for S2P04(Abu)-5-tMyK (Figs. 5b and 6c). In a similar setup, TM displayed an IC_50_ value as 56 ± 16 nM, 3- to 6-fold less potent than the three tMyK-containing peptides we synthesized. Also, these tMyK-containing peptides showed enhanced selectivity against SIRT1, as S2P03-tMyK inhibited SIRT2 40-fold more efficiently than SIRT1 (Supplementary Fig. 28, Supplementary Table 2). Interestingly, S2P03-tMyK displays relatively fast inhibition kinetics, as testing inhibition without preincubation of enzyme resulted in an insignificant change in IC_50_ (Supplementary Fig. 29). While it would be interesting to observe the binding kinetics over a longer time period with SIRT2, the highly active curves plateaued after 5–10 min of incubation with substrate (Supplementary Fig. 30), which prevented us from performing long-term analysis of the inhibitors. To further understand how our selected peptides interact with SIRT2 for achieving high potency, we carried out molecular dynamics simulations of the binding of the selected thiobutyryl peptides to SIRT2. The simulation results showed that residues flanking the tBuK residue in all peptides involved hydrophobic interactions with residues F235 and L239 of SIRT2 (Fig. 6d, Supplementary Figs. 31 and 32, and Supplementary Movies 1–5), providing a possible explanation for its high potency.

## Discussion

For quick identification of potent ligands of epigenetic regulators, we have developed a phage-assisted, active site-directed ligand evolution technique, as well as an assisting technique, for the efficient production of ncAA-containing phages. As a demonstration of the utility of our techniques, we constructed a phage-displayed, BuK-containing peptide library and used this library for active site-directed ligand evolution of peptides that bind to SIRT2. The selected peptides synthesized with tBuK or tMyK in place of BuK were demonstrated to be much more potent inhibitors of SIRT2 than two control compounds, TB and TM, respectively. In comparison to SirReal1–6, a group of small-molecule sirtuin inhibitors, all our tested peptides have much lower IC_50_ values^43^. Also, S2iL5 is a 14-mer cyclic peptide that showed high potency to inhibit SIRT2^38^. A recent study of using the same SIRT2 inhibition assay, as described in this work, indicated an IC_50_ value as 600 nM that is also significantly higher than those for our synthesized peptides^41^. Collectively, our data demonstrate unequivocally that an amber-obligate phage display library constructed via superinfection-immunity-based selection can be used for active site-directed ligand evolution to identify potent and selective peptide inhibitors for an epigenetic regulator. A number of other similar-functioning ncAAs have been genetically encoded that are compatible with this technique. Thus, adapting our developed technique to these ncAAs will allow for potentially rapid discovery of potent ligands for many different corresponding epigenetic regulators. In theory, a ligand of any target that is not necessarily a protein may be integrated into an ncAA. The genetic incorporation of this ncAA into phages can facilitate a similar type of active site-directed ligand evolution. Implementation of this prospect will have great potentials to advance drug discovery research. Therefore, we believe that our developed techniques will find great applications in the field of drug discovery.

Although we originally developed the superinfection-immunity-based selection technique as a technique assisting the phage-assisted, active site-directed ligand evolution technique, the technique itself is not limited just for this application, and not confined for the construction of peptide libraries. As we have demonstrated, the amber-obligate library generated from superinfection-immunity selection is robust and allows for the efficient incorporation of several ncAAs into phage libraries. Therefore, the constructed libraries can be used for a variety of applications. Larger proteins, such as antibodies, are routinely displayed on Ff phages. Since the superinfection-immunity-based selection relies on an inherent property of Ff phage biology, this method is not limited by library design or size. Thus, it can theoretically be applied to generate libraries of much larger peptides and proteins within the general constraints of pIII display. To date, over 200 ncAAs that contain a large variety of functionalities have been genetically encoded via amber suppression^14,44,45^. The superinfection-immunity-based selection technique provides a means to readily generate phage display libraries containing these diverse ncAAs, for which numerous applications can be envisaged.

## Methods

### Construction of a pADL-(NNK)_6_TAGgIII library

The pADL-(NNK)_6_TAGgIII library was designed in which a gene fragment with its seventh coding position fixed as TAG and the other six randomized as NNK was fused at the gIII 5′ side. We constructed this library by undergoing polymerase chain reaction (PCR) to directly amplify the pADL-10b plasmid using two primers pADL-F: 5′-GGTCCGTCCATGGCCTGCNN KNNKNNKNNK NNKNNKTAGG GCCCGGG-3′, and pADL-R: 5′-CCACGGCCAT GGCCGGCTG GGCCGCG-3′. We digested the PCR product using the NcoI restriction enzyme, and ligated the digested product using T4 DNA ligase. DpnI was also used to remove the template phagemid. We then electroporated the ligated plasmids into competent *E. coli* Top10 cells, incubated the transformants in 1 mL of LB medium, and then inoculated them into 50 mL of LB medium containing 100 µg mL^−1^ ampicillin. After OD_600_ reached 1.0, we collected 0.5 mL of the cell culture, mixed it with 50% glycerol, and stored at −80 °C. Several aliquots are made for the total coverage of more than 10^11^ cfu. To collect the phagemids, we normalized the amount of cell stocks to guarantee an equal amount of phagemids from each aliquot. We isolated 20 clones from this library and subjected them to DNA sequencing. The sequencing data are presented in Supplementary Fig. 1. Among these 20 clones, 16 contained the designed sequences, 2 were the original pADL-10b phagemid, and 2 were deleterious cloning products that resulted possibly from the synthetic errors in the DNA primers. Among all 16 designed clones, all sites are randomized with no obvious enrichment toward certain codons.

### Propagation of the pADL-(NNK)_6_TAGgIII library

The pADL-(NNK)_6_TAGgIII library was used to transform DH5α that contained M13KO7(pIII^−^) (the construction of this plasmid is decribed in the next section). The transformed cells were used to inoculate 100 mL of 2YT containing 100 µg mL^−1^ ampicillin and 25 µg mL^−1^ kanamycin. The culture was grown to OD_600_ 0.5, and 1 mM IPTG was added. The culture was incubated at 37 °C overnight. The next day, the cells were pelleted (20 min, 5251 × *g*), and 2 × 40 mL of the supernatant was added to 10 mL of PEG precipitation solution (2.5 M NaCl, 20%, PEG 8000) and incubated at 4 °C overnight. Then, the phage was pelleted (20 min, 10,976 × *g*), the supernatant was poured off, and the residue was resuspended in 10 mL of PBS, pH 7.4. Residue cells were pelleted (20 min, 5251 × *g*), and the supernatant was transferred to a new tube. In all, 2.5 mL of precipitation solution was added with the phage solution and incubated overnight at 4 °C. The next day, phages were pelleted (20 min, 10,976 × *g*), and the supernatant was poured off. The pellet was resuspended in 4 mL of PBS, and the remaining cells and debris were pelleted (20 min, 5251 × *g*), and the supernatant was transferred to a new tube. The purified phages were heated to 70 °C to kill all residual cells for 15 min and then stored at 4 °C. The phage solution was used to infect ER2738 and repeat the protocols mentioned above for passaging. After six rounds of propagation, we isolated 20 clones from the finally propagated library, and subjected them to DNA sequencing. The sequencing data are also presented in Supplementary Fig. 1. Among these 20 clones, 18 turned out to be the original pADL-10 phagemid and 2 were the desired library clones. Deleterious clones were not observed due to their inability to propagate.

### Description and validation of M13KO7(pIII^−^)

M13KO7(pIII^−^) is a derivative of the commonly used M13KO7 helper phage that contains an in-frame nonsense mutation in gene III, rendering it unable to produce the pIII protein. When used to complement a phagemid bearing a peptide-pIII library, this helper phage allows for polyvalent display of the peptide library on all five copies of pIII. To generate M13KO7(pIII^−^), we introduced a TAA mutation at position K10 of gIII. To construct the M13KO7(pIII^−^) phagemid, we followed a standard Pfu polymerase-based Quik-Change protocol. We used two primers M13K07-g3TAA-F: 5′-gttgaaagtt gtttagcaTa accccataca gaaaattc-3′ and M13K07TAA-R: 5′-gaattttctg tatggggttA tgctaaacaa ctttcaac-3′ to introduce a single TAA mutation at the K10 coding site of gene III to afford the M13KO7(pIII^−^) phagemid.

We performed two experiments to validate M13KO7(pIII^−^) as a helper phage for polyvalent display. To confirm the phenotypic knockout of pIII, we expressed the helper phage in *E. coli* Top10 F′ at 37 °C overnight in 2xYT containing 25 µg mL^−1^ kanamycin. The following day, the cells were pelleted, and the supernatant was incubated at 65 °C for 15 min to kill residual bacteria. The heat-killed supernatant (10 µL) was then spotted onto an overlay of *E. coli* Top10 F′ in top agar on agar containing 10 µg mL^−1^ tetracycline, and incubated at 37 °C overnight. As positive controls, we also expressed and spotted wild-type M13KO7 and CM13 phages (Antibody Design Labs). After overnight incubation, a zone of delayed cell growth was present for the spots corresponding to wild-type M13KO7 and CM13, indicating the presence of viable phages. By contrast, no delayed growth was observed in the spot corresponding to M13KO7(pIII^−^), confirming the loss of functional pIII that is required for host infection (Supplementary Fig. 3).

Next, we confirmed the ability of M13KO7(pIII^−^) to complement a phagemid-bearing pIII and produce viable phages. To do this, we co-transformed *E. coli* Top10 F′ with M13KO7(pIII^–^), and one of two phagemids: pADL-10b or pADLg3-amber. pADL-10b contains the gene encoding wild-type pIII, whereas pADLg3-amber contains wild-type pIII with an in-frame amber mutation. The transformed cells were grown in 2xYT media containing 100 µg mL^−1^ ampicillin and 25 µg mL^−1^ kanamycin to an OD_600_ of 0.8, at which point pIII expression was induced with the addition of IPTG. Following overnight incubation, the supernatants were collected and heat-killed as described above, and 10 µL of the heat-killed supernatants were used to infect 90 µL of log-phase *E. coli* Top10 F′ for 45 min. Infected cultures were spread onto agar selection plates containing 100 µg mL^−1^ ampicillin and grown at 37 °C overnight. No growth was observed for cells that were infected with the supernatant from pADLg3-amber, as both the phagemid and helper phage contain nonsense mutations in pIII. However, plating cells infected with the supernatant from pADL-10b resulted in a dense patch of cell growth, confirming the ability of M13KO7(pIII^−^) to complement phagemid-borne pIII and produce functional phagemid particles (Supplementary Fig. 4).

### Construction of the pADL-(NNK)_7_gIII phagemid library

The phagemid pADL-10b, which contains the gene encoding pIII, was purchased from Antibody Design Labs. To introduce a seven-site library at the N terminus of pIII, the phagemid was PCR-amplified using the primers pADLg3-P1: 5′-CATGCCATGGCC(NNK)_7_GCGGCGAAAGCGG-3′, and pADLg3-P2: 5′-CATGCCATGGCCGGCTGGGCCGC-3′. The PCR product was digested with NcoI, ligated, and used to transform electrocompetent *E. coli* Top10 F′ cells. A small aliquot of the transformed cells was plated onto agar selection plates to quantify the transformation efficiency, and the remaining culture was diluted in fresh 2xYT media and grown at 37 °C overnight. The following day, the cells were harvested by centrifugation, resuspended in one-tenth volume of 2xYT containing 20% glycerol, and aliquots were stored at −80 °C for use in subsequent superinfection-immunity-based selection.

### Generation of the amber-obligate pADL-(NNK)_7_gIII library

For the first round of superinfection-immunity-based selection, Top10F′ cells containing the naive phagemid library were grown in 2xYT media containing 100 µg mL^−1^ ampicillin and 10 µg mL^−1^ tetracycline at 37 °C. Upon reaching OD_600_ ≈0.3, the expression of pIII was induced with the addition of 0.2 mM IPTG. Thirty minutes after induction, the culture was superinfected with CM13 helper phage (Antibody Design Labs, 1 × 10^11^ pfu, MOI ≈15), and incubated at 37 °C for an additional 40 min. Following the superinfection, the cells were pelleted by centrifugation, resuspended in fresh 2xYT media containing 100 µg mL^−1^ ampicillin, 25 µg mL^−1^ kanamycin, and 1 mM IPTG, and grown at 37 °C overnight. The following day, the cells were pelleted, and the plasmids purified from the cells using a commercial plasmid extraction kit. The phagemid library was separated from the helper phage and purified by agarose gel electrophoresis.

Following the first round of superinfection-immunity-based selection, the phagemids were passed through *E. coli* DH5α to remove clones that contained nonfunctional pIII. *E. coli* DH5α containing the M13KO7(pIII^−^) helper phage was transformed with the purified phagemid library, recovered for 1 h in 2xYT media, and then grown overnight in 2xYT containing 100 µg mL^−1^ ampicillin, 25 µg mL^−1^ kanamycin, and 1 mM IPTG. The following day, the cells were pelleted, and phage particles were precipitated from the supernatant on ice with the addition of one-fifth volume of a 5 × Phage Precipitation solution (2.5 M NaCl, 20% PEG 8000). The precipitated particles were pelleted by centrifugation and then resuspended in one-tenth volume of PBS. The resuspension was clarified by centrifugation, and the supernatant was incubated at 65 °C for 15 min to kill residual *E. coli*.

For the second round of superinfection selection, the purified phage particles (3.7 × 10^9^ cfu, MOI ≈ 0.1) were added to a 100-mL culture of the mid log-phase *E. coli* Top10 F′ in 2xYT media supplemented with 10 µg mL^−1^ tetracycline. The culture was incubated for 1 h at 37 °C, and then diluted 1:1 with fresh 2xYT containing 100 µg mL^−1^ ampicillin and 0.5 mM IPTG. The culture was incubated at 37 °C to OD_600_ ≈ 0.4, and then 60 mL was removed and infected with CM13 helper phage (1 × 10^11^ pfu, MOI ≈ 10). The culture was incubated for an additional 45 min before the cells were pelleted and resuspended in 500 mL of fresh 2xYT media supplemented with 100 µg mL^−1^ ampicillin, 25 µg mL^−1^ kanamycin, and 1 mM IPTG. The culture was then incubated at 37 °C overnight, and the following day, the phagemid library was extracted and purified as described above.

### Phagemid particle and phage quantification

For all experiments, phagemid particles and phages were quantified via a colony-forming unit assay. In this assay, serial dilutions of the phage solution were prepared in 2xYT media, and 10 µL of each dilution was added to 90 µL of log-phase *E. coli* Top10 F′. Following addition of the phage dilutions, the culture was incubated at 37 °C for 45 min, and then 10 µL was spotted onto agar selection plates containing either 100 µg mL^−1^ ampicillin (phagemids) or 25 µg mL^−1^ kanamycin (phage), which were incubated at 37 °C overnight. The following day, colonies in each spot were counted, and this number was used to calculate the number of cfu in the solution.

### Illumina sequencing of the amber-obligate library

The two primers used for generation of amplicons are (1) the forward primer: 5′-GCCCAGCCGG CCATG-3′ and (2) the reverse primer: 5′-CGGCCGCTTTCGCCGC-3′. A four-step PCR cycle was used to amplify the library region out of the original phagemid library (see Supplementary Notes for specific PCR conditions). Primers were purchased from Integrated DNA Technologies (Coralville, IA, USA). The amplicons were purified and extracted from a 3% agarose gel according to the GenCatch gel extraction kit. These were then sent to the Genomics and Bioinformatics center at Texas A&M University, where Illumina adapters and barcodes were added onto the amplicons via two PCR steps. The library was then sequenced using Illumina MiSeq paired-end sequencing (2 × 150 bp).

Sequences were analyzed in R, and all scripts are available in the Supplementary Information. Reads were first truncated and filtered according to quality scores using the DADA2 package. All forward reads were truncated after 120 base pairs, while all reverse reads were truncated at 95 base pairs. The expected error of each sequence (*E*_max_) was used to determine the initial quality of reads, and any read with an *E*_max_ > 1 was discarded. Reads containing the two primer regions flanking the library sequence were then spliced out of the initial sequences of both forward and reverse reads. Previously published paired-end processing^46^ was adapted to the R platform—any reads containing more than one mismatch in the primer regions or any mismatch in the library region were discarded. The library regions of reads passing the filters were then analyzed for NNK quality and translated into peptide sequences.

### Expression of phages containing ncAAs

*Expression with phenylalanine derivatives*: To express the amber-obligate phagemid library with phenylalanine derivatives, we used pEDF-PhDRS. Electrocompetent *E. coli* containing M13KO7(pIII^−^) and pEDF-PhDRS was transformed with the amber- obligate phagemid library to yield 6.92 × 10^8^ transformants. Transformed cells were grown at 37 °C in 200 mL of 2xYT media supplemented with 100 μg mL^−1^ ampicillin, 34 μg·mL^−1^ chloramphenicol, and 25 μg·mL^−1^ kanamycin to OD_600_ = 0.4–0.6, at which point 1 mM IPTG and 0.2% arabinose was added, and the culture was split into two flasks. To one flask was added one of the ncAA **1**–**4** to a final concentration of 4 mM. Following induction, the phagemids were expressed at 30 °C for 18–24 h.

After expression, each of the 100-mL cultures was split into two 50-mL tubes, and the cells were pelleted (5251 × *g*, 20 min). The upper 40 mL of the supernatant was poured into new 50-mL tubes, and to each tube was added 10 mL of a 5 × phage precipitation solution (2.5 M NaCl, 20% PEG 8000). The tubes were mixed by inversion, and the phages precipitated at 4 °C overnight. The following day, the phages were pelleted (11,000 × *g*, 25 min), the supernatant was decanted, and both pellets were resuspended in the same 10 mL of PBS, pH 7.5. Residual cells were pelleted (5251 × *g*, 20 min), and the supernatant was transferred to a new tube. The phages were precipitated a second time by the addition of 2.5 mL of phage precipitation solution and incubation at 4 °C overnight. The following day, the phages were pelleted (11,000 × *g*, 25 min), and the pellet was resuspended in 1 mL of PBS. Residual cells were pelleted (16,873 × *g*, 10 min), and any remaining cells were killed by incubating the phage solution at 65 °C for 15 min. To verify successful ncAA incorporation, the purified phages that were expressed in the presence and absence of ncAA were quantified using the cfu assay described above.

*Expression with lysine derivatives*: For expression of phages containing **5** and **6** electrocompetent *E. coli* Top10 containing M13KO7(pIII^−^) and pEDF-BuKRS, they were transformed with the purified amber-obligate phagemid library yielding 3.1 × 10^9^ transformants. Phages were expressed and purified exactly, as described for the phenylalanine derivatives, but with the addition of 5 mM nicotinamide to the growth media.

### Expression of TEV protease

His_6_-TEV(S219V) protease was expressed and purified essentially as described previously^47^ with minor modifications. Briefly, *E. coli* BL21(DE3) containing the plasmid encoding TEV protease was grown in 2xYT to an OD_600_ = 0.6, at which point, 1 mM IPTG was added, and the protein was expressed at 30 °C. Four hours after induction, the cells were pelleted (5251 × *g*, 20 min), and the cell pellets were stored at −80 °C until purification.

TEV protease was purified via nickel affinity chromatography. The cell pellets were resuspended in lysis buffer (50 mM NaH_2_PO_4_, 300 mM NaCl, and 10 mM imidazole, pH 8.0), and sonicated. The soluble fraction was recovered by centrifugation (10,976 × *g*, 50 min), and the clear supernatant was incubated with 2 mL of Ni^2+^-NTA resin with end-over-end rotation for 1 h at 4 °C. The resin was then washed with 40 mL of the lysis buffer, and the protein was eluted from the column with an elution buffer (50 mM NaH_2_PO_4_, 300 mM NaCl, and 250 mM imidazole, pH 8.0). The eluted protein was dialyzed against PBS and concentrated via ultrafiltration (10,000 Da MWCO). The purified proteins were analyzed via 12% SDS-PAGE and electrospray ionization mass spectrometry. Following elution from the column, the protein was dialyzed overnight against TEV storage buffer (25 mM Tris, pH 8.0, 100 mM NaCl, 10% glycerol, and 1 mM DTT), concentrated to 15 µM, flash frozen, and stored at −80 °C. SDS-PAGE analysis of purified TEV protease is shown in Supplementary Fig. 8.

### Biotinylation of TEV protease

To generate biotin-labeled TEV protease, aliquots of the purified protein, in storage buffer, were combined and dialyzed against PBS, pH 7.6, overnight at 4 °C. The following day, the protein was dialyzed against PBS for an additional 1.5 h. The pH was adjusted to 8.0 using 0.1 M NaOH, and the protein was concentrated to 10 µM. The concentrated protein was biotinylated with a tenfold molar excess of Ez-Link^TM^ NHS-Biotin (Thermo Fisher Scientific) for 1 h at room temperature with end-over-end rotation. After 1 h, the excess biotin reagent was removed by ultrafiltration (10,000 Da MWCO) by performing tenfold dilutions in PBS, and then concentrating to 1 mL (3 ×). Aliquots of the biotinylated protein were flash frozen and stored at −80 °C.

### Affinity selection against TEV protease

Streptavidin-coated magnetic beads (100 µL, 50% slurry, Genscript) were washed with PBS, pH 7.4 (2 × 1 mL), and then resuspended in 50 µL of PBS. In all, 5 µg of biotin-labeled TEV protease in 400 µL of PBS was added, and the protein/bead mixture was incubated for 15 min at room temperature. After 15 min, the beads were washed with PBS (3 × 1 mL), resuspended in 300 µL of PBS, and then 150 µL of 2 × blocking buffer was added to the tubes (PBS, pH 7.4, 0.3% Tween-20, and 3% bovine srBSA). At the same time, 0.5 mL of 2 × blocking buffer was also added to the purified phagemid library in 1 mL of PBS. Both tubes were incubated at room temperature with end-over-end rotation for 30 min. After 30 min, the blocking buffer was removed from the tube with the beads, and the blocked phage library (0.75 mL) was added. The bead/library mixture was incubated at room temperature with end-over-end rotation for 30 min. After 30 min, the supernatant was removed, and the beads were washed (8 × 1 mL of PBS containing 0.1% Tween-20; 2 × 1 mL of PBS). Bound phages were eluted from the beads by incubating with 100 µL of elution buffer (50 mM glycine, pH 2.2) for 10 min. The elution was then immediately added to 50 µL of neutralization buffer (1 M Tris, pH 8.0).

A small portion (10 μL) of the eluted phages was used for tittering, and the remainder was amplified by adding the elution to a 20- mL culture of *E. coli* Top10 F′ (OD_600 _= 0.45–0.55) in 2xYT media supplemented with 10 μg mL^−1^ tetracycline, and incubated at 37 °C, for 45 min. After 45 min, the cells were pelleted (3724 × *g*, 20 min), resuspended in 300 mL of fresh 2xYT media supplemented with 100 μg mL^−1^ ampicillin, and grown overnight at 37 °C. The following day, the phagemids were extracted using a commercial plasmid Miniprep kit. The purified phagemids were used to transform electrocompetent *E. coli* Top10 containing pEVOL-PylT-PylRS(N346A/C348A) and M13KO7(pIII^−^). After electroporation, the transformed cells were recovered in 1 mL of 2xYT supplemented with 35 μg mL^−1^ chloramphenicol and 25 μg mL^−1^ kanamycin for 1 h at 37 °C. A small portion of the recovered cells were tittered to ensure that the number of transformed cells exceeded the number of eluted phages by at least 100-fold. After 1 h, the recovered cells were added to 50 mL of 2xYT media supplemented with ampicillin, chloramphenicol, and kanamycin, and amplified overnight. This amplified culture was used to prepare glycerol stocks for expressing phages for further rounds of selection according to the methods described above.

### Affinity selection against streptavidin

Selection was performed against streptavidin that had been nonspecifically adsorbed to a polystyrene petri dish. To immobilize the target protein, lyophilized streptavidin (Chem-Impex International, Woodale, IL) was dissolved in 0.1 M NaHCO_3_, pH 8.6, to a final concentration of 25 μg mL^−1^. In all, 1.5 mL of the protein solution was added to a sterile petri dish (60 × 15 mm, Falcon 35-3004) and incubated at 4 °C overnight in a sealed, humidified, box. The following day, the protein-coating solution was removed, the petri dish was filled with blocking buffer (0.1 M NaHCO_3_, 5 mg mL^−1^ BSA, and 0.1 mg mL^−1^ streptavidin, pH 8.6), and the plate was incubated for 2 h at 4 °C. Following blocking, the plate was washed six times with 2 mL of PBST (PBS + 0.1% Tween-20, pH 7.5), and the phage library, in 1 mL of PBS, was added to the dish. The phages were incubated with the target at room temperature in a humidified box for 1 h (rounds 1–3) or 30 min (round 4). After incubating the library with the target, unbound phages were removed, and the dish was washed ten times by completely filling the dish with PBST, swirling for 30 s, pouring off the PBST, and then slapping the dish face-down on a clean paper towel. The bound phages were then eluted with the addition of 1 mL of 0.1 mM d-biotin in PBS incubating at room temperature for 30 min. Eluted phages were quantified and amplified exactly as described for the selection against TEV protease.

### Expression of SIRT2

The plasmid pHEX-His_6_-SIRT2 was a kind gift from Professor John Denu at the University of Wisconsin School of Medicine and Public Health. *E. coli* Top10 containing the plasmid pHEX-His_6_-SIRT2 was grown at 37 °C in 2 L of 2xYT containing 100 µg mL^−1^ ampicillin to OD_600_ of 0.6, at which point SIRT2 expression was induced by the addition of 0.1 mM IPTG. Two hours after induction, the cells were harvested by centrifugation (5251 × *g*, 20 min), and the cell pellets were stored at −80 °C until purification.

Cell pellets were thawed and resuspended in 40 mL of lysis buffer (50 mM NaH_2_PO_4_, pH 8.0, 300 mM NaCl, 10 mM imidazole, and 0.1 mM phenylmethanesulfonyl fluoride (PMSF)). The resuspended pellet was incubated in the presence of lysozyme (1 mg mL^−1^, chicken egg white) for 30 min on ice. Following incubation, the resuspension was sonicated twice (1 s on, 1 s off, 1 min total, 60% output) and clarified by centrifugation (10,976 × *g*, 45 min). To the clarified supernatant was added 4 mL of a 50% slurry of high-affinity Ni^2+^-charged resin (GenScript), and the mixture was incubated at 4 °C with end-over-end rotation for 30 min. After 30 min, the mixture was filtered through a disposable column, the resin was washed with 40 mL of lysis buffer, and the bound protein was eluted with 7 mL of elution buffer (lysis buffer containing 250 mM imidazole). Subsequent purification steps vary depending on whether the protein was used for biotinylation and phage panning or SIRT2 inhibition assays.

For phage panning, the eluted protein was concentrated to 1 mL by ultrafiltration (10,000 Da MWCO), diluted 1:9 with Q Sepharose buffer A (50 mM NaH_2_PO_4_, pH 8.0, 50 mM NaCl, and 0.1 mM DTT), and concentrated again to 1 mL. The concentrated sample was applied to a 25-mL Q Sepharose column (GE Healthcare) that was pre-equilibrated with Q Sepharose buffer A. The column was washed with 2 column volumes (CV, 2 mL min^−1^) of buffer A, and the proteins were eluted with a linear gradient of 0–100% buffer B (50 mM NaH_2_PO_4_, pH 8.0, 1 M NaCl, and 0.1 mM DTT) over 3 CVs (2 mL min^−1^). Fractions from the main peak were combined and concentrated to 1 mL for biotinylation. SDS-PAGE of SIRT2 purified using this protocol is shown in Supplementary Fig. 11a.

For inhibition assays, the eluted protein was loaded onto a HiPrep 26/10 desalting column (GE Healthcare) that had been pre-equilibrated with Source 15Q buffer A (20 mM Tris, pH 8.0, 50 mM NaCl, 0.2 mM DTT, and 10% glycerol). The desalted protein was concentrated by ultrafiltration to 1 mL (10,000 Da MWCO) and applied to a 10-mL Source 15Q column (GE Healthcare) pre-equilibrated with Source 15Q buffer A. The column was washed with 3 CVs (2 mL min^−1^) of Source 15Q buffer A, and then eluted with a linear gradient from 0 to 60% Source 15Q buffer B (20 mM Tris, pH 8.0, 1 M NaCl, 0.2 mM DTT, and 10% glycerol, 1 mL min^−1^) over 5 CVs. The fractions containing pure SIRT2 were concentrated to 40–70 µM, flash frozen in 10- µL aliquots, and stored at −80 °C. Enzyme concentration was determined from the absorbance at 280 nm using the calculated extinction coefficient of 32,470 M^−1^ cm^−1^. SDS-PAGE of SIRT2 purified using this protocol is shown in Supplementary Fig. 11b.

### Biotinylation of SIRT2

SIRT2 (53 µM) was biotinylated with a tenfold molar excess of EZ-Link^TM^ NHS-Biotin (Thermo Fisher Scientific) for 2 h on ice with gentle rocking. After 2 h, the solution was directly loaded onto a Superdex 75 10/300 GL size-exclusion column (GE Healthcare) pre-equilibrated with 50 mM Tris, pH 8.0, 250 mM NaCl, 0.2 mM DTT, and 10% glycerol. The eluted protein was concentrated by ultrafiltration (10,000 Da MWCO) to 10 µM, flash frozen in 10-µL aliquots, and stored at −80 °C. Biotinylation of the protein was confirmed in two ways. In method one, biotin was detected via Western blot using a streptactin–horseradish peroxidase conjugate (Bio-Rad). In method two, biotinylation was confirmed by capture on magnetic streptavidin beads (GenScript) using 2 µg of biotinylated SIRT2 as described previously^48^. Briefly, biotinylated SIRT2 (2 µg) was diluted in 50 µL of PBS and added to streptavidin-coated magnetic beads (New England BioLabs) that had been washed with PBS (3 × 1 mL). The protein and beads were then incubated for 20 min at room temperature with rotation. Following incubation, the supernatant was removed, and the beads were washed with PBS containing 0.1% Tween (4 × 1 mL). The supernatant and beads were then directly used for the SDS-PAGE analysis shown in Supplementary Fig. 11.

SDS-PAGE of the streptavidin pulldown is shown in Supplementary Fig. 11c. Enzymatic activity of the biotinylated SIRT2 was confirmed using the continuous sirtuin assay provided below.

### Affinity selection against SIRT2

*Rounds 1 and 3*: Streptavidin-coated magnetic beads (100 µL, 50% slurry, GenScript) were transferred to a 1.5-mL tube, washed twice with 1 mL of phosphate-buffered saline (PBS, 137 mM NaCl, 2.7 mM KCl, 10 mM Na_2_HPO_4_, and 1.8 mM KH_2_PO_4_, pH 7.5), resuspended in 100 µL of PBS, and split into two tubes. Biotinylated SIRT2 (10 µg) in 400 µL of PBS was added to one of the tubes, and an equal volume of PBS was added to the other tube (hereafter referred to as +SIRT2 and −SIRT2, respectively). The bead/protein mixture was incubated with rocking for 20 min at room temperature. After 20 min, the supernatant was removed, 1 mL of blocking buffer (0.1 M NaHCO_3_, pH 8.6, 5 mg mL^−1^ BSA) was added, and the tubes were incubated at room temperature with end-over-end rotation. After 30 min, the blocking buffer was removed from the −SIRT2 tube, the resin was washed with PBST (3 × 1 mL; PBS containing 0.1% Tween-20) followed by PBS (1 × 1 mL), and the purified phage library in PBS (Round 1: 6.2 × 10^10^ cfu, Round 3: 2.9 × 10^10^ cfu) was added to the tube for negative selection. The phage bead mixture was incubated for 30 min at room temperature with end-over-end rotation. After 30 min, the +SIRT2 resin was washed (3 × 1 mL of PBST, 1 × 1 mL of PBS), and the phage library was transferred to the +SIRT2 tube and incubated for 30 min at room temperature with end-over-end rotation. After 30 min, the phage library was removed, and the resin was washed (10 × 1 mL of PBST) to remove nonspecifically bound phages. During each washing step, the resin was completely resuspended by pipetting up and down. To remove phages binding to the polypropylene tube, the resin was transferred to a new tube after the third, sixth, and ninth washes. After the last wash, phages were eluted by incubating for 10 min with 100 µL of a low pH elution buffer (50 mM glycine, pH 2.2). After 10 min, the supernatant was removed and immediately added to 50 µL of neutralization buffer (1 M Tris, pH 8). The neutralized elution was immediately used for amplification.

*Rounds 2 and 4*: Eight wells of a pre-blocked neutravidin-coated polystyrene plate (Pierce) were washed with PBS (3 × 250 µL). Biotinylated SIRT2 (1.25 µg in 100 µL of PBS) was added to each of the wells, and the plates were incubated in a sealed, humidified box at room temperature for 30 min. After 30 min, the SIRT2 solution was removed, and the wells were washed (3 × 250 µL of PBST, 1 × 250 µL of PBS). The phage library in PBS (800 µL, 5.4 × 10^9^ cfu) was divided evenly into each of the wells and incubated for 30 min at room temperature in a sealed humidified box. After 30 min, the phages were removed, and the wells were washed (10 × 250 µL of PBST). The bound phages were eluted with low pH elution buffer (100 µL per well) for 10 min. The pooled elution fractions were added to 400 µL of neutralization buffer and immediately used for amplification.

*Phage amplification*: A small aliquot (10 µL) of the phage elution was removed for tittering. The remaining phages were added to an actively growing culture of *E. coli* Top10 F′ (OD_600_ = 0.5–0.6) in 20 mL of 2xYT containing 10 µg mL^−1^ tetracycline for 45 min at 37 °C with rotation. After 45 min, the cells were pelleted (3724 × *g*, 15 min), resuspended in 100 mL of 2xYT containing 100 µg mL^−1^ ampicillin, and amplified overnight at 37 °C. The following day, phagemids were extracted from the amplified culture using a commercial plasmid purification kit. The purified phagemids were used to transform electrocompetent *E. coli* Top10 containing M13KO7(pIII^−^) and pEDF-BuKRS for expressing phages for the next round. For each round, the number of transformants was at least 1000-fold greater than the number of eluted phages.

### Expression of SIRT1

The plasmid pQE80-His_6_-SIRT1 was a kind gift from Professor John Denu at the University of Wisconsin School of Medicine and Public Health. *E. coli* BL21(DE3) containing the plasmid pQE80-His_6_-SIRT1 was grown at 37 °C in 3 L of 2xYT media supplemented with 100 µg mL^−1^ ampicillin to an OD_600_ of 0.7, at which point, protein expression was induced with the addition of 0.5 mM IPTG. Four hours post induction, the cells were harvested by centrifugation (3724 × *g*, 20 min), and cell pellets were stored at −80 °C until purification. Cell pellets were resuspended in 50 mL of lysis buffer (50 mM Tris, pH 8.0, 250 mM NaCl, 10 mM imidazole, 0.1 mM PMSF, and 1% Triton X-100) and incubated with 1 mg mL^−1^ lysozyme for 30 min on ice. Following incubation, the resuspension was sonicated twice (1 s on, 1 s off, 1 min total, 60% output) and clarified by centrifugation (10,976 × *g*, 45 min). To the clarified supernatant was added 4 mL of a 50% slurry of high-affinity Ni-charged resin (GenScript), and the mixture was incubated at 4 °C with end-over-end rotation for 30 min. After 30 min, the mixture was filtered through a disposable column; the resin was washed with 40 mL of lysis buffer containing 0.1% Triton X-100, followed by 40 mL of lysis buffer containing 0% Triton X-100. The bound protein was eluted with 7 mL of elution buffer (lysis buffer containing 250 mM imidazole). The eluted protein was then desalted and purified on a Source 15Q column exactly as described for SIRT2. The fractions containing SIRT1 were concentrated to 19 µM, flash frozen in 25-µL aliquots, and stored at −80 °C. Enzyme concentration was determined from the absorbance at 280 nm using the calculated extinction coefficient of 40,340 M^−1^ cm^−1^. SDS-PAGE of SIRT1 purified using this protocol is shown in Supplementary Fig. 21a.

### Expression of GST-SIRT3

*E. coli* Top10 containing the plasmid pGEX4T-GST-SIRT3, a kind gift from Professor John Denu at the University of Wisconsin School of Medicine and Public Health, was grown at 37 °C in 2 L of 2xYT media supplemented with 100 µg mL^−1^ ampicillin to an OD_600_ of 0.6, at which point, protein expression was induced with the addition of 0.2 mM IPTG. Two hours post induction, the cells were harvested by centrifugation (3724 × *g*, 20 min), and cell pellets were stored at −80 °C until purification. Cell pellets were resuspended in 50 mL of lysis buffer (50 mM Tris, pH 7.4, 150 mM NaCl, 0.1 mM PMSF, and 1% Triton X-100) and incubated in the presence of 1 mg mL^−1^ of lysozyme for 30 min on ice. Following incubation, the resuspension was sonicated twice (1 s on, 1 s off, 1 min total, 60% output) and clarified by centrifugation (10,976 × *g*, 45 min). To the clarified supernatant was added 3 mL of a 50% slurry of immobilized glutathione resin (Genscript), and the mixture was incubated at 4 °C for 30 min with end-over-end rotation. After 30 min, the mixture was filtered through a disposable column, and the resin was washed with 50 mL of lysis buffer containing 0.1% Triton X-100. After washing, the bound protein was eluted with 10 mL of lysis buffer containing 0% Triton X-100 and 10 mM of reduced glutathione. The elution was directly applied to a HiPrep 26/10 desalting column (GE Healthcare) that had been pre-equilibrated with 50 mM Tris, pH 7.5, 150 mM NaCl, 0.2 mM DTT, and 10% glycerol. The desalted protein was concentrated to 16 µM via ultrafiltration (10,000 Da MWCO), flash frozen, and stored in 25-µL aliquots at −80 °C. Enzyme concentration was determined from the absorbance at 280 nm using the calculated extinction coefficient of 80,790 M^−1^ cm^−1^. SDS-PAGE analysis of GST-SIRT3 purified using this protocol is shown in Supplementary Fig. 21b.

### FP assay of SIRT2 binding to S2P03 derivatives

SIRT2 was serially diluted in assay buffer (50 mM Tris, 137 mM NaCl, 2.7 mM KCl, 1 mM MgCl_2_, 1 mM DTT, and 0.01% Triton X-100, pH 8.0) in final concentrations ranging from 5 µM to 1 nM, and 24 µL were aliquoted into a black 384-well plate. Peptides S2P03-BuK and S2P03-K were dissolved in DMSO (2.5 mM), diluted with assay buffer to 250 nM, and 1 µL was added to each solution of SIRT2. The plate was incubated at 37 °C for 15–30 min, and then the fluorescence polarization was measured (*λ*_ex_ = 485 nm, *λ*_em_ = 525 nm) using a Biotek Synergy H1 microplate reader.

### SIRT1, 2, and 3 inhibition assay

The continuous assay for comparing isoform specificity of S2P04 was performed using the universal fluorogenic sirtuin substrate described by Schuster et al.^41^. Briefly, the inhibition reactions were performed in 50-µL volumes at 37 °C in black, half-area, 96-well plates. The sirtuin enzyme (0.25 µM) was preincubated with different concentrations of the peptide S2P04 (0.0064–50 µM for SIRT1 and SIRT2, 0.5–100 µM for SIRT3) or TB (0.16–150 µM for SIRT1–SIRT3), and 1 mM NAD^+^ in assay buffer (20 mM Tris, pH 7.8, 150 mM NaCl, 5 mM MgCl_2_, and 1 mM DTT) for 15 min. Following incubation, the reaction started with the addition of the fluorogenic substrate (5 µM), and product formation was monitored by measuring the fluorescence intensity every 20 s for 5 min (SIRT2) or 30 min (SIRT1 and SIRT3) in a BioTek Synergy H1 microplate reader at *λ*_ex_ = 320 nm and *λ*_em_ = 408 nm (gain = 130 and read height = 7 mm). Initial rates of product formation were determined by a linear regression of the plot of fluorescence intensity vs. time and normalized relative to 0% (max inhibitor) and 100% (no inhibitor) controls. The assay was repeated to determine the IC_50_ values of all BuK peptides for SIRT2. IC_50_ values were determined by nonlinear regression of the plot of the normalized initial rate vs. inhibitor concentration using GraphPad Prism. The determined IC_50_ values are provided in Supplementary Table 2, and inhibition curves can be seen in Supplementary Fig. 20.

### SIRT2 inhibition assay for thiomyristoyl peptides

The inhibition assay for thiomyristoyl-containing peptides and TM (purchased from CSNpharm, Cat. No. CSN20494) was performed using a universal continuous sirtuin substrate^41^. Assays were performed in 40-µL volumes at 37 °C in black, 384-well plates. About 10 nM of SIRT2 was preincubated with different concentrations of peptide or TM (0.5–1000 nM in DMF, 20% DMF final concentration) for 15 min in assay buffer (20 mM Tris, pH 7.8, 150 mM NaCl, 5 mM MgCl_2_, 1 mM DTT, and 1 mM NAD^+^). Following incubation, the reaction started with the addition of the fluorogenic substrate (5 µM), and product formation was monitored by measuring the fluorescence intensity every 20 s for 5 min (SIRT2) in a BioTek Synergy H1 microplate reader at *λ*_ex_ = 320 nm and *λ*_em_ = 408 nm (gain = 130 and read height = 7 mm). Initial rates were determined using the plot of fluorescence vs. time for linear portions of each curve. IC_50_ values were then determined using GraphPad Prism to fit a nonlinear regression to the normalized rates vs. inhibitor concentration.

### SIRT2 inhibition assay—no preincubation

The inhibition assay was performed using a universal continuous sirtuin substrate^41^. Assays were performed in 40-µL volumes at 37 °C in black, 384-well plates. Five micrometers of substrate was incubated with different concentrations of peptide or TM (0.5–1000 nM in DMF, 20% DMF final concentration) for 2 min in assay buffer (20 mM Tris, pH 7.8, 150 mM NaCl, 5 mM MgCl_2_, 1 mM DTT, and 1 mM NAD^+^). Following incubation, the reaction started with the addition of SIRT2 (10 nM), and product formation was monitored by measuring the fluorescence intensity every 20 s for 10 min (SIRT2) in a BioTek Synergy H1 microplate reader at *λ*_ex_ = 320 nm and *λ*_em_ = 408 nm (gain = 130 and read height = 7 mm). Initial rates were determined using the plot of fluorescence vs. time for linear portions of each curve. The IC_50_ value was determined using GraphPad Prism to fit a nonlinear regression to the normalized rates vs. inhibitor concentration (Supplementary Fig. 30).

### Molecular docking of peptides binding to SIRT2

*General information*: The protein environments after the reaction of the peptide with NAD^+^ were studied by simulating the different peptides interacting in the position of an analogous inhibitor based on the crystal structure PDB 4X3O. The Schrödinger (Release 2017-4)^49^ and Desmond^50,51^ software was used for all simulations. Each of the simulations for investigating the protein–peptide interactions followed the steps described below.

*Protein preparation module*: Several structures of SIRT2 were available in the PDB. By overlaying these entries, it was evident that SIRT2 is a conformationally flexible protein due to its many loops (Supplementary Fig. 31). However, the domain where the peptide inhibitors bind is a somewhat inflexible region. The 4X3O crystal structure was chosen as a template structure because it contained an analogous thiomyristoyl-lysine peptide, which was modified to the target peptides (S2P03, S2P04, S2P07, S2P04-5, and Ac-S2P04-5), and because it had the fewest missing residues with the highest resolution among the crystal structures available. The Protein Preparation Wizard in the Schrödinger software was used to prepare the 4X3O.pdb structure for simulation at a pH of 7.5 ± 0^52,53^. Crystal structure waters were maintained. The PrimeLoop algorithm was used to fill in missing residues (Met299–Gly304)^53–55^. After initial preparation, the H-bonding network was optimized using the PROPKA tool at a pH of 7.5, water molecule orientations were sampled, and a restrained minimization was performed on all atoms using the OPLS3 force field^56^.

*System builder module*: *Preparation of the molecular dynamics simulations*. The System Builder tool within the Desmond Module (note that this is in the Schrödinger Software Maestro interface distributed with Desmond) was used to prepare the model for a Desmond MD simulation. Modifications by transforming the native thiomyristoyl-lysine peptide into the target peptides were made before applying the solvent box. An orthorhombic solvent box was constructed with a distance of 10.0 Å around the peptide–protein assembly. The solvent box was size-minimized before applying explicit water molecules to the system. The TIP4P^57,58^ water model was used for the explicit water molecules, and the OPLS3^56^ force field for all other atoms. The protein–peptide assembly was charge neutralized using a physiological NaCl concentration of 0.15 M.

*Desmond module*: *Molecular dynamics simulations*. The Desmond multisim molecular dynamics protocol^50,51^ was used for all runs using the NPT ensemble (1.0135 bar, 310.15 K) for a 10-ns production run taking a snapshot every 10 ps, resulting in a trajectory with 1000 frames. The system was put through a relaxation protocol before the production run, as implemented in the Desmond Schrödinger software Maestro interface, and outlined as follows: (1) restrained minimization steps. (2) Brownian dynamics NVT, *T* = 10 K, small timesteps, and restraints on solute-heavy atoms, 100 ps. (3) NVT, *T* = 10 K, small timesteps, and restraints on solute-heavy atoms, 12 ps. (4) NPT, *T* = 10 K, and restraints on solute-heavy atoms, 12 ps. (5) NPT and restraints on solute-heavy atoms, 12 ps. (6) NPT and no restraints, 24 ps. (7) Production run.

Protein–peptide interaction charts: The MD-simulation results were imported, and ligand interaction tables were then generated using the ligand interaction diagram module in the Desmond distributed Schrödinger software. The hydrophobic protein–ligand interactions are tabulated in Supplementary Fig. 32 for the different peptides. These plots show only the hydrophobic interactions stemming from the residues adjacent to the thBuK residue in the peptide inhibitor. The accompanying plots show in which of the 1000 frames the interactions occur (orange bar)^50,51^.

### Synthetic methods

All synthetic routes and characterization for the reported compounds and peptides can be found in the Supplementary Notes.

### Reporting summary

Further information on research design is available in the Nature Research Reporting Summary linked to this article.

## Supplementary information

Supplementary Movie 1

Supplementary Movie 2

Supplementary Movie 3

Supplementary Movie 4

Supplementary Movie 5

Supplementary Information

Reporting Summary

Description of Additional Supplementary Files

## Data Availability

The data that support the findings of this study are available from the corresponding author upon reasonable request. Novel plasmids are available through Addgene with the following identification numbers shown in parentheses: pEVOL-pylT-N346A/C348A (127411), M13K07-g3TAA (127414), and pEDF-PhdRS (127445). The raw next-generation sequencing data of the amber-obligate phage library have been uploaded to NCBI’s Sequence Read Archive. The accession number for the bioproject is PRJNA606283 and for the biosample is SAMN14088869. The source data underlying Figs. 4, 5, and 6a, c and Supplementary Figs. 28 and 29 are provided as a Source Data file.

## Source Data

Source Data

## Code availability

The R Scripts for analysis of the next-generation sequencing results are available in the Supplementary Information. For ease of access, these scripts have also been uploaded to github, and can be found under jhampton1/NNK7-Amber-Analysis.

## References

[1] Liu R, Li X, Lam KS (2017). Combinatorial chemistry in drug discovery. Curr. Opin. Chem. Biol..

[2] Janzen WP (2014). Screening technologies for small molecule discovery: the state of the art. Chem. Biol..

[3] Guha R (2013). On exploring structure-activity relationships. Methods Mol. Biol..

[4] Metivier JP, Cuissart B, Bureau R, Lepailleur A (2018). The pharmacophore network: a computational method for exploring structure-activity relationships from a large chemical data set. J. Med. Chem..

[5] Bhaumik SR, Smith E, Shilatifard A (2007). Covalent modifications of histones during development and disease pathogenesis. Nat. Struct. Mol..

[6] Ellis L, Atadja PW, Johnstone RW (2009). Epigenetics in cancer: targeting chromatin modifications. Mol. Cancer Ther..

[7] Porter NJ, Christianson DW (2019). Structure, mechanism, and inhibition of the zinc-dependent histone deacetylases. Curr. Opin. Struct. Biol..

[8] Musselman CA, Lalonde ME, Cote J, Kutateladze TG (2012). Perceiving the epigenetic landscape through histone readers. Nat. Struct. Mol..

[9] Richon VM (2006). Cancer biology: mechanism of antitumour action of vorinostat (suberoylanilide hydroxamic acid), a novel histone deacetylase inhibitor. Br. J. Cancer.

[10] Gallinari P (2007). HDACs, histone deacetylation and gene transcription: from molecular biology to cancer therapeutics. Cell Res..

[11] Falkenberg KJ, Johnstone RW (2014). Histone deacetylases and their inhibitors in cancer, neurological diseases and immune disorders. Nat. Rev. Drug Discov..

[12] de Ruijter AJ (2003). Histone deacetylases (HDACs): characterization of the classical HDAC family. Biochem. J..

[13] Wang L, Brock A, Herberich B, Schultz PG (2001). Expanding the genetic code of *Escherichia coli*. Science.

[14] Wan W, Tharp JM, Liu WR (2014). Pyrrolysyl-tRNA synthetase: an ordinary enzyme but an outstanding genetic code expansion tool. Biochim. Biophys. Acta.

[15] Neumann H, Peak-Chew SY, Chin JW (2008). Genetically encoding N^ε^-acetyllysine in recombinant proteins. Nat. Chem. Biol..

[16] Nguyen DP (2009). Genetically encoding N^ε^-methyl-l-lysine in recombinant histones. J. Am. Chem. Soc..

[17] Kim CH (2012). Site-specific incorporation of N^ε^-crotonyllysine into histones. Angew. Chem. Int. Ed. Engl..

[18] Huang Y (2010). Genetic incorporation of an aliphatic keto-containing amino acid into proteins for their site-specific modifications. Bioorg. Med. Chem. Lett..

[19] Lee YJ (2013). A genetically encoded acrylamide functionality. ACS Chem. Biol..

[20] Wang ZA (2017). A versatile approach for site-specific lysine acylation in proteins. Angew. Chem..

[21] Wang ZA (2017). A genetically encoded allysine for the synthesis of proteins with site-specific lysine dimethylation. Angew. Chem..

[22] Gattner MJ, Vrabel M, Carell T (2013). Synthesis of N^ε^-propionyl-, N^ε^-butyryl-, and N^ε^-crotonyl-lysine containing histone H3 using the pyrrolysine system. Chem. Commun..

[23] Wang WW (2016). A chemical biology approach to reveal Sirt6-targeted histone H3 sites in nucleosomes. ACS Chem. Biol..

[24] Liu CC, Choe H, Farzan M, Smider VV, Schultz PG (2009). Mutagenesis and evolution of sulfated antibodies using an expanded genetic code. Biochemistry.

[25] Eggertsson G, Soll D (1988). Transfer ribonucleic acid-mediated suppression of termination codons in *Escherichia coli*. Microbiol. Rev..

[26] Boeke JD, Model P, Zinder ND (1982). Effects of bacteriophage f1 gene III protein on the host cell membrane. Mol. Genom. Genet..

[27] Riechmann L, Holliger P (1997). The C-terminal domain of TolA is the coreceptor for filamentous phage infection of E-coli. Cell.

[28] Wang YS (2013). Genetic incorporation of twelve meta-substituted phenylalanine derivatives using a single pyrrolysyl-tRNA synthetase mutant. ACS Chem. Biol..

[29] Wang YS (2012). A rationally designed pyrrolysyl-tRNA synthetase mutant with a broad substrate spectrum. J. Am. Chem. Soc..

[30] Smith BC, Hallows WC, Denu JM (2008). Mechanisms and molecular probes of sirtuins. Chem. Biol..

[31] Jing H (2016). A SIRT2-selective inhibitor promotes c-Myc oncoprotein degradation and exhibits broad anticancer activity. Cancer Cell.

[32] Hoffmann G, Breitenbucher F, Schuler M, Ehrenhofer-Murray AE (2014). A novel sirtuin 2 (SIRT2) inhibitor with p53-dependent pro-apototic activity in non-small cell lung cancer. Biol. Chem..

[33] Cheon MG, Kim W, Choi M, Kim JE (2015). AK-1, a specific SIRT2 inhibitor, induces cell cycle arrest by downregulating Snail in HCT116 human colon carcinoma cells. Cancer Lett..

[34] Herskovits AZ, Guarente L (2013). Sirtuin deacetylases in neurodegenerative diseases of aging. Cell Res..

[35] Lavu S, Boss O, Elliott PJ, Lambert PD (2008). Sirtuins—novel therapeutic targets to treat age-associated diseases. Nat. Rev. Drug. Discov..

[36] Morimoto J, Hayashi Y, Suga H (2012). Discovery of macrocyclic peptides armed with a mechanism-based warhead: isoform-selective inhibition of human deacetylase SIRT2. Angew. Chem..

[37] Rauh D (2013). An acetylome peptide microarray reveals specificities and deacetylation substrates for all human sirtuin isoforms. Nat. Commun..

[38] Yamagata K (2014). Structural basis for potent inhibition of SIRT2 deacetylase by a macrocyclic peptide inducing dynamic structural change. Structure.

[39] Fatkins DG, Monnot AD, Zheng W (2006). N^ε^-thioacetyl-lysine: a multi-facet functional probe for enzymatic protein lysine N^ε^-deacetylation. Bioorg. Med. Chem. Lett..

[40] Smith BC, Denu JM (2007). Mechanism-based inhibition of Sir2 deacetylases by thioacetyl-lysine peptide. Biochemistry.

[41] Schuster S (2016). A continuous sirtuin activity assay without any coupling to enzymatic or chemical reactions. Sci. Rep..

[42] Feldman JL, Dittenhafer-Reed KE, Denu JM (2012). Sirtuin catalysis and regulation. J. Biol. Chem..

[43] Rumpf T (2015). Selective SIRT2 inhibition by ligand-induced rearrangement of the active site. Nat. Commun..

[44] Wang L, Xie J, Schultz PG (2006). Expanding the genetic code. Annu. Rev. Biophys. Biomol. Struct..

[45] Dumas A, Lercher L, Spicer CD, Davis BG (2015). Designing logical codon reassignment—expanding the chemistry in biology. Chem. Sci..

[46] He B (2018). Compositional bias in naïve and chemically-modified phage-displayed libraries uncovered by paired-end deep sequencing. Sci. Rep..

[47] Tropea, J.E., Cherry, S. & Waugh, D.S. Expression and purification of soluble his6-tagged TEV protease. in *High Throughput Protein Expression and Purification:* Methods *and Protocols*. (ed. Doyle, S.A.) 297–307 (Humana Press, Totowa, NJ, 2009). 10.1007/978-1-59745-196-3_19 18988033

[48] Heinis C (2013). Phage selection of bicyclic peptides. Methods.

[49] Schrödinger Release 2017-4. (Maestro, Schrödinger, LLC, New York, NY, 2017).

[50] Bowers, K. J. et al. Scalable algorithms for molecular dynamics simulations on commodity clusters. *Proc. ACM/IEEE Conference on Supercomputing (SC06)*, Tampa, Florida, November 11–17 (2006).

[51] Schrödinger Release 2017-4: Desmond Molecular Dynamics System. (D. E. Shaw Research, New York, NY, 2017; Maestro-Desmond Interoperability Tools, Schrödinger, New York, NY, 2017).

[52] Sastry GM, Adzhigirey M, Day T, Annabhimoju R, Sherman W (2013). Protein and ligand preparation: parameters, protocols, and influence on virtual screening enrichments. J. Comput. Aid. Mol. Des..

[53] Schrödinger Release 2017-4: Schrödinger Suite 2017-4 Protein Preparation Wizard. (Epik Schrödinger, LLC, New York, NY, 2017; Impact, Schrödinger, LLC, New York, NY, 2016; Prime, Schrödinger, LLC, New York, NY, 2017).

[54] Jacobson MP (2004). A hierarchical approach to all-atom protein loop prediction. Proteins.

[55] Jacobson MP, Friesner RA, Xiang Z, Honig B (2002). On the role of crystal packing forces in determining protein side-chain conformations. J. Mol. Biol..

[56] Harder E (2016). OPLS3, a force field providing broad coverage of drug-like small molecules and proteins. J. Chem. Theory Comput..

[57] Jorgensen WL, Madura JD (1985). Temperature and size dependence of Monte Carlo simulations of TIP4P water. Mol. Phys..

[58] Zielkiewicz J (2005). Structural properties of water: comparison of the SPC, SPCE, TIP4P, and TIP5P models of water. J. Chem. Phys..

