# Supplementary material for: An amber obligate active site-directed ligand evolution technique for phage display
Source: Nat Commun. 2020 Mar 13;11:1392. doi: 10.1038/s41467-020-15057-7 (PMC7070036; doi:10.1038/s41467-020-15057-7)
Supplement: Supplementary file 6 — Supplementary Information [file 41467_2020_15057_MOESM6_ESM.pdf]

SUPPLEMENTARY NOTES, FIGURES, AND CODE

An Amber Obligate Active Site-Directed Ligand Evolution Technique for Phage Display

Tharp et al.

Contents:

Supplementary Notes

Next Generation Sequencing of the Amber Obligate Library and Data Analysis

Synthetic Methods

Supplementary Figures 1-32

Supplementary R Scripts 1-3

SUPPLEMENTARY NOTES

Next Generation Sequencing of the Amber Obligate Library and Data Analysis

PCR Conditions

Solution	Volume (μL)	Final Concentration
5x Phusion GC Buffer	10	1x
10 mM dNTP	1	0.2 mM
Sample DNA	1	0.71 ng/μL
Forward Primer	0.5	1 μM
Reverse Primer	0.5	1 μM
High Purity Water	36	
Phusion HiFidelity Polymerase	1	2 U

PCR Cycles

Temperature (°C)	Time (s)	Cycles
98	60	
98	10	30
55	10	
69	30	
72	30	
12	Hold	

Summary of R Scripts

Name	Description
Filters Reads with DADA Platform.R	1) Plots FastQC quality scores for forward and reverse reads<br>2) Reads are truncated accordingly and filtered using an expected error cutoff of 1
Amino Acid Analysis.R	1) Filtered fastq files are read into an R object for forward and reverse reads<br>2) Paired-End processing filters out any reads with more than one mismatch in the primer region or any mismatch in the library region

	3) Library regions are broken into codons and translated into a matrix containing peptide sequences.<br>4) Peptides in the matrix are counted and heatmaps showing library bias are generated
NNK Analysis.R	1) Sequences passing the paired-end processing are analyzed by nucleotide at each position<br>2) A table of nucleotide composition by position in library is returned.

Synthetic Methods

Synthesis of N^{ε}-butyryl-lysine (5) and N^{ε}-crotonyl-lysine (6)

N^{ε}-butyrlyl-lysine (**5**) and N^{ε}-crotonyl-lysine (**6**) were synthesized according to our previously reported procedure.[1]

Synthesis of N^{ε}-thiobutyryl-lysine (7) and N^{α}-Fmoc-N^{ε}-thiobutyrlyl-lysine (13)

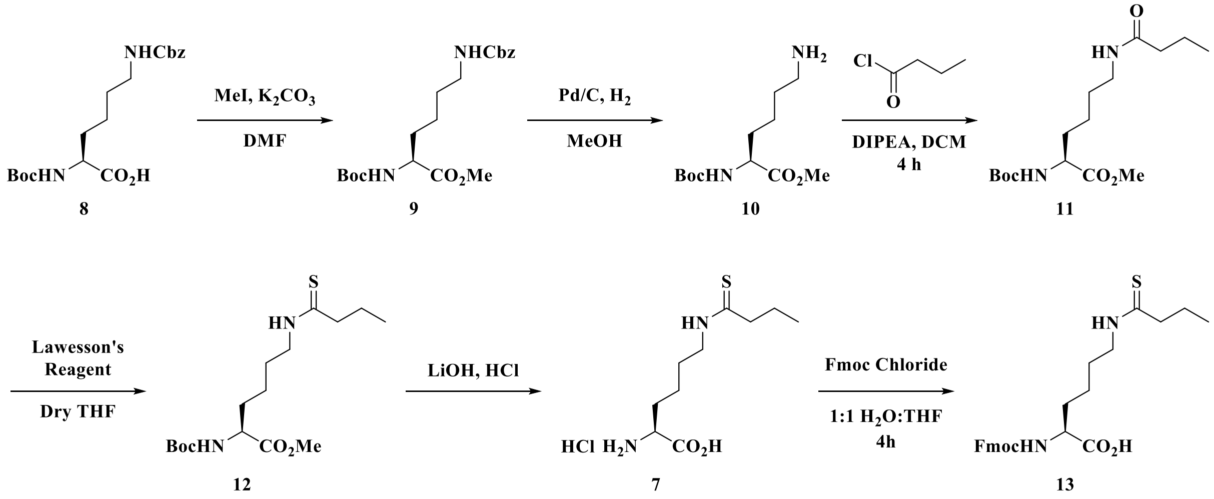

To a suspension of Boc-Lys(Z)-OH (**8**, 40.3 g, 0.106 mol) and potassium carbonate (27.6 g, 0.200 mol) in DMF (200 mL) was added iodomethane (9.90 mL, 0.159 mol), and the mixture was stirred at room temperature for 30 hours. The mixture was filtered, and the filter cake was washed with ethyl acetate (50 mL), dissolved in water (100 mL), and extracted with ethyl acetate (2x 100 mL). All of the ethyl acetate solutions were combined with the filtrate, and the solution was evaporated under vacuum until most of the DMF

had been removed. The residue was dissolved in ether (250 mL), washed with water (100 mL) and brine (50 mL), dried (Na_2SO_4), and evaporated to afford **9** (41.8 g, quant.) as a yellow oil. The material was pure enough for the next reaction without further treatment.

A solution of **9** (4.20 g, 10.6 mmol) in methanol (100 mL) was hydrogenated under an H_2 balloon in the presence of palladium on carbon (10 wt.% Pd, 0.71 g, 0.67 mmol) at room temperature for 3 hours, and TLC analysis showed complete conversion. The mixture was then filtered over a pad of Celite and the solution was directly used for the next reaction. The material should be immediately used without purification since prolonged storage at room temperature or flash chromatography would contribute to lactam formation.

To a solution of the above amine (**10**, ~9.15 mmol) in anhydrous dichloromethane (90 mL), cooled in an ice bath, was added *N,N*-diisopropylethylamine (2.80 mL, 16.07 mmol) dropwise, followed by a solution of n-butyryl chloride (1.10 mL, 11.07 mmol) in dichloromethane (10 mL) dropwise over 20 minutes. The mixture was then stirred at room temperature for 12 hours, and it was washed with sodium hydroxide solution (0.5 M, 20 mL) and brine (2x 20 mL), dried (Na_2SO_4), evaporated, and purified by column chromatography (EtOAc/hexanes, 1:3) to give **11** (2.77 g, 79% for two steps) as a colorless oil. Rf = 0.28 (EtOAc/hexanes, 1:2).

To a solution of compound **11** (2.5 g, 7.5 mmol) in THF (20 mL) was added Lawesson's reagent (3.0 g, 7.5 mmol) at room temperature. The reaction mixture was stirred overnight under nitrogen (monitored by TLC). After removal of THF using a rotary evaporator, the residue was purified by silica gel column (EtOAc/hexanes = 3:7) to give the product **12** as light yellow oil (1.96 g, 75% yield).

To a solution of **12** (1.90 g, 5.49 mmol) in THF (30 mL) was added lithiumhydroxide solution (1.0 M, 5.5 mL, 5.49 mmol), and the mixture was stirred at room temperature for 2 hours. The mixture was diluted in water (20 mL) and extracted with ether (2x 30 mL). The ether extracts were discarded, and the remaining aqueous solution was adjusted to pH 3 with hydrochloric acid (3 M), with the concomitant formation of white precipitate. The suspension was extracted with ethyl acetate (2x 30 mL), and the combined organic phases were washed once with brine (20 mL), dried (Na_2SO_4), and evaporated to give the crude carboxylic acid as a colorless oil, which was used without further purification.

The above crude acid (~4.8 mmol) was dissolved in 1,4-dioxane (10 mL), and hydrogen chloride in 1,4-dioxane (4.0 M, 2.4 mL, 9.63 mmol) was added. The resulting white suspension was stirred at room temperature for 20 hours, filtered, washed with dichloromethane, and dried to give **7** (1.03 g, 70% for two steps) as a white solid (^{1}H NMR (500 MHz, D_2O): δ 3.93 (t, *J* = 3.6 Hz, 1H), 3.48 (t, *J* = 3.6 Hz, 2H), 2.49 (t, *J* = 4.2 Hz, 2H), 1.92-1.79 (m, 2 H), 1.60-1.52 (m, 4 H), 1.38-1.30 (m, 2H), 0.73 (t, *J* = 4.2 Hz, 3H). ^{13}C NMR (125 MHz, D_2O) δ 204.8, 171.8, 52.6, 47.3, 45.3, 29.3, 26.3, 22.3, 21.6, 12.3).

Compound **7** (1.00 g, 3.73 mmol) and $NaHCO_3$ (1.17 g, 11.1 mmol) were dissolved in a mixture of 20 mL H_2O and 20 mL THF. The solution was cooled to 0 °C in an ice bath, and 9-fluorenylmethyl chloroformate (1.17 g, 4.47 mmol) dissolved in dry THF was added dropwise over 10 minutes. The reaction mixture was stirred for 4 hours. THF was evaporated and the aqueous phase was acidified with 3 M HCl (aq.) to pH 2-3. The product was extracted 3 times with EtOAc and the organic phase was dried and evaporated. The product was purified by column chromatography (MeOH gradient 0-10% in DCM) to give product **13** (1.00 g, 60%) as a solid. Proton and carbon NMR for **13** are shown below (^{1}H NMR (300 MHz, CD_3OD): δ 7.80 (d, *J* = 4.5 Hz, 2H), 7.69 (t, *J* = 4.8 Hz, 2H), 7.40 (t, *J* = 4.5 Hz, 2H), 7.32 (t, *J* = 4.8 Hz, 2H), 4.36 (d, *J* = 3.9Hz, 2H), 4.23 (t, *J* = 4.2 Hz, 1H), 4.16-4.13 (m, 1H), 3.60 (t, *J* = 3.9 Hz, 2H), 2.57 (t, *J* = 4.5 Hz, 2H), 1.90-1.85 (m, 1H), 1.78-1.65 (m, 5H), 1.47-1.44 (m, 2H), 0.93 (t, *J* = 4.5 Hz, 3H). ^{13}C NMR (125 MHz, CD_3OD); δ 204.7, 175.1, 157.2, 143.9, 143.7, 141.9, 127.4, 126.8, 124.8, 119.5, 66.8, 54.1, 45.2, 31.1, 26.9, 23.0, 22.6, 12.2).

Synthesis of TB (19)

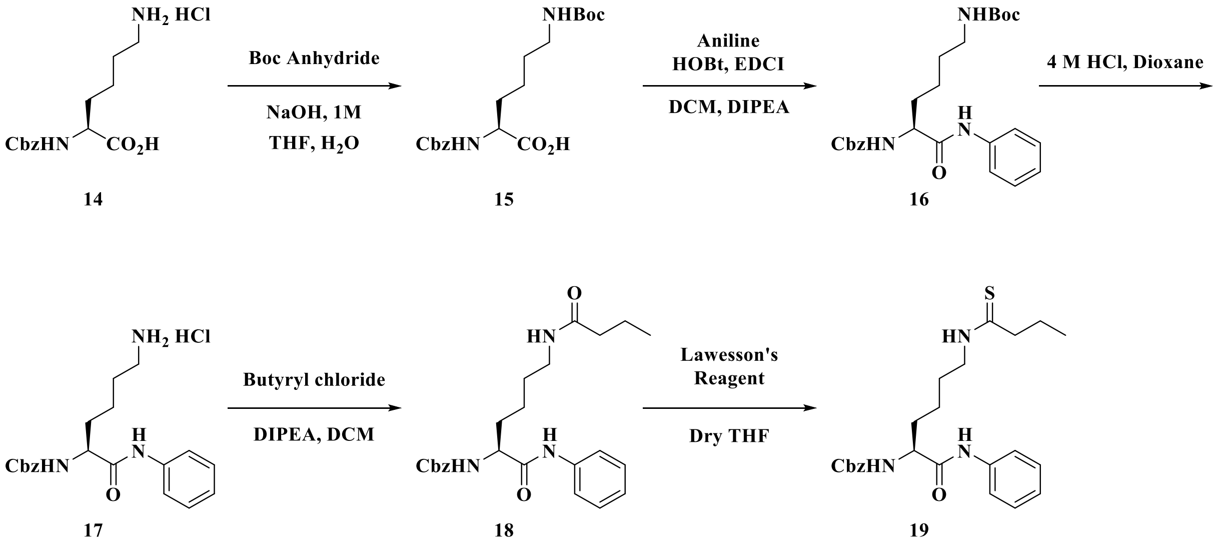

To a 500 mL of round-bottom-flask was added Z-Lys-OH (**14**, 5.0 g, 15.82 mmol), followed by THF (100 mL) and aqueous NaOH (1 M, 17.40 mL, 17.4 mmol). The reaction mixture was cooled to 0 ^{o}C. Di-*tert*-butyl dicarbonate (3.75g g, 17.4 mmol) was dissolved in THF (100 mL) and added to the above mixture dropwise over 40 minutes. Then the resulting mixture was stirred at room temperature for 20 hours. THF was removed under reduced pressure. The residue was diluted with water (100 mL), and extracted with EtOAc (100 mL). The aqueous layer was collected and acidified to pH 3 with HCl (1 M in water), then extracted with EtOAc (3x 100 mL). The combined organic layers were dried over anhydrous Na_2SO_4 and concentrated under reduced pressure to afford compound **15** (4.81 g, 80% yield). Compound **15** was pure enough for the next step without further purification.

A mixture of **15** (2.20 g, 5.5 mmol), aniline (1.15 g, 11.0 mmol), EDCI (1.63 g, 8.5 mmol) and HOBt·H_2O (1.34 g, 8.5 mmol) in DCM (100 mL) was stirred for 24 hours at room temperature. The reaction mixture was poured into water and the whole was extracted with EtOAc. The EtOAc layer was separated, washed with saturated aqueous $NaHCO_3$, brine, and dried over Na_2SO_4. Filtration and concentration *in vacuo* gave a colorless solid. The solid was suspended in n-hexane and collected by filtration to give 2.53 g (90%) of **16** as a colorless solid.

To a solution of the fully protected compound **16** (2.52 g, 5.53 mmol) dissolved in 15 mL dioxane was added HCl (2.76 mL, 11.07 mmol, 4 M in dioxane) and the mixture was

stirred and monitored by TLC for 8 hours. After complete removal of the solvent, compound **17** was obtained as a white powder (2 g, 92%). **17** was pure enough to use for the next step without further purification.

To a solution of the above amine (**17**, 2 g, 5.10 mmol), in anhydrous dichloromethane (30 mL), cooled in an ice bath, was added *N,N*-diisopropylethylamine (1.10 mL, 7.92 mmol) dropwise, followed by a solution of n-butyryl chloride (1.10 mL, 7.91 mmol) in dichloromethane (10 mL) dropwise over 5 minutes. The mixture was then stirred at room temperature for 12 hours, and it was washed with sodium hydroxide solution (0.5 M, 20 mL) and brine (2x 20 mL), dried (Na_2SO_4), evaporated, and flash chromatographed (EtOAc/hexanes, 1:3) to give **18** (1.85 g, 85%) as a colorless oil. Rf = 0.28 (EtOAc/hexanes, 1:2).

To a solution of compound **18** (1.5 g, 3.52 mmol) in THF (20 mL) was added Lawesson's reagent (1.42 g, 3.52 mmol) at room temperature. The reaction mixture was stirred overnight under nitrogen (monitored by TLC). After removal of THF using a rotary evaporator, the residue was purified by silica gel column (EtOAc/Hexane = 3:7) to give the product **19** as light yellow oil (1.1 g, 75% yield). Proton and carbon NMR of **19** are shown below (^{1}H NMR (300 MHz, $CDCl_3$): δ 8.43 (br, 1H), 7.70 (br, 1H), 7.51 (d, *J* = 4.5 Hz, 2H), 7.34-7.28 (m, 7H), 7.12-7.11(m, 1H), 5.72 (d, *J* = 4.8 Hz, 2H), 5.15-5.09 (m, 2H), 4.38 (br, s, 1H), 3.70-3.60 (m, 2H), 2.59 (t, *J* = 4.8 Hz, 2H), 1.97-1.90 (m, 1H), 1.83-1.72 (m, 6H), 1.49-1.48 (m, 2H), 0.93 (t, *J* = 4.5 Hz, 3H). ^{13}CNMR (125 MHz, $CDCl_3$); δ 205.4, 170.7, 156.8, 137.4, 135.9, 128.9, 128.5, 128.2, 127.8, 124.7, 120.3, 6.2, 60.5, 55.3, 48.6, 45.4, 32.2, 27.1, 22.8, 22.7, 21.1, 14.2, 13.9).

Synthesis of N^{α}-Fmoc-N^{ε}-thiomyristyl-lysine (N^{α}-Fmoc-tMyK)

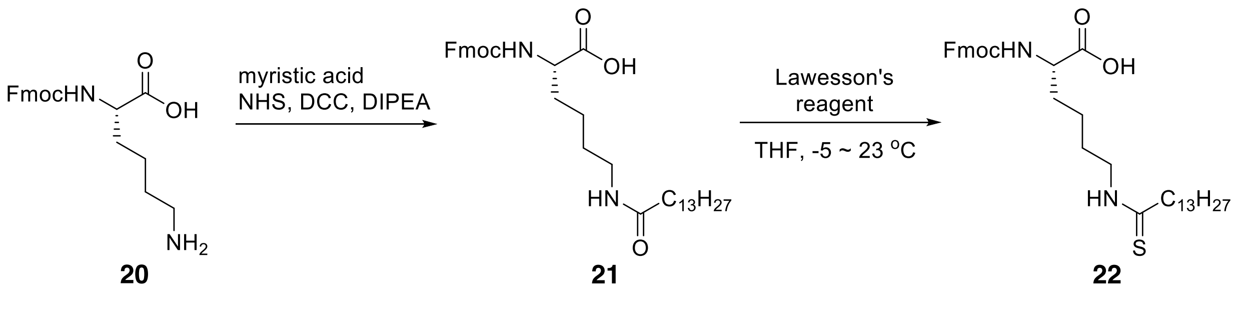

To a solution of myristic acid (2.28 g, 10 mmol) in anhydrous DMF (20 mL), *N*-hydroxysuccinimide (1.15 g, 10 mmol) was added *N*,*N'*-dicyclohexylcarbodiimide solution (DCC, 2.06 g, 10 mmol dissolved in anhydrous 10 mL DMF) at room temperature. After stirring for 2 hrs, the reaction mixture was filtered. The filtrate was added to a solution of Fmoc-Lys-OH (3.68 g, 10 mmol) with *N*,*N*-diisopropylethylamine (DIPEA, 1.74 mL, 10 mmol) in anhydrous DMF (10 mL) at room temperature. The resulting reaction mixture was stirred overnight. Then the reaction mixture was poured into water and neutralized with 1 M HCl to adjust pH to 2~3. The precipitate was collected by filtration and dried to give the crude product. Then the crude compound was purified by silica gel column chromatography (silica gel, CH_2Cl_2:CH_3OH = 40:1) to give compound **21** as a white solid (4.74 g, 82% yield). ^{1}H NMR (400 MHz, CD_3OD): δ 7.80 (d, 2H, J = 8.0 Hz), 7.68 (t, 2H, J = 8.0 Hz), 7.35 (dt, 4H, J = 32.0 and 8.0 Hz), 4.38-4.31 (m, 2H), 4.23 (d, 1H, J = 8.0 Hz), 4.17-4.12 (m, 1H), 3.18 (t, 2H, J = 8.0 Hz), 2.15 (t, 2H, J = 8.0 Hz), 1.92-1.80 (m, 1H), 1.76-1.65 (m, 1H), 1.62-1.38 (m, 6H), 1.35-1.19 (m, 20H), 0.89 (t, 3H, J =8.0 Hz). ESI calcd. for $C_{35}H_{51}N_2O_5$ ($[M+H]^+$) 579.4, obsd. 579.4.

To a solution of **21** (1.50 g, 2.59 mmol) in THF (50 mL) was cooled to -5 °C in ice-salt bath under nitrogen. Lawesson's reagent (629 mg, 1.56 mmol) was added portionwise to the reaction solution at -5 °C. The resulting mixture was stirred at -5 °C, and gradually increased to room temperature for 12 hrs. The reaction mixture was cooled to -5 °C, Lawesson's reagent (190 mg, 0.47 mmol) was added. The resulting mixture was stirred at -5 °C to room temperature for 8 hrs. After removing THF using a rotary evaporator, the residue was purified using silica gel column chromatography (silica gel, CH_2Cl_2:CH_3OH = 50:1) to give compound **22** as an off-white solid (480 mg, 31.2% yield). ^{1}H NMR (400 MHz, CD_3OD): δ 7.81 (d, 2H, J = 8.0 Hz), 7.71 (t, 2H, J =8.0 Hz), 7.36 (dt, 4H, J = 28 and 8.0 Hz), 4.48-4.37 (m, 2H), 4.27-4.18 (m, 2H), 3.63-3.58 (m, 2H), 2.59 (t, 2H, J =8.0), 1.96-1.87 (m, 1H), 1.78-1.60 (m, 5H), 1.54-1.38 (m, 2H), 1.34-1.20 (m, 20H), 0.86 (t, 3H, J = 8.0 Hz). MS (ESI) calcd. for $C_{35}H_{51}N_2O_4S$ $[M+H]^+$ 595.4, obsd. 595.4.

Solid Phase Peptide Synthesis of S2P03-FITC that has BuK at the X position

FITC-conjugated peptides S2P03-BuK and S2P03-K were synthesized on a ProTide rink amide resin (CEM #R003) using an automated Liberty Blue Peptide Synthesizer with an HT12 attachment. Fmoc-amino acids were deprotected using 20% piperidine in DMF with a two-step microwave cycle: 1) 75 °C, 15 s 2) 90 °C, 50 s. Residues were then coupled with 0.125 M DIC and 0.25 M Oxyma using CEM's standard microwave coupling cycle: 75 °C, 15 s 2) 90 °C, 110 s. To the N-terminus was linked 6-(Fmoc-amino)caproic acid (Matrix Scientific), coupled twice. The final Fmoc group was deprotected, and a final coupling with FITC (0.08 M with 0.16 M diisopropylethylamine, 4 and 2 equivalents respectively) was performed. The peptides were cleaved from the resin by agitating for 2 hours in 5 mL of 92.5:2.5:2.5:2.5 TFA:H_2O:DODT:TIS. The products were then filtered and peptides were precipitated out of the filtrate using 40 mL of cold ether. The precipitates were collected by centrifugation and washed again with cold ether. The peptides were then dissolved in 1 mL of DMF and purified by reverse-phase semi-preparative HPLC (30-50% Acetonitrile in Water 1% formic acid, 20 min, 4 mL/min, PDA) using a Discovery BIO wide pore C18-5 column (25 cm x 10 mm, Millipore-Sigma #568230-U).

Solid Phase Peptide Synthesis of S2P03, S2P04, S2P04-5, Ac-S2P04-5, and S2P07 that have tBuK at the X position

The peptides S2P03-tBuK, S2P04-tBuK, S2P04-5-tBuK, Ac-S2P04-5-tBuK, and S2P07-tBuK were synthesized via manual Fmoc-based solid-phase peptide synthesis on Rink-amide resin using *N*,*N*,*N'*,*N'*-tetramethyl-O-(1*H*-benzotriazol-1-yl)uranium hexafluorophosphate (HBTU) as a coupling agent. Rink-amide MBHA resin (Novabiochem, 100 mg, substitution: 0.78 mmol g^{-1}) was swelled for 1 h in 10 mL of DMF. Deprotection, coupling, and cleavage reactions were carried out at room temperature in a glass reaction vessel agitated with end-over-end rotation under the following conditions:

Deprotection: A solution of 20% Piperidine in DMF (v:v, 5 mL) was added to the resin for 5 min. The resin was washed (3x 5 mL DMF) and the deprotection reaction was repeated for 15 min. After the second deprotection reaction the resin was washed again (5x 5 mL DMF, 2x 5 mL DCM).

Coupling: Each coupling reaction contained the Fmoc-protected amino acid (312 µmol, 4 eq.), HBTU (304.2 µmol, 3.9 eq.), and *N,N*-diisopropylethylamine (DIPEA, 780 µmol, 10 eq.) dissolved in 5 mL DMF. The Fmoc-protected amino acid was preactivated with HBTU and DIPEA for 5 min before adding to the resin for 40 min. After 40 min the resin was washed (5x 5 mL DMF, 2x 5 mL DCM).

N-Terminal Acetylation: For synthesizing Ac-S2P04-5 with an N-terminal acetylation, the resin was washed (5x 5 mL DMF, 3x 5 mL DCM) following the last Fmoc deprotection and then incubated with a solution of acetic anhydride, pyridine, and DMF (1:2:3, v:v:v) for 1 hour at room temperature with agitation. The peptide was then cleaved and simultaneously sidechain deprotected as described.

Cleavage: After the final Fmoc deprotection, the resin was washed (5x 5 mL DMF, 5x 5 mL DCM) and the peptide was cleaved from the resin and simultaneously sidechain deprotected using 7 mL of a cleavage cocktail containing 88% trifluoroacetic acid (TFA), 5% phenol, 5% H_2O, and 2% triisopropylsilane (v:w:v:v) for two hours under a blanket of nitrogen. After two hours, the resin was filtered and washed (2x 1 mL cleavage cocktail) and the combined filtrate was reduced to ~4 mL using a stream of nitrogen. The concentrated solution was added dropwise to cold diethyl ether (40 mL) and incubated on ice for 10 min. The precipitated peptide was collected by centrifugation (6k rpm, 10 min), washed twice with cold diethyl ether (20 mL), dried under a stream of nitrogen, and stored at -20 °C.

Purification: Crude peptides were dissolved in DMF and purified by semipreparative RP-HPLC on a C18 column (Alltech, 10 x 250 mm, 10 µm, 100 Å pore or Supelco, 10 x 250 mm, 5 µm, 300 Å pore). Solvent A: water with 0.1% TFA, Solvent B: acetonitrile with 0.1% TFA. The peptides were eluted with a linear gradient from 20-50% Solvent B over 30 min. Fractions containing the purified peptides were combined and lyophilized to afford a fluffy white powder. Masses of the peptides were confirmed by MALDI-TOF mass spectrometry. Peptide purity was determined according to the HPLC traces at 215 nm and was greater than 90% for all peptides used. MALDI-TOF mass spectra and HPLC traces for the purified peptides are shown in **Supplementary Fig. 16-21** and their detected and theoretical molecular weights are provided in **Supplementary Table 1**.

Solid Phase Peptide Synthesis of S2P03, S2P04, and S2P04(Abu)-5 that have tMyK at the X position

Peptides S2P03-tMyK, S2P04-tMyK, and S2P04(Abu)-5-tMyK were synthesized on a low loading ProTide rink amide resin (CEM #R002) using an automated Liberty Blue Peptide Synthesizer with an HT12 attachment. Fmoc-amino acids were deprotected using 20% piperidine in DMF with a two-step microwave cycle: 1) 75 °C, 15 s 2) 90 °C, 50 s. Residues were then coupled with 0.125 M DIC and 0.25 M Oxyma using CEM's standard microwave coupling cycle: 75 °C, 15 s 2) 90 °C, 110 s. The peptides were cleaved from the resin by agitating for 2 hours in 5 mL of 94:2.5:2.5:1 TFA:H_2O:DODT:TIS. The products were then filtered and peptides were precipitated out of the filtrate using 40 mL of cold ether. The precipitates were collected by centrifugation and washed again with cold ether. The peptides were then dissolved in 1 mL of DMF and purified by reverse-phase semi-preparative HPLC (40-70% Acetonitrile in Water 1% formic acid, 20 min, 4 mL/min, PDA) using a Discovery BIO wide pore C18-5 column (25 cm x 10 mm, Millipore-Sigma #568230-U).

SUPPLEMENTARY TABLES

Supplementary Table 1 | Molecular Masses of Synthetic Peptides.

Peptide	Expected Mass, Da			Observed Mass, Da
-	$[M+H]^+$	$[M+Na]^+$	$[M+K]^+$	-
S2P03-BuK	1322.6	1344.6	1360.7	1322.7, 662.0 (2+)
S2P03-K	1252.5	1274.5	1290.6	627.1 (2+)
S2P03-tBuK	836.4	858.4	874.5	858.8, 874.8
S2P04-tBuK	806.4	828.4	844.5	808.0, 829.3, 845.3
S2P07-tBuK	880.4	902.4	918.5	902.9, 918.9
S2P04-5-tBuK	648.4	670.3	686.4	670.5, 686.5
Ac-S2P04-5-tBuK	690.4	712.3	728.5	712.4, 728.4
S2P04(Abu)-5-tBuK	630.4	652.4	668.5	653.1, 668.0
S2P03-tMyK	976.6	998.6	1014.7	976.590, 488.799 (2+)
S2P04-tMyK	946.6	968.6	984.7	946.579, 473.794 (2+)
S2P04(Abu)-5-tMyK	770.6	792.6	798.7	770.555, 385.781 (2+)

Supplementary Table 2 | The IC_{50} values of S2P04, S2P03-tMyK and TB measured for SIRT1-3. Values are given as the mean ± standard deviation of two independent experiments. The IC_{50} value of S2P03-tMyK for SIRT3 was not determined.

**Compound**	**IC_{50}, µM**		
**-**	SIRT1	SIRT2	SIRT3
TB	9.62 ± 0.09	3.11 ± 0.04	>150
S2P04	0.25 ± 0.05	0.092 ± 0.003	14 ± 1
S2P03-tMyK	0.4 ± 0.1	0.010 ± 0.003	n.d.

SUPPLEMENTARY FIGURES

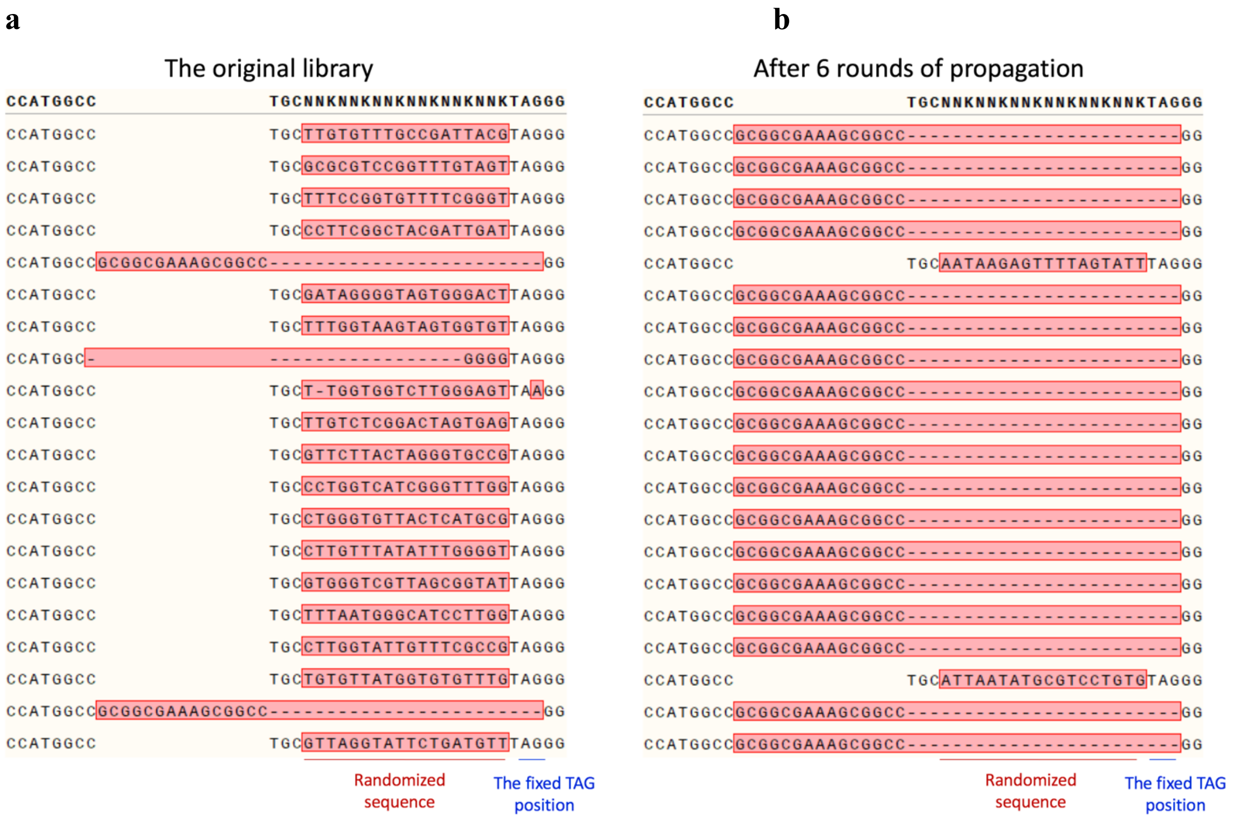

Supplementary Fig. 1 | Sequences of an amber obligate library that was generated by primer pairs that fixed an amber codon at the 7th coding position and randomized the other six coding sites before and after 6 rounds of propagation. The original phagemid turned to be dominate after 6 rounds of propagation.

a

5’ ***3’***

*...GCCGAGGATGAGTTG**TAG**ATTTGTGCG...*

...GCCTTGAGGTTGGTTGTTAGGGTTGCG...

...GCC - GCG...

...GCCTTG - ATTAGTATCTTTCGTATGCG...

b

5’ ***3’***

...GCCTTTTATACTCATGGGGTGCCTGCG...

...GCCACTACTTATGTGAAGTCTTCTGCG...

*...GCCTTTGTGCTGGTGAGT**TAG**GCTGCG...*

...GCCTGTAATTTTTTTTATTATTATGCG...

Supplementary Fig. 2 | The DNA sequence of phage clones during construction of the amber-codon-obligate library. a) The sequences of phages after the first round of superinfection-selection. Dashes represent base deletions. **b)** The sequence of phages after passage through *E. coli* DH5α.

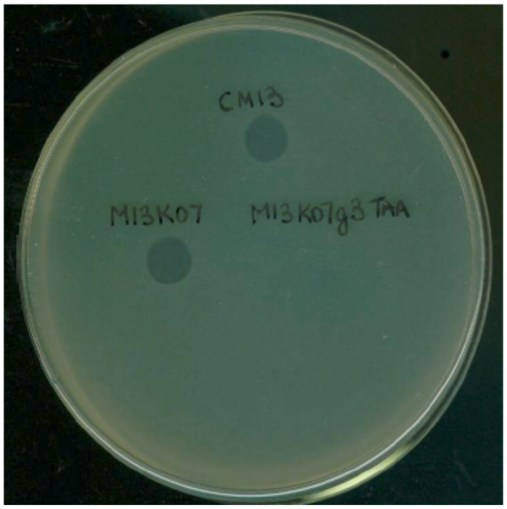

Supplementary Fig. 3 | Confirmation of pIII knockout in M13KO7(pIII⁻). Supernatants from *E. coli* cultures expressing M13KO7(pIII⁻) or wildtype M13KO7 and CM13 were spotted onto an overlay of top agar containing *E. coli* Top10 F'. The presence of plaques in the wildtype M13KO7 and CM13 spots indicates the presence of viable phages. The lack of plaques in the M13KO7(pIII⁻) spot confirms the loss of pIII which is required for host infection.

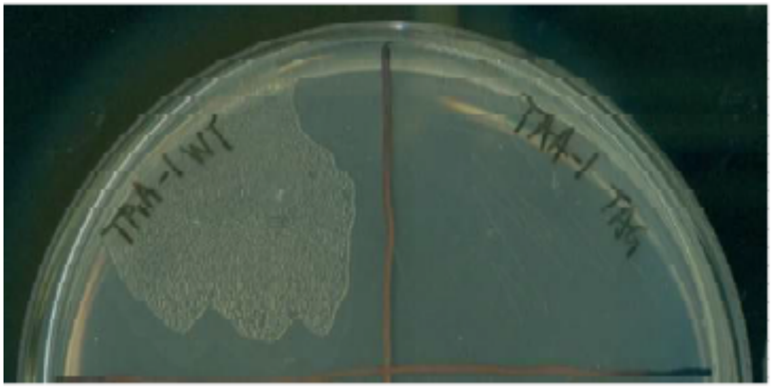

Supplementary Fig. 4 | M13KO7(pIII^{-}) phagemid complementation assay. *E. coli* Top10 F’ were infected with supernatants from cultures expressing wildtype pIII (left) or pIII with an in-frame amber mutation (right). The growth of cells infected with the wildtype pIII supernatant confirms the ability of M13KO7(pIII^{-}) to complement phagemids bearing viable pIII.

5′ **3′**

...T C G T T G **T A G** T T G C A G C A T T C T ...

...**T A G** A G T G C T A T T A C G T A T T G T ...

...A T T **T A G** A T T C A T C C G T A T T T T ...

...T A T A G T A T T A A G T G G **T A G** G T T ...

...G G T G C G **T A G** A G G C C G G G G A T G...

...C G T A T G C A T **T A G** G C G T T T T T T ...

...A C T A C T T A T A C T G G T G A T **T A G**...

...G G T T C G T T G C A T G T T C A T **T A G**...

...**T A G** T T G C A T C C G G G G A T G C G T ...

...T A T **T A G** A C T G A T T C T G A T G C T ...

Supplementary Fig. 5 | Sequence analysis after the second round of superinfection immunity-based selection confirmed the presence of an in-frame amber codon in all clones.

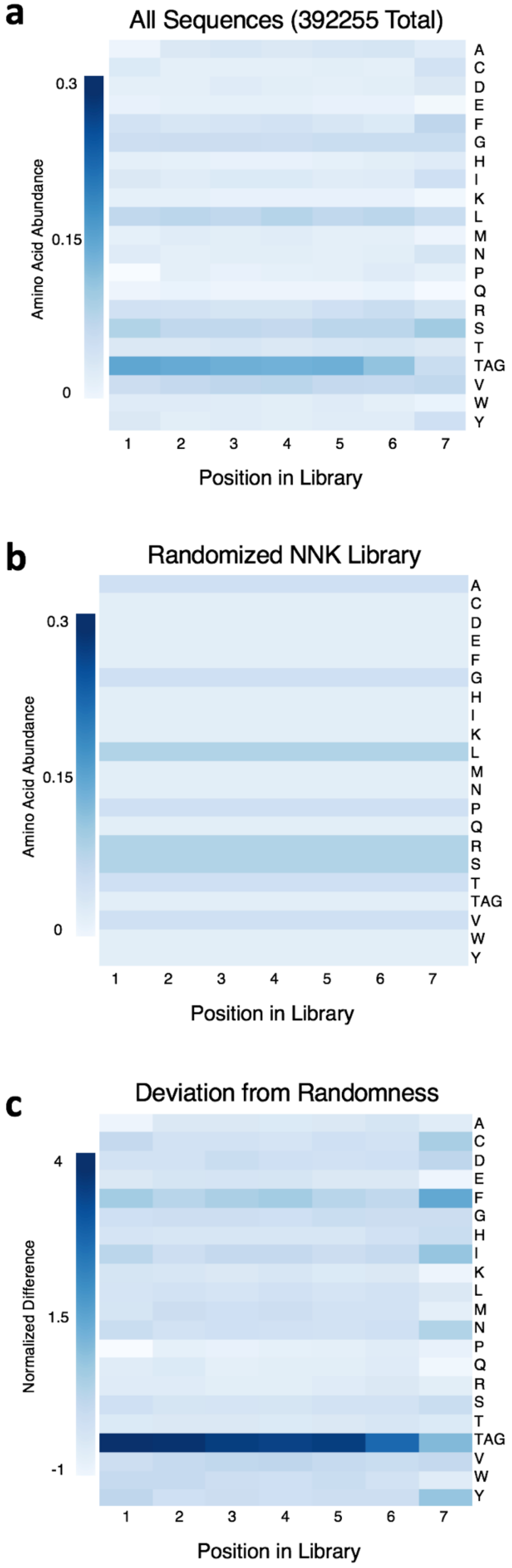
a
All Sequences (392255 Total)
Amino Acid Abundance
0.3
0.15
0
A
C
D
E
F
G
H
I
K
L
M
N
P
Q
R
S
T
TAG
V
W
Y
1 2 3 4 5 6 7
Position in Library
b
Randomized NNK Library
Amino Acid Abundance
0.3
0.15
0
A
C
D
E
F
G
H
I
K
L
M
N
P
Q
R
S
T
TAG
V
W
Y
1 2 3 4 5 6 7
Position in Library
c
Deviation from Randomness
Normalized Difference
4
1.5
-1
A
C
D
E
F
G
H
I
K
L
M
N
P
Q
R
S
T
TAG
V
W
Y
1 2 3 4 5 6 7
Position in Library

Supplementary Fig. 6 | Heat plots of amino acid occurrence in the amber obligate phage library generated using the superinfection immunity-based selection. a. The experimental data; b. Theoretical occurrence based on the calculation of randomness of nucleotides in the primers; c. Deviation of the experimental data from the theoretical calculation.

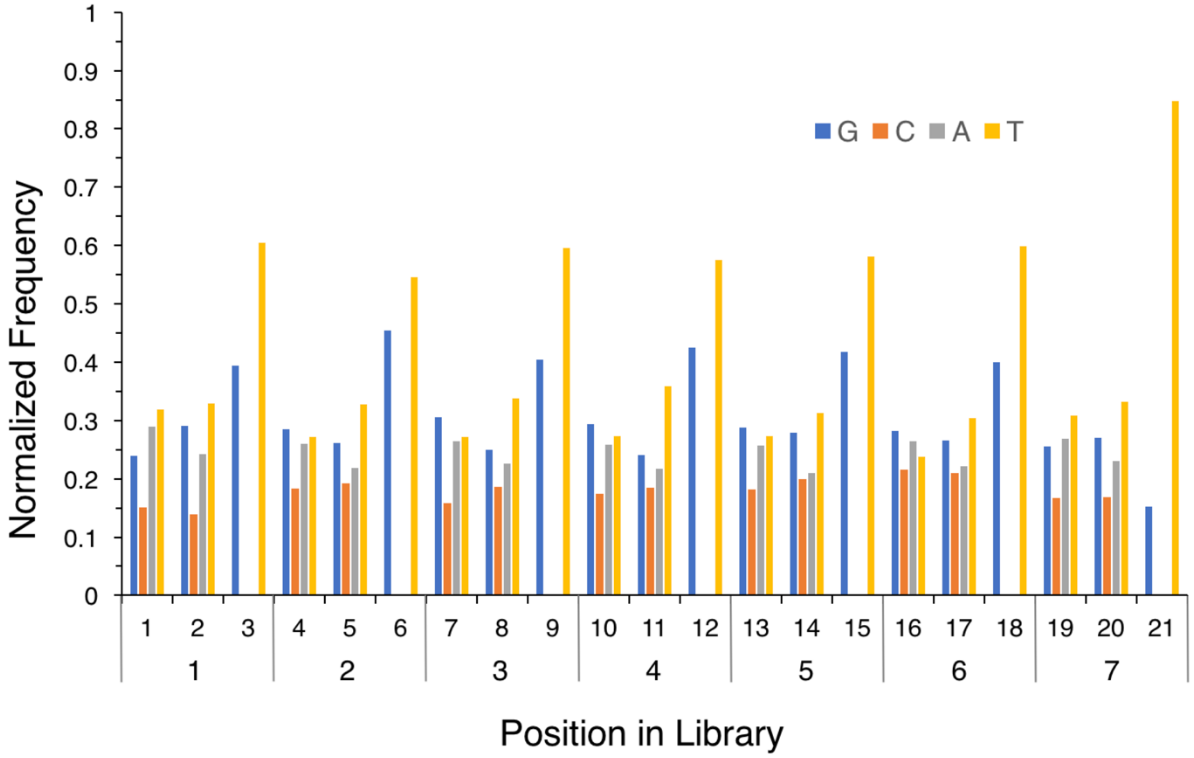

Supplementary Fig. 7 | The distribution of each nucleotide in all positions in the amber obligate phage library generated using the superinfection immunity-based selection.

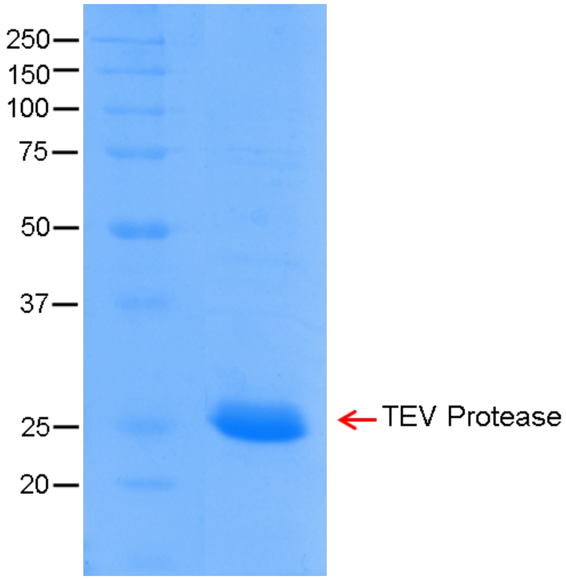

Supplementary Fig. 8 | SDS-PAGE analysis of the purified TEV protease. The TEV protease expression has been repeated once successfully. The reported molecular weights are in kDa units.

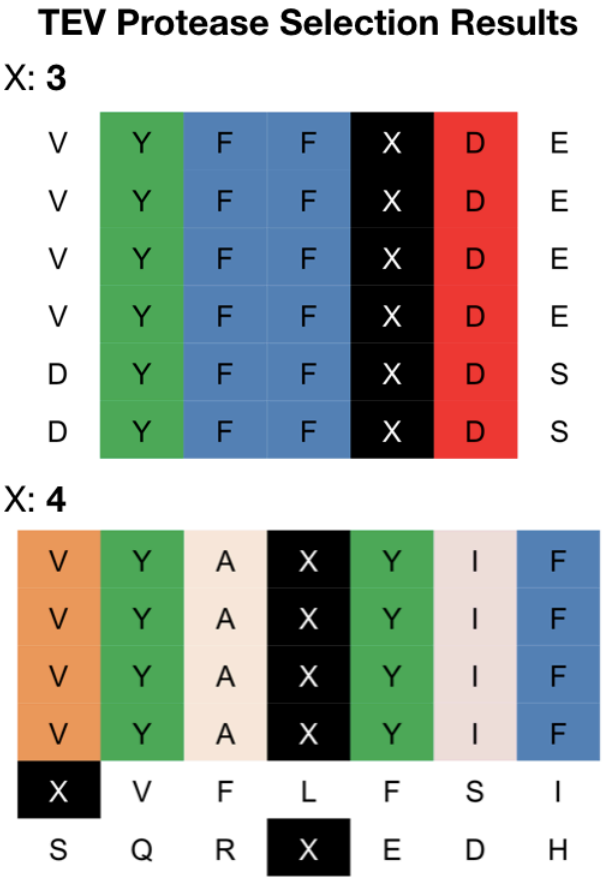

Supplementary Fig. 9 | The amino acid sequence of peptides isolated after the fourth round of selection against TEV protease.

Clone	1	2	3	4	5	6	7
	Third Selection Round						
SAP01	K	X	N	F	G	Y	Y
SAP02	K	W	K	V	X	T	S
SAP03	T	W	X	K	S	N	W
SAP04	M	A	K	P	Q	R	X
SAP05	R	E	H	K	P	X	N
SAP06	K	L	X	K	H	Y	P
	Fourth Selection Round						
SAP09	K	L	X	K	H	Y	P
SAP10	K	L	X	K	H	Y	P
SAP11	K	L	X	K	H	Y	P
SAP12	K	L	X	K	H	Y	P
SAP13	K	H	X	K	A	I	P
SAP14	K	M	X	P	Q	R	N
SAP15	K	M	X	P	Q	R	N
SAP16	K	M	X	P	Q	R	N
SAP17	K	M	X	P	Q	R	N
SAP18	K	M	X	P	Q	R	N
SAP19	K	W	C	X	N	C	R
SAP20	S	A	A	K	T	X	I

Supplementary Fig. 10 | The amino acid sequence of peptides isolated after the third and fourth round of selection against streptavidin.

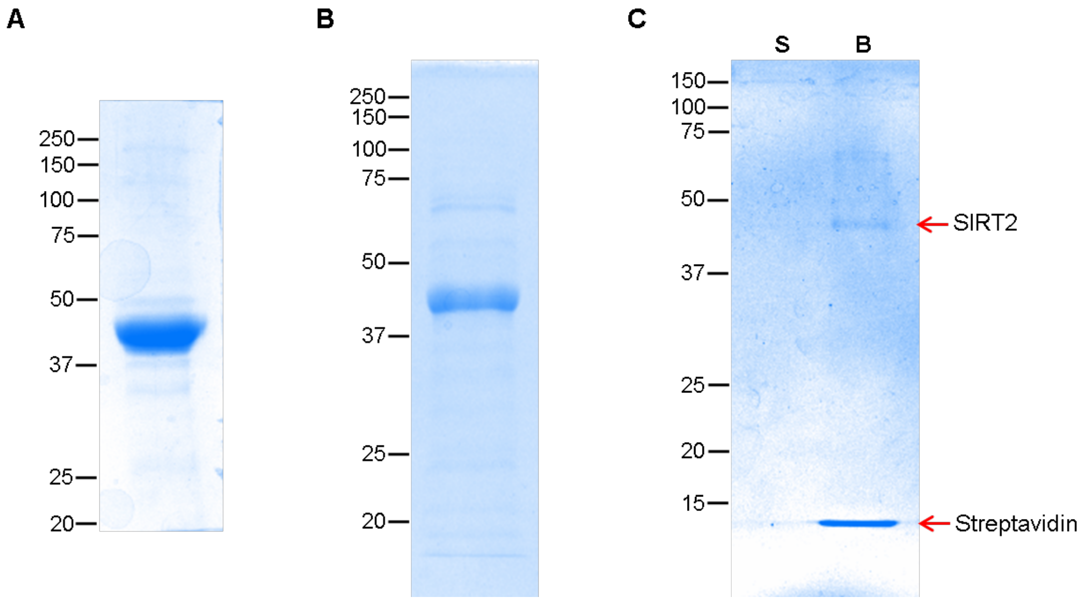

Supplementary Fig. 11 | SDS-PAGE analysis of purified SIRT2. (A) SIRT2 purified for activity assays; **(B)** Biotinylated SIRT2 purified for phage panning; **(C)** Pulldown assay to confirm biotinylation of SIRT2. The lane labelled “S” denotes supernatant whereas “B” denotes beads. SIRT2 expression and purification has been repeated successfully four times. All listed molecular weights are in kDa units.

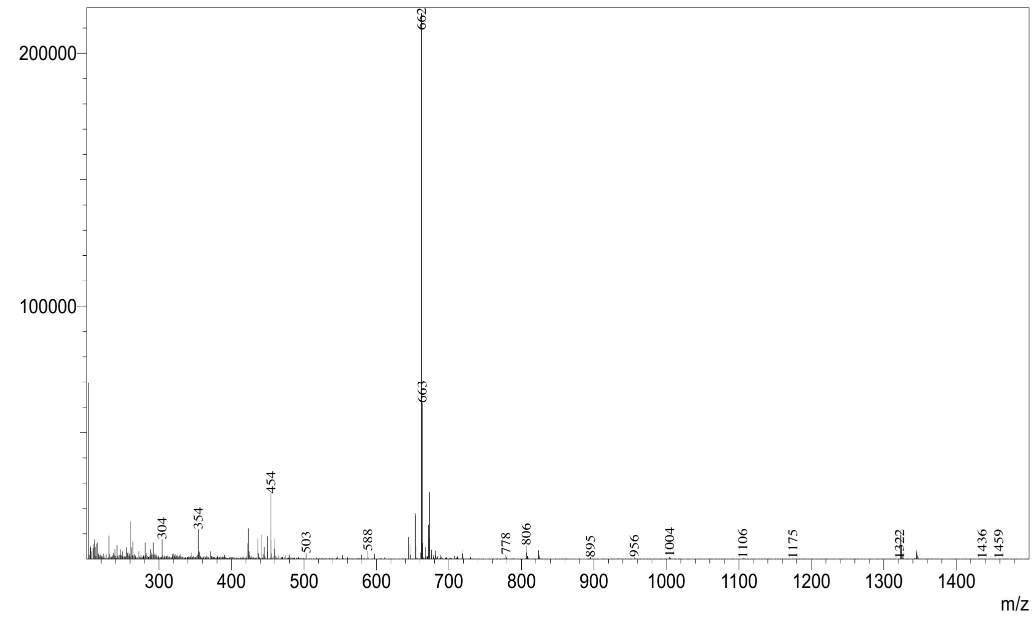

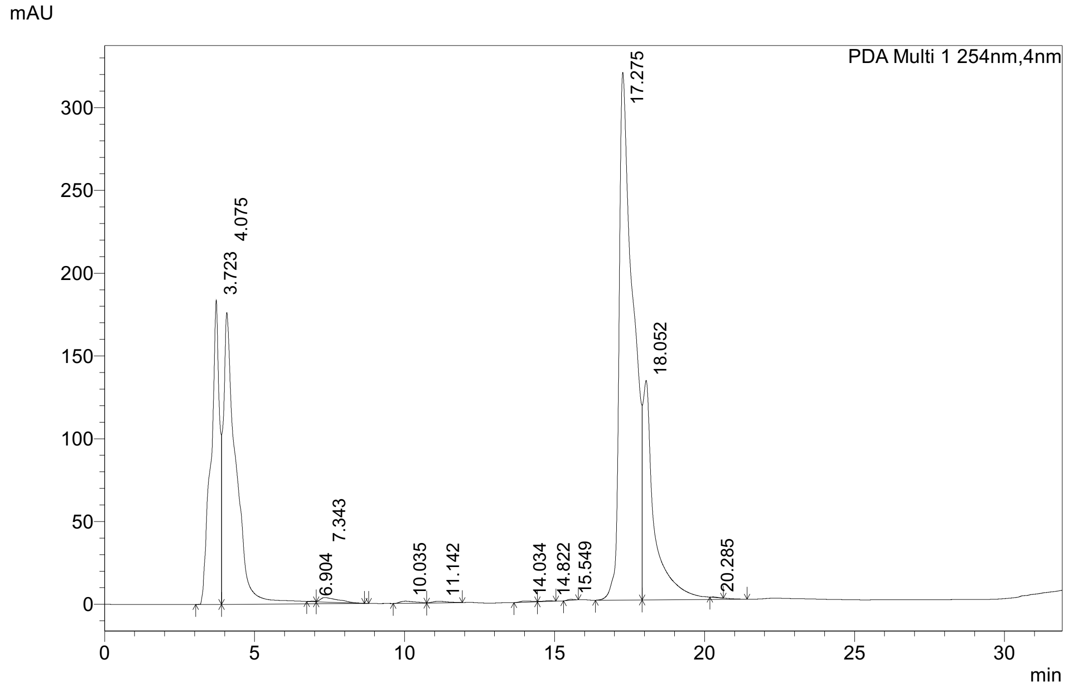

Supplementary Fig. 12 | Characterization data for S2P03-BuK. The peptide was eluted at 17.3 minutes using a 30-50% ACN/Water gradient over 20 minutes (from 5-25 min. on chromatogram). The peak from 3.7-4.2 minutes corresponds to DMF.

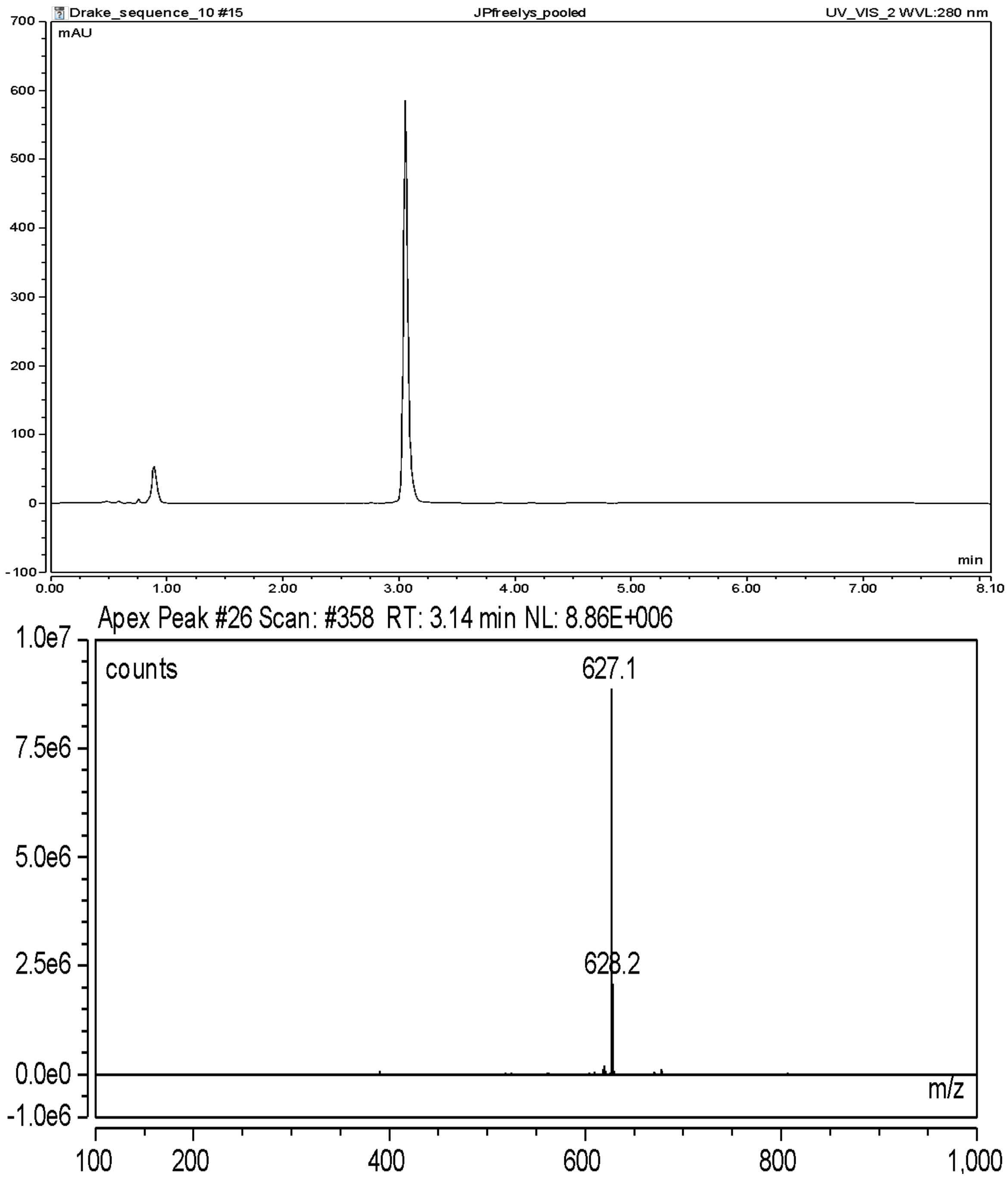

Supplementary Fig. 13 | Characterization data for S2P03-K. The peptide was eluted at 3 minutes using a 20-80% ACN/Water gradient in an analytical C18 column over 10 minutes.

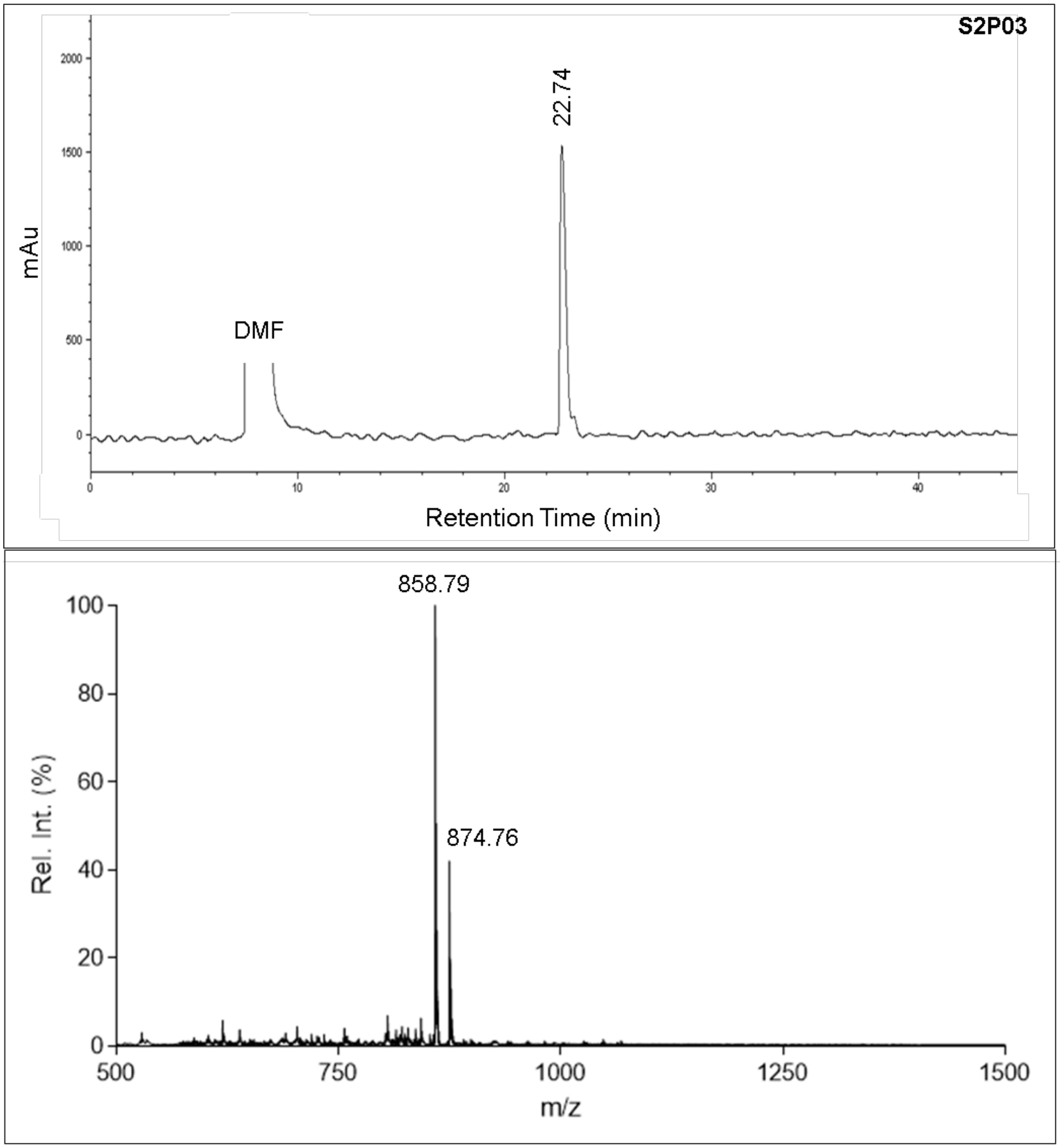

Supplementary Fig. 14 | Characterization data for the peptide S2P03-tBuK. (**Top**) HPLC trace at 215 nm. The peptide was eluted with a linear gradient from 20-50% acetonitrile in water (0.1% TFA) from 10-40 min. (**Bottom**) MALDI-TOF mass spectra. Calculated mass: $[M+H]^+$ = 836.44 Da, $[M+Na]^+$ =858.41 Da, $[M+K]^+$ = 874.52 Da; Observed Mass: 858.79 Da, 874.76.

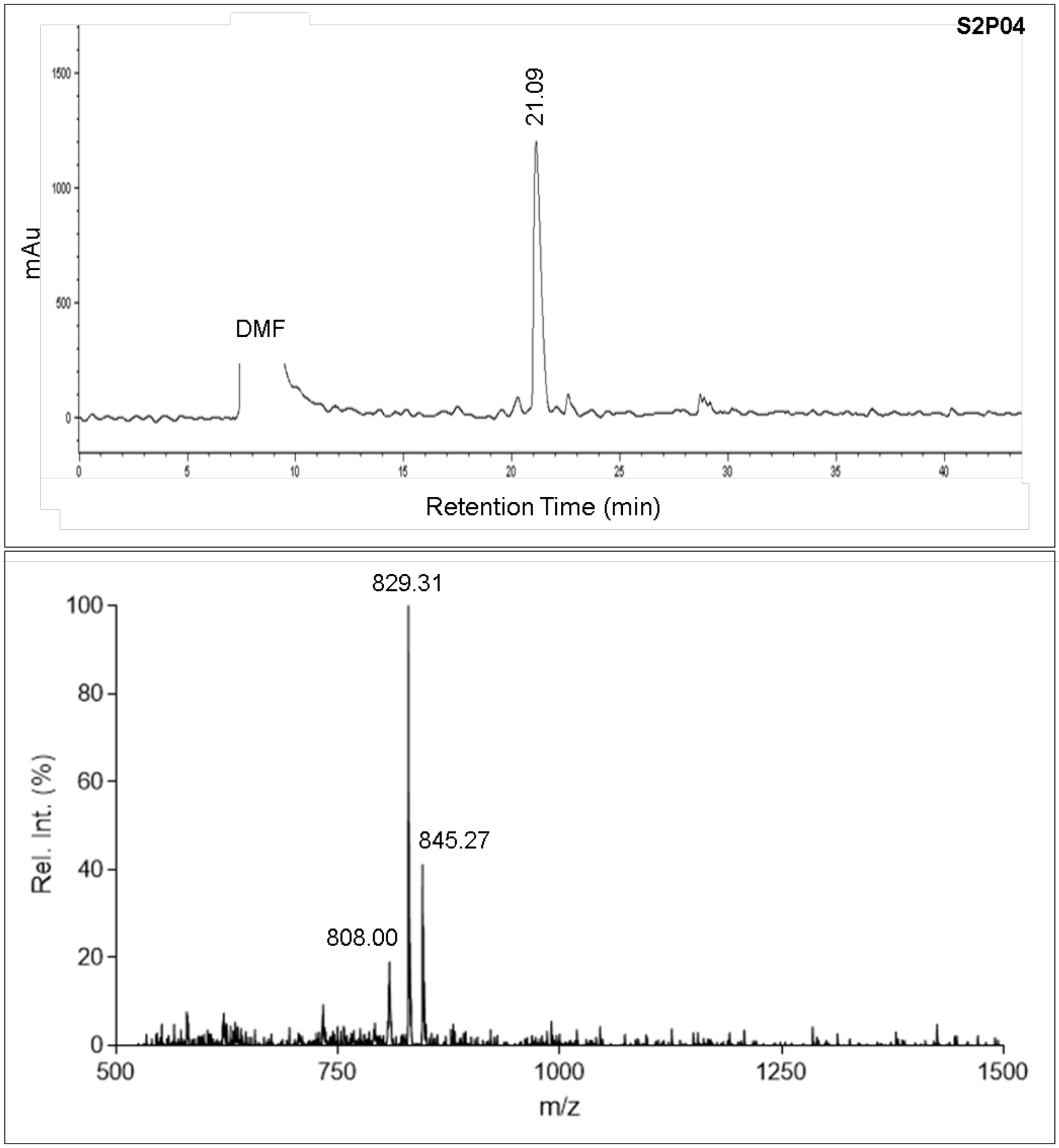

Supplementary Fig. 15 | Characterization data for the peptide S2P04-tBuK. (**Top**) HPLC trace at 215 nm. The peptide was eluted with a linear gradient from 20-50% acetonitrile in water (0.1% TFA) from 10-40 min. (**Bottom**) MALDI-TOF mass spectra. Calculated mass: $[M+H]^+$ = 806.43 Da, $[M+Na]^+$ = 828.41 Da, $[M+K]^+$ = 844.52 Da; Observed Mass: 808.00 Da, 829.31 Da, 845.27 Da.

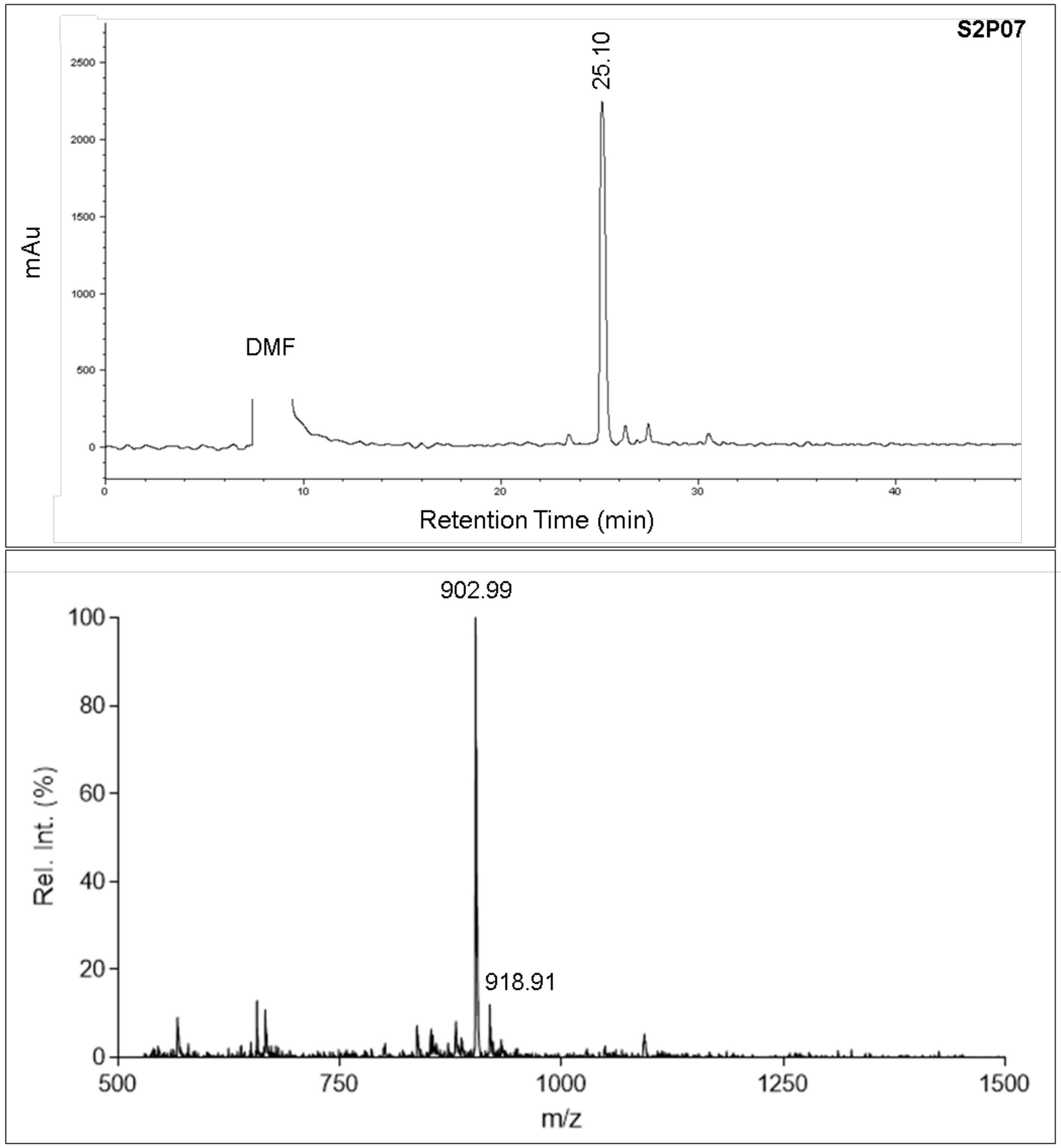

Supplementary Fig. 16 | Characterization data for the peptide S2P07-tBuK. (**Top**) HPLC trace at 215 nm. The peptide was eluted with a linear gradient from 20-50% acetonitrile in water (0.1% TFA) from 10-40 min. (**Bottom**) MALDI-TOF mass spectra. Calculated mass: $[M+H]^+$ = 880.44 Da, $[M+Na]^+$ = 902.41 Da, $[M+K]^+$ = 918.52 Da; Observed Mass: 902.88 Da, 918.91 Da.

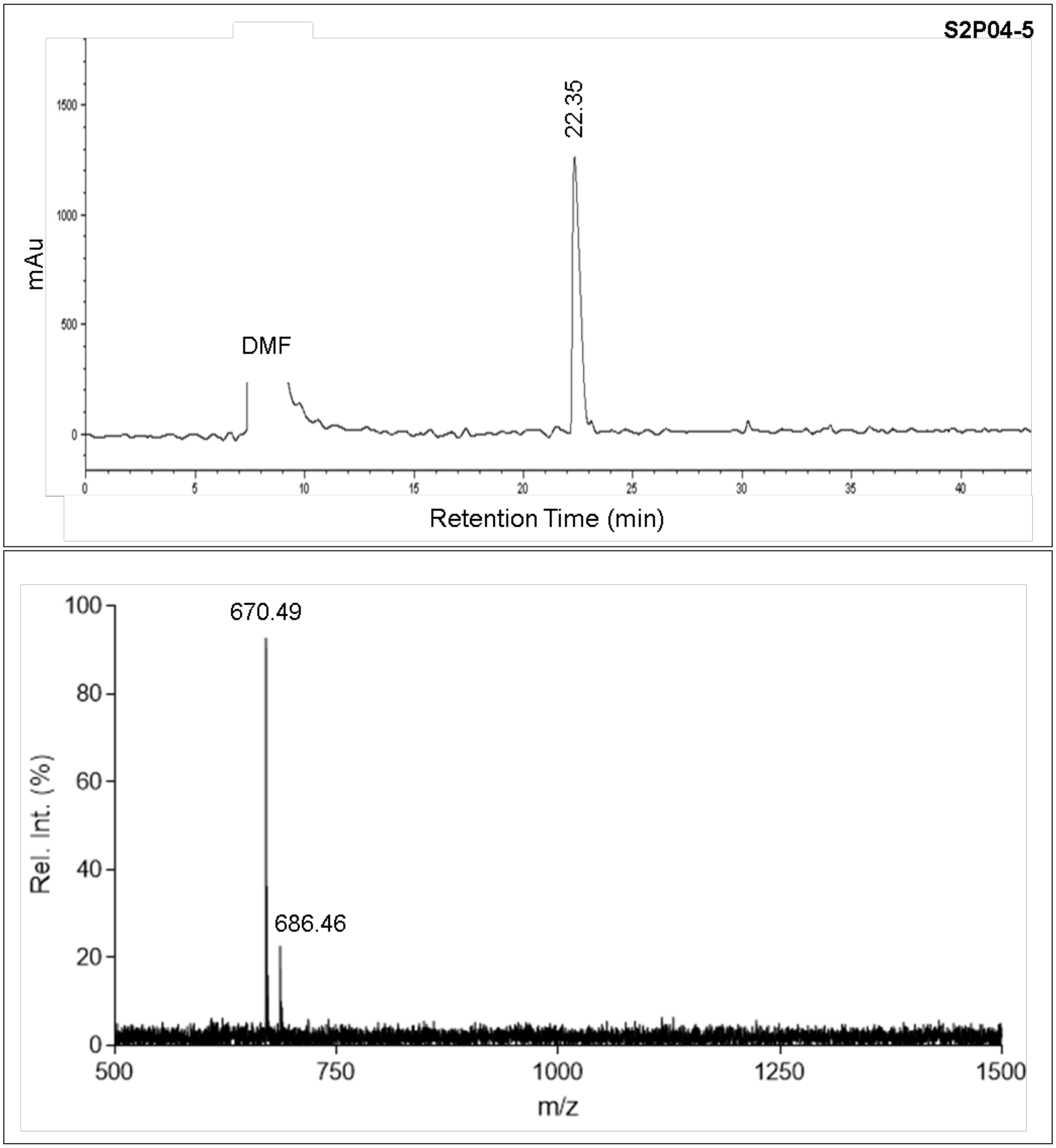

Supplementary Fig. 17 | Characterization data for the peptide S2P04-5-tBuK. (**Top**) HPLC trace at 215 nm. The peptide was eluted with a linear gradient from 20-50% acetonitrile in water (0.1% TFA) from 10-40 min. (**Bottom**) MALDI-TOF mass spectra. Calculated mass: $[M+H]^+$ = 648.36 Da, $[M+Na]^+$ = 670.34 Da, $[M+K]^+$ = 686.45 Da; Observed Mass: 670.49 Da, 686.46 Da.

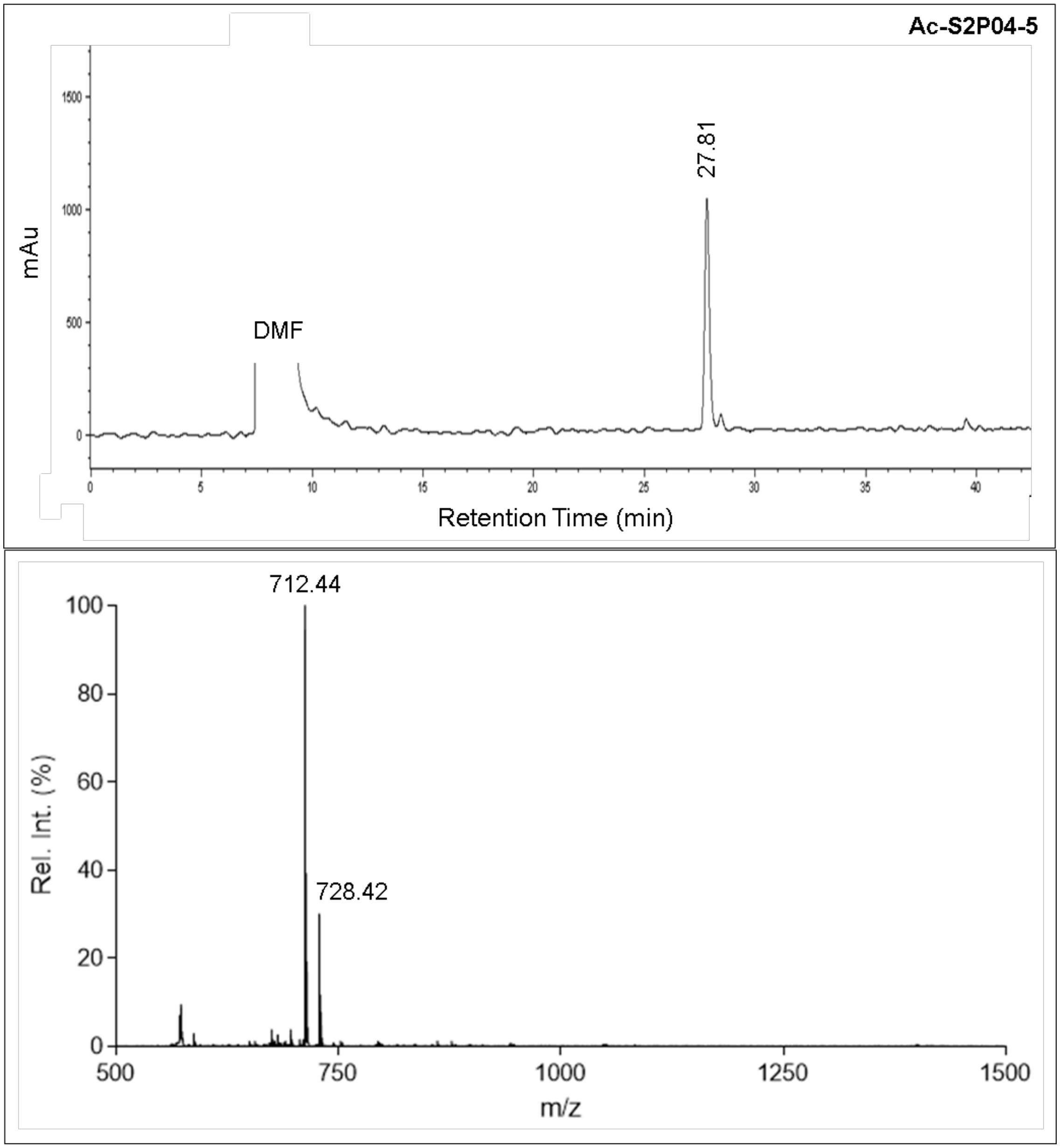

Supplementary Fig. 18 | Characterization data for the peptide Ac-S2P04-5-tBuK. (**Top**) HPLC trace at 215 nm. The peptide was eluted with a linear gradient from 20-50% acetonitrile in water (0.1% TFA) from 10-40 min. (**Bottom**) MALDI-TOF mass spectra. Calculated mass: $[M+H]^+$ = 690.37 Da, $[M+Na]^+$ = 712.35 Da, $[M+K]^+$ = 728.46 Da; Observed Mass: 712.44 Da, 728.42 Da.

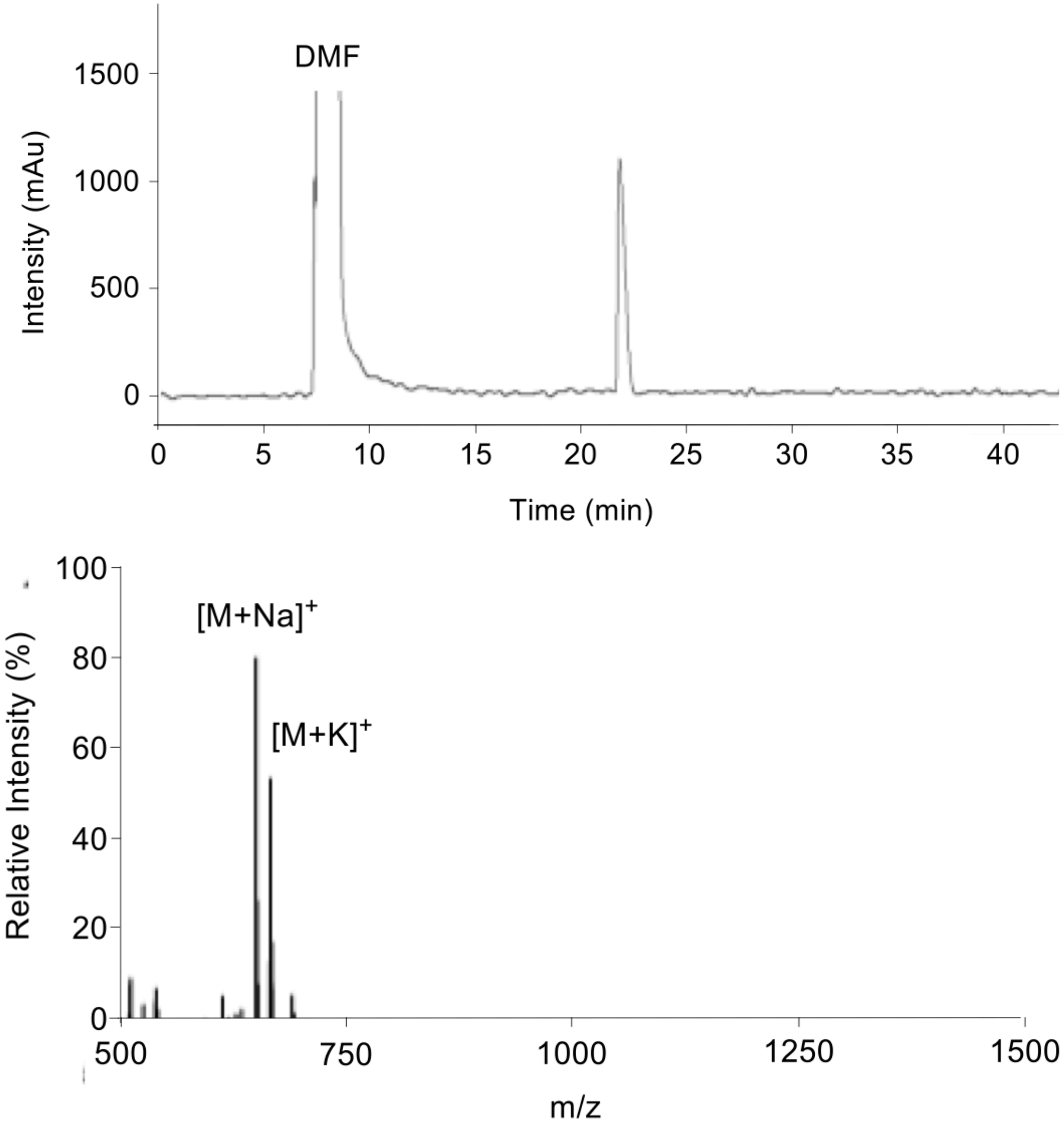

Supplementary Fig. 19 | Characterization data for the peptide S2P04(Abu)-5-tBuK. (**Top**) HPLC trace at 215 nm. The peptide was eluted with a linear gradient from 20-50% acetonitrile in water (0.1% TFA) from 10-40 min. (**Bottom**) MALDI-TOF mass spectra. Calculated mass: $[M+H]^+$ = 630.4 Da, $[M+Na]^+$ = 652.4 Da, $[M+K]^+$ = 668.5 Da; Observed Mass: 653.1 Da, 668.0 Da.

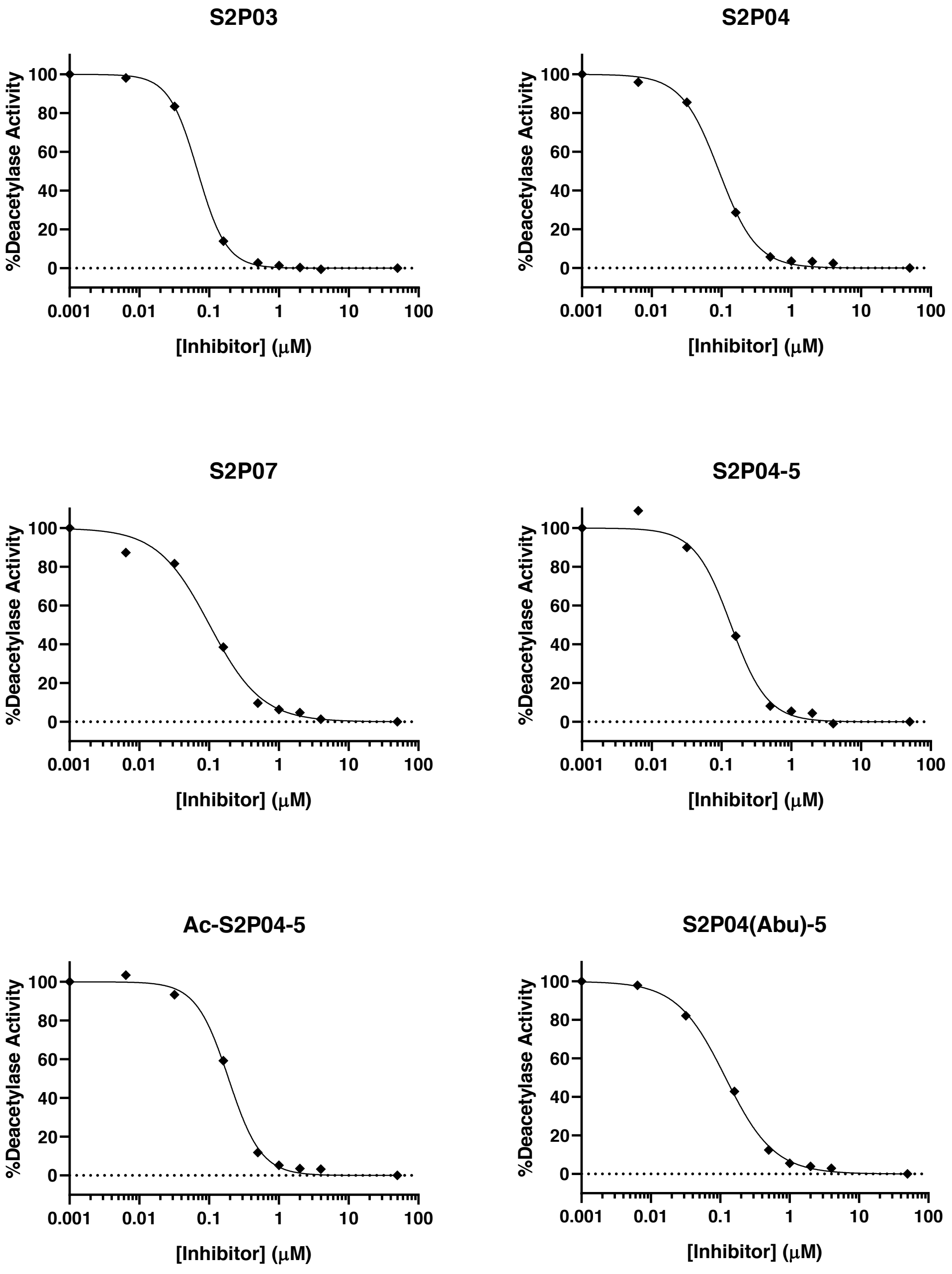

Supplementary Fig. 20 | Inhibition of SIRT2 by synthetic peptides containing tBuK. Reaction mixtures contained 0.25 μM SIRT2, 1 mM NAD^+, 5 μM of fluorogenic substrate, and various concentrations of the peptide inhibitors. Values are given as the mean of 2 biologically independent experiments (*n* = 2).

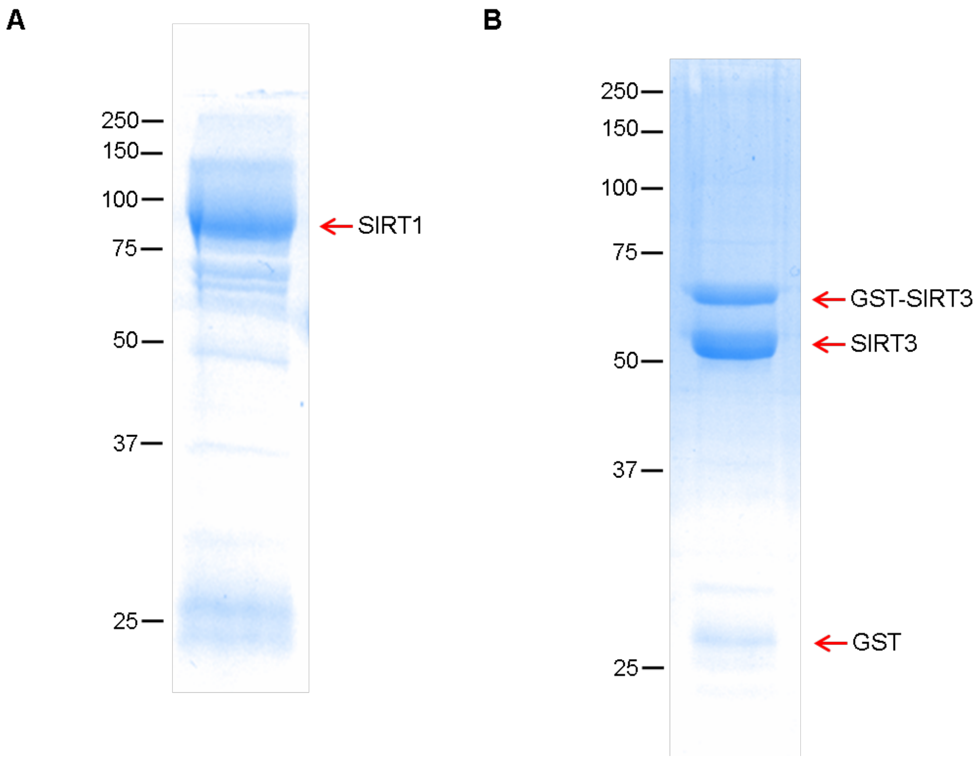

Supplementary Fig. 21 | SDS-PAGE analysis of purified sirtuin 1 and GST-sirtuin 3. **(A)** SDS-PAGE analysis of SIRT1; **(B)** SDS-PAGE analysis of GST-SIRT3. Expression of SIRT1 and GST-SIRT3 has been repeated once successfully. All reported molecular weights are in kDa units.

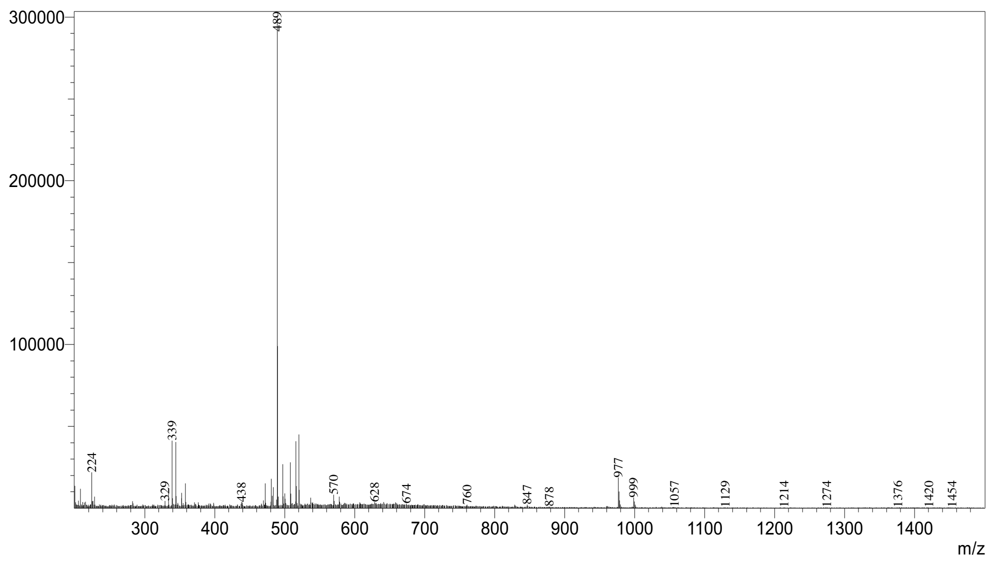

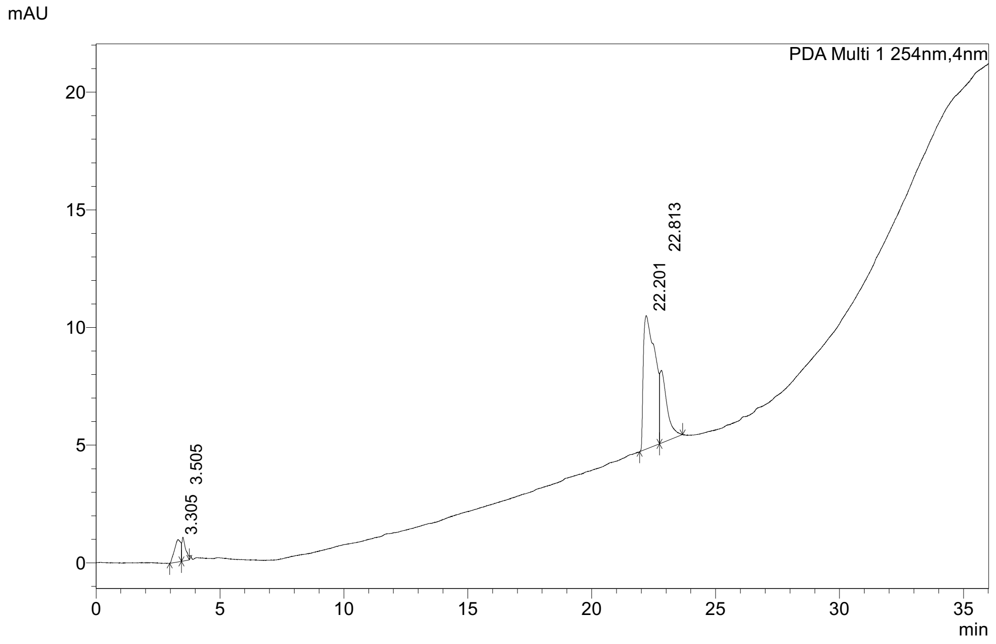

Supplementary Fig. 22 | Characterization data for S2P03-tMyK. Top) ESI-MS from HPLC-MS system. The spectrum consists of an integration over all portions of the chromatogram. **Bottom)** 254 nm trace from isolated product. The peptide was eluted at 22.2 minutes using a 40-70% ACN/Water gradient over 20 minutes (from 5-25 min. on chromatogram).

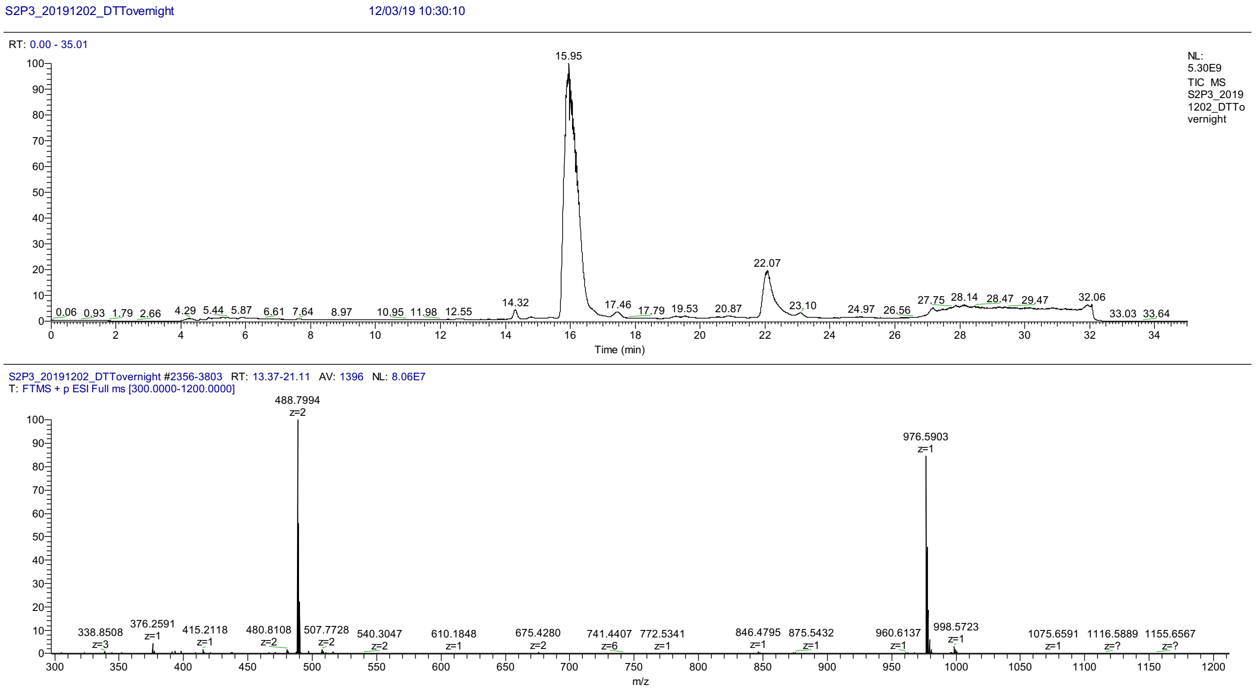

Supplementary Fig. 23 | High Resolution LC-MS for S2P03-tMyK. Mass spectrum corresponds to integration of all mass data from 13.37 minutes to 21.11 minutes. The peak at 22.07 corresponds to disulfide formation and dimerization of the peptide.

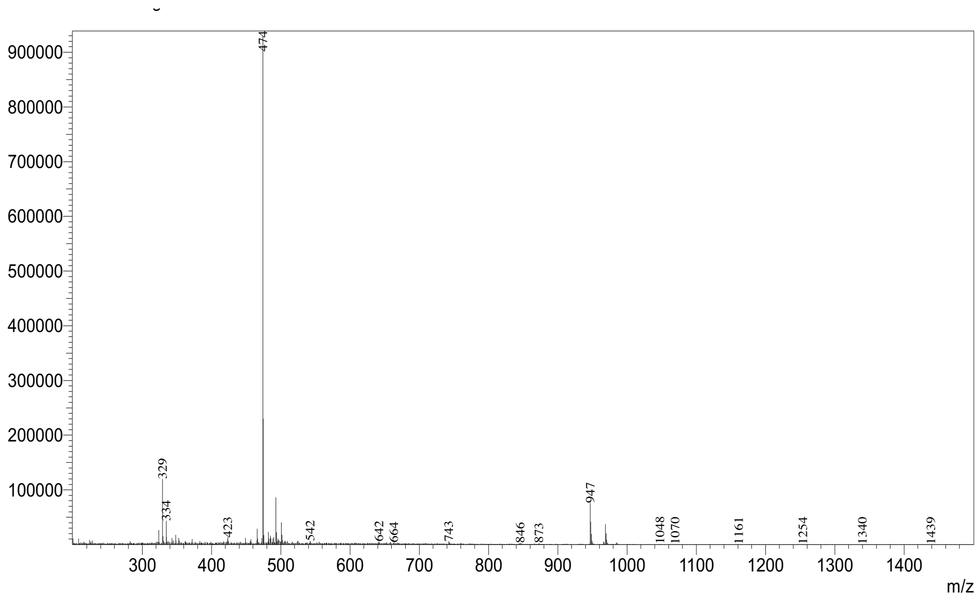

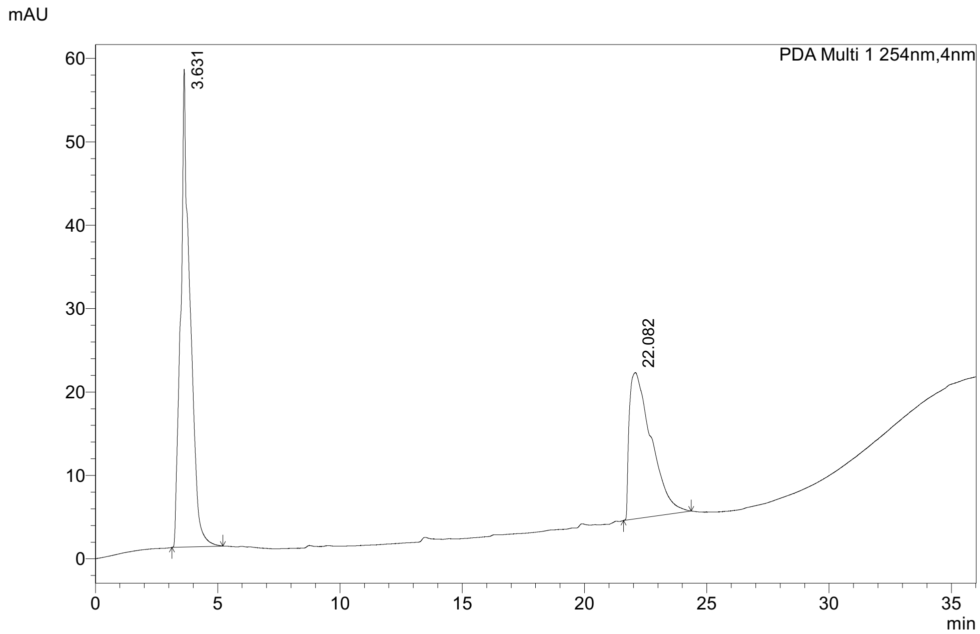

Supplementary Fig. 24 | Characterization data for S2P04-tMyK. Top) ESI-MS from HPLC-MS system. The spectrum consists of an integration over all portions of the chromatogram. **Bottom)** 254 nm trace from isolated product. The peptide was eluted at 22.1 minutes using a 40-70% ACN/Water gradient over 20 minutes (from 5-25 min. on chromatogram). The peak at 3.6 min corresponds to DMF.

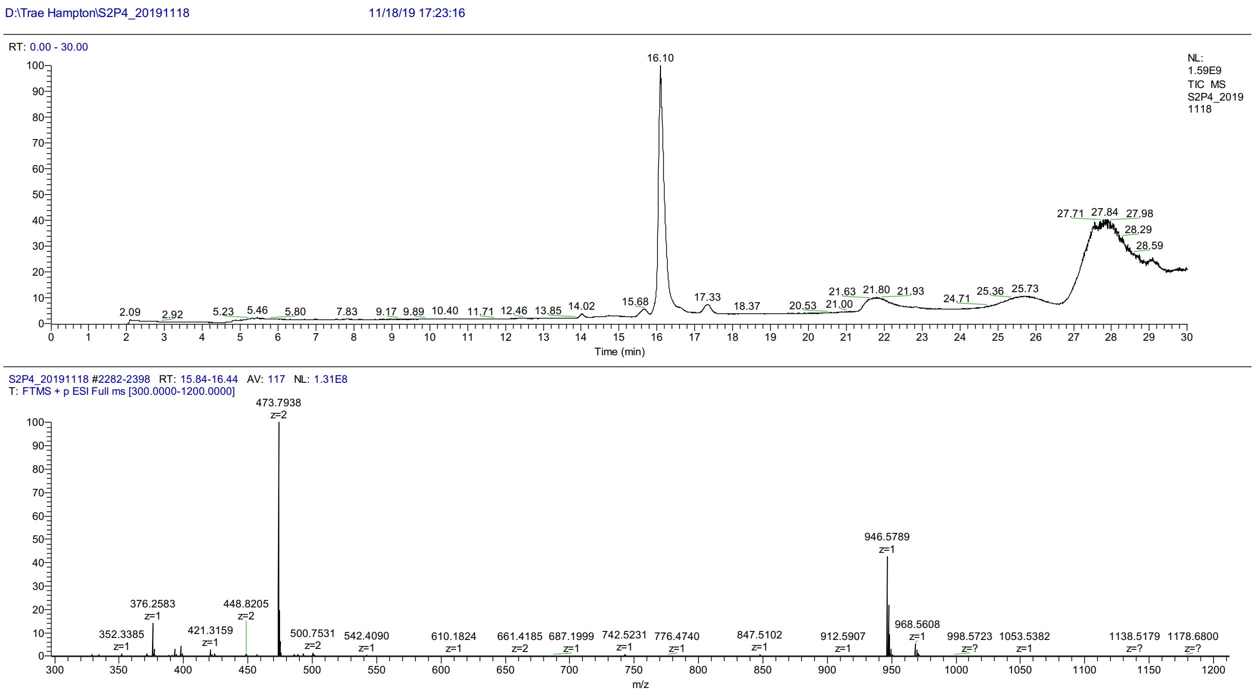

Supplementary Fig. 25 | High Resolution LC-MS for S2P04-tMyK. Mass spectrum corresponds to integration of all mass data from 15.84 minutes to 16.44 minutes.

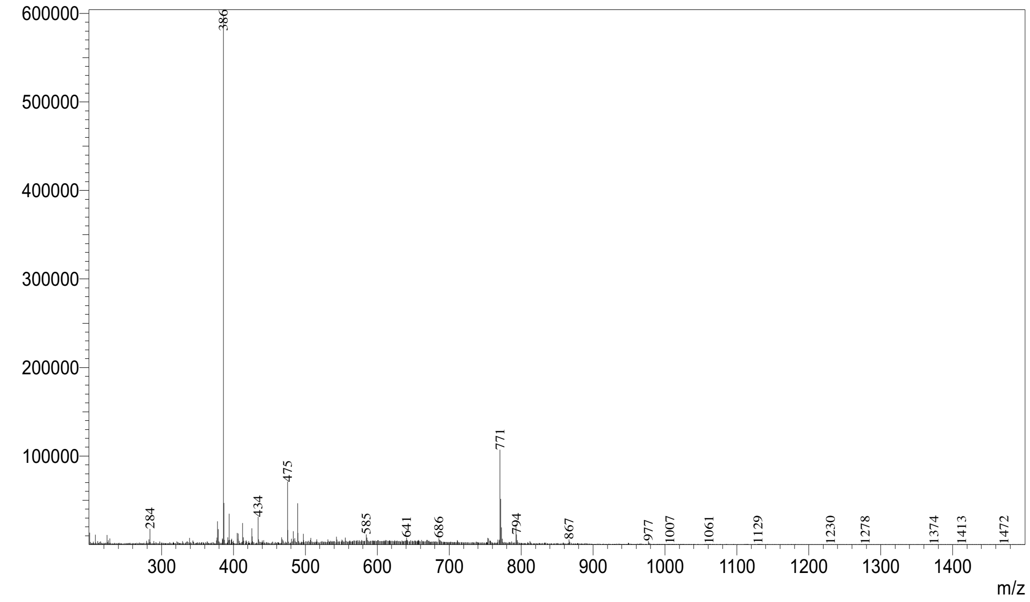

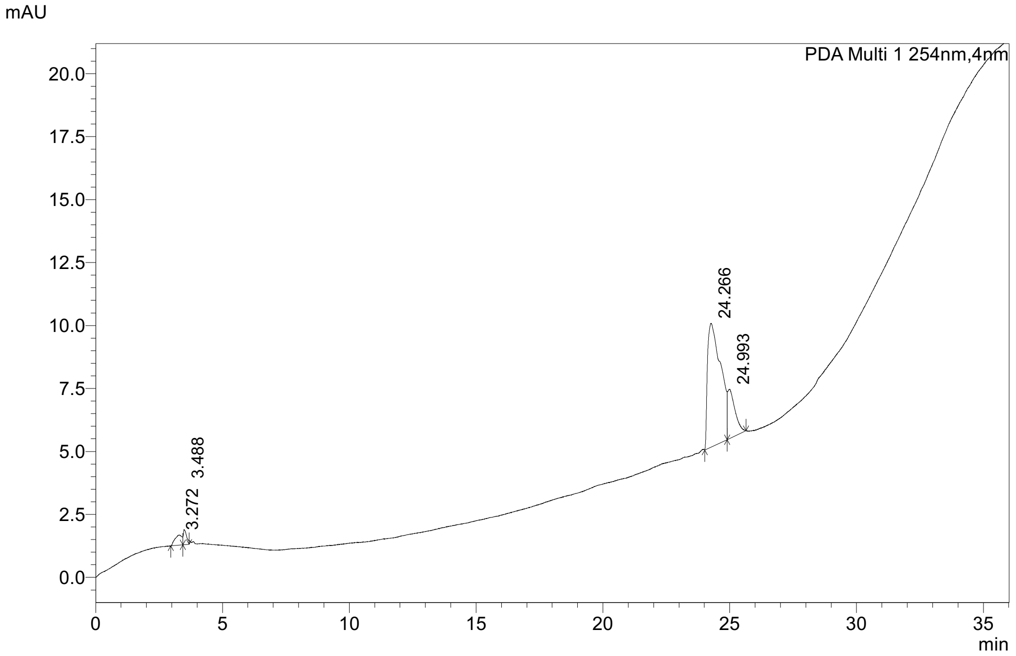

Supplementary Fig. 26 | Characterization data for S2P04(Abu)-5-tMyK. Top) ESI-MS from HPLC-MS system. The spectrum consists of an integration over all portions of the chromatogram. **Bottom)** 254 nm trace from isolated product. The peptide was eluted at 24.3 minutes using a 40-70% ACN/Water gradient over 20 minutes (from 5-25 min. on chromatogram).

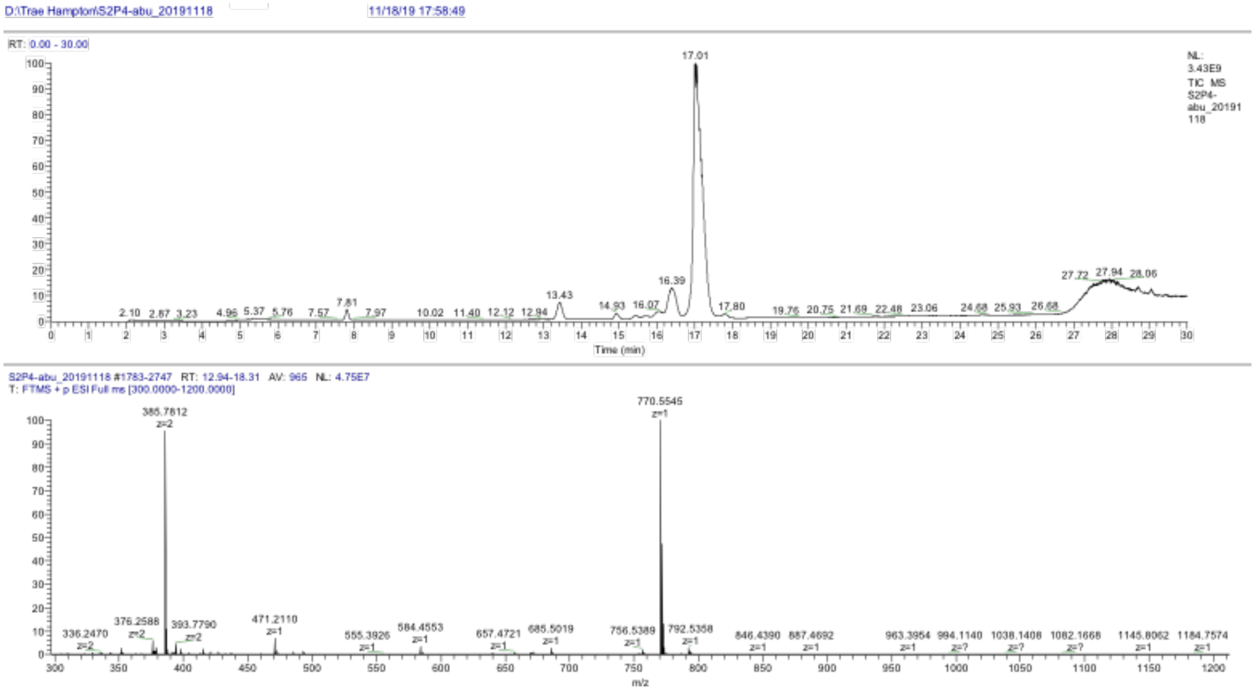

Supplementary Fig. 27 | High Resolution LC-MS for S2P04(Abu)-5-tMyK. Mass spectrum corresponds to integration of all mass data from 12.94 minutes to 18.31 minutes.

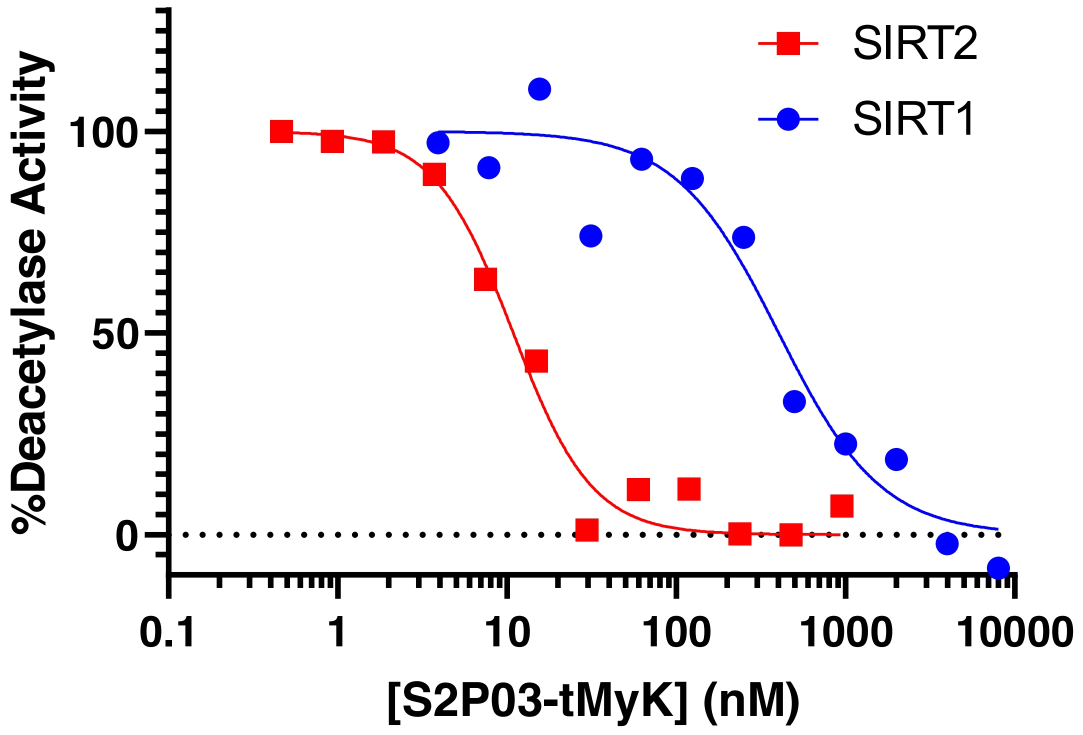

Supplementary Fig. 28 | Inhibition of SIRT2 and SIRT1 by S2P03-tMyK. Values are represented by the mean of n = 2 biologically independent experiments. Data for this graph is available in the Source Data File.

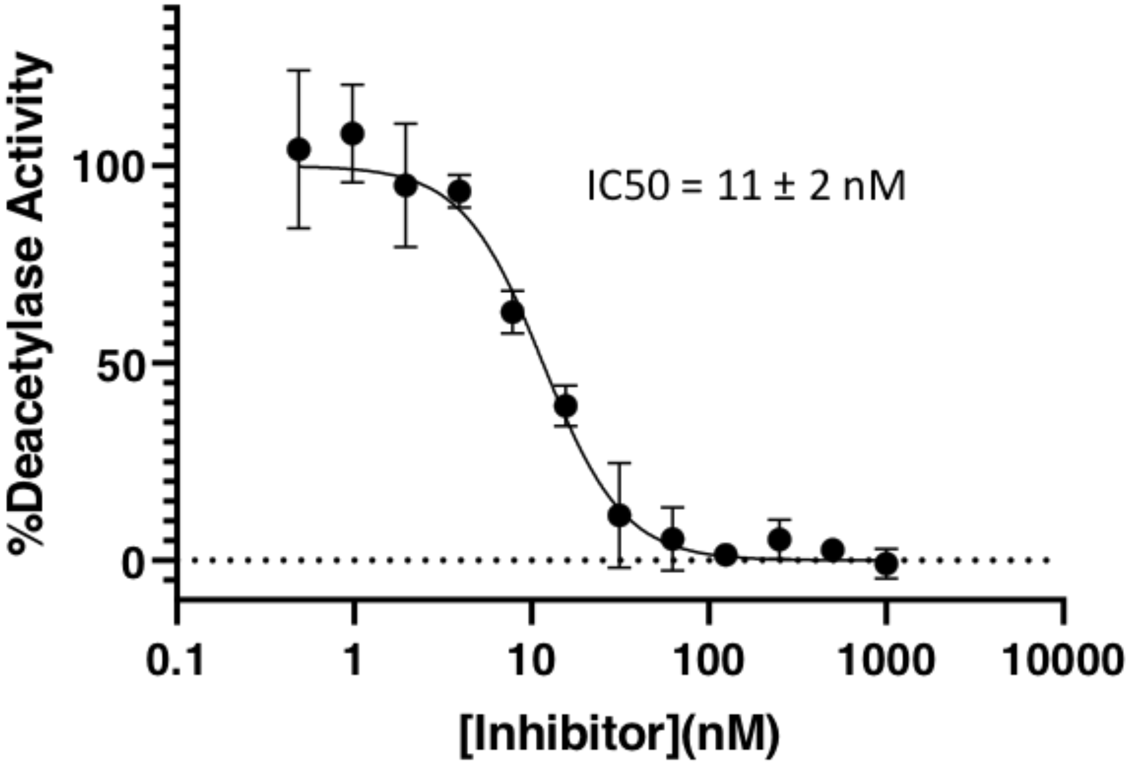

Supplementary Fig. 29 | Inhibition of SIRT2 by S2P03-tMyK with no preincubation. Error bars represent ± one standard deviation from the mean for *n* = 4 biologically independent experiments. Data for this curve is available in the Source Data File.

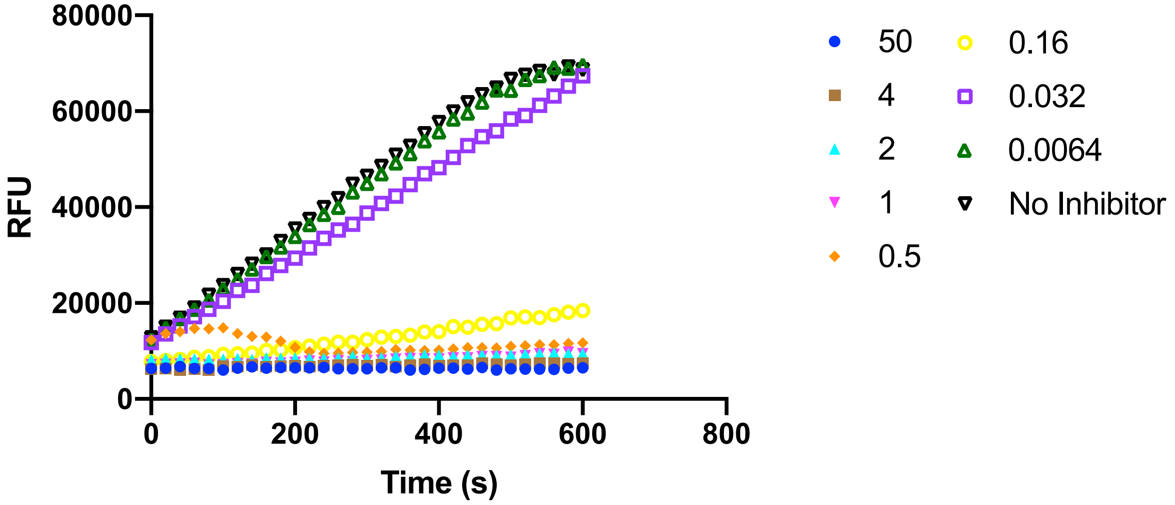

Supplementary Fig. 30 | Inhibition Curves for Inhibition of SIRT2 by S2P03-tBu. With the continuous assay, fluorescence intensity of active samples plateaus after around 8 minutes, preventing kinetic studies over a long period of time. Each curve corresponds to a concentration of S2P03-tBu in μM.

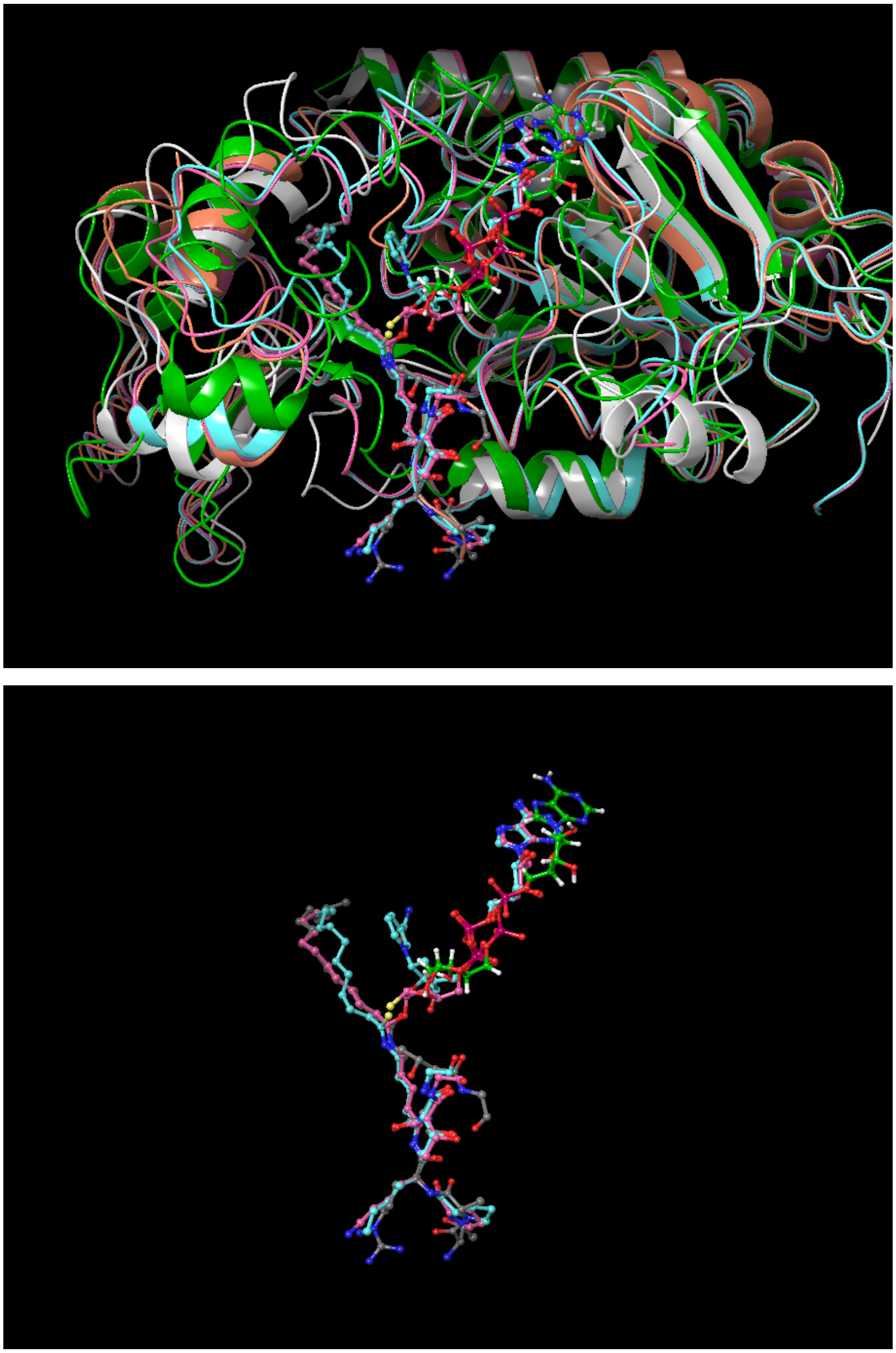

Supplementary Fig. 31 | Molecular overlays of available SIRT2 crystal structures. Top, A molecular overlay of available SIRT2 structures from the PDB (3ZGO, 3ZGV, 4X3O, 4X3P, and 4Y6L) showing a number of flexible loop regions. **Bottom,** An overlay of the ligands illustrating that the binding pocket shape is mostly conserved with the different ligands.

S2P03-tBuK

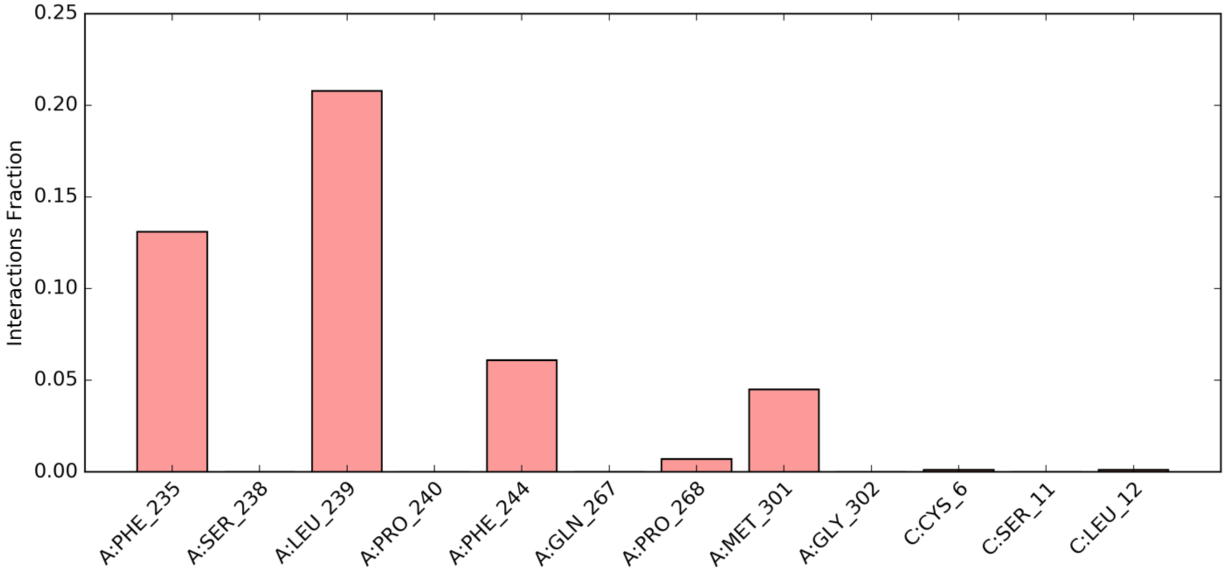

Interactions Fraction
0.25
0.20
0.15
0.10
0.05
0.00
A:PHE_235
A:SER_238
A:LEU_239
A:PRO_240
A:PHE_244
A:GLN_267
A:PRO_268
A:MET_301
A:GLY_302
C:CYS_6
C:SER_11
C:LEU_12

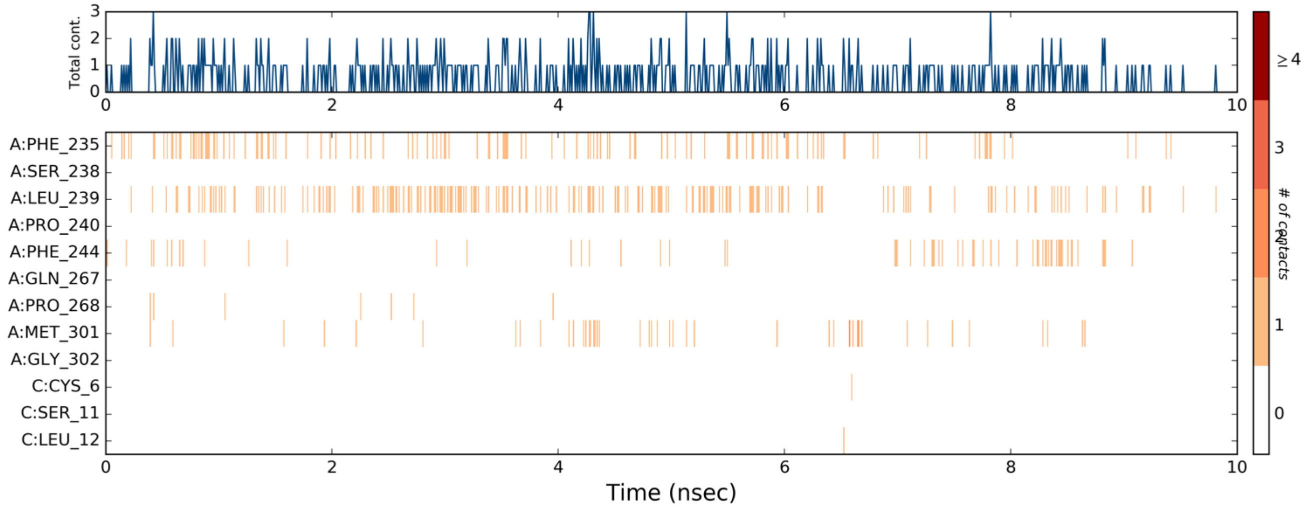

Total cont.
A:PHE_235
A:SER_238
A:LEU_239
A:PRO_240
A:PHE_244
A:GLN_267
A:PRO_268
A:MET_301
A:GLY_302
C:CYS_6
C:SER_11
C:LEU_12
Time (nsec)
# of contacts
≥ 4

S2P04-tBuK

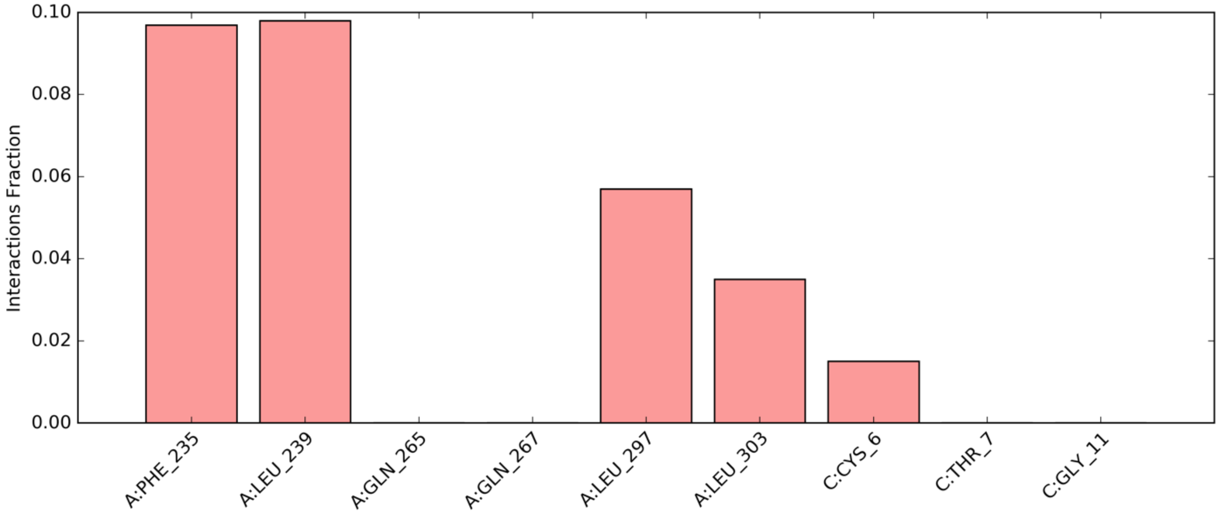

Interactions Fraction
0.00
0.02
0.04
0.06
0.08
0.10
A:PHE_235
A:LEU_239
A:GLN_265
A:GLN_267
A:LEU_297
A:LEU_303
C:CYS_6
C:THR_7
C:GLY_11

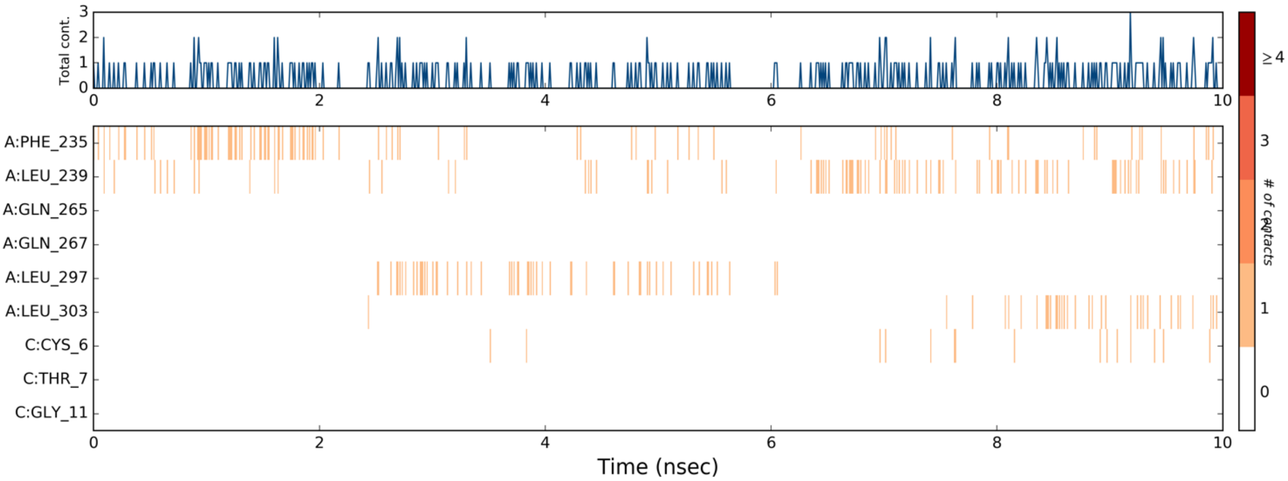

Total cont.
A:PHE_235
A:LEU_239
A:GLN_265
A:GLN_267
A:LEU_297
A:LEU_303
C:CYS_6
C:THR_7
C:GLY_11
Time (nsec)
# of contacts
≥ 4

S2P07-tBuK

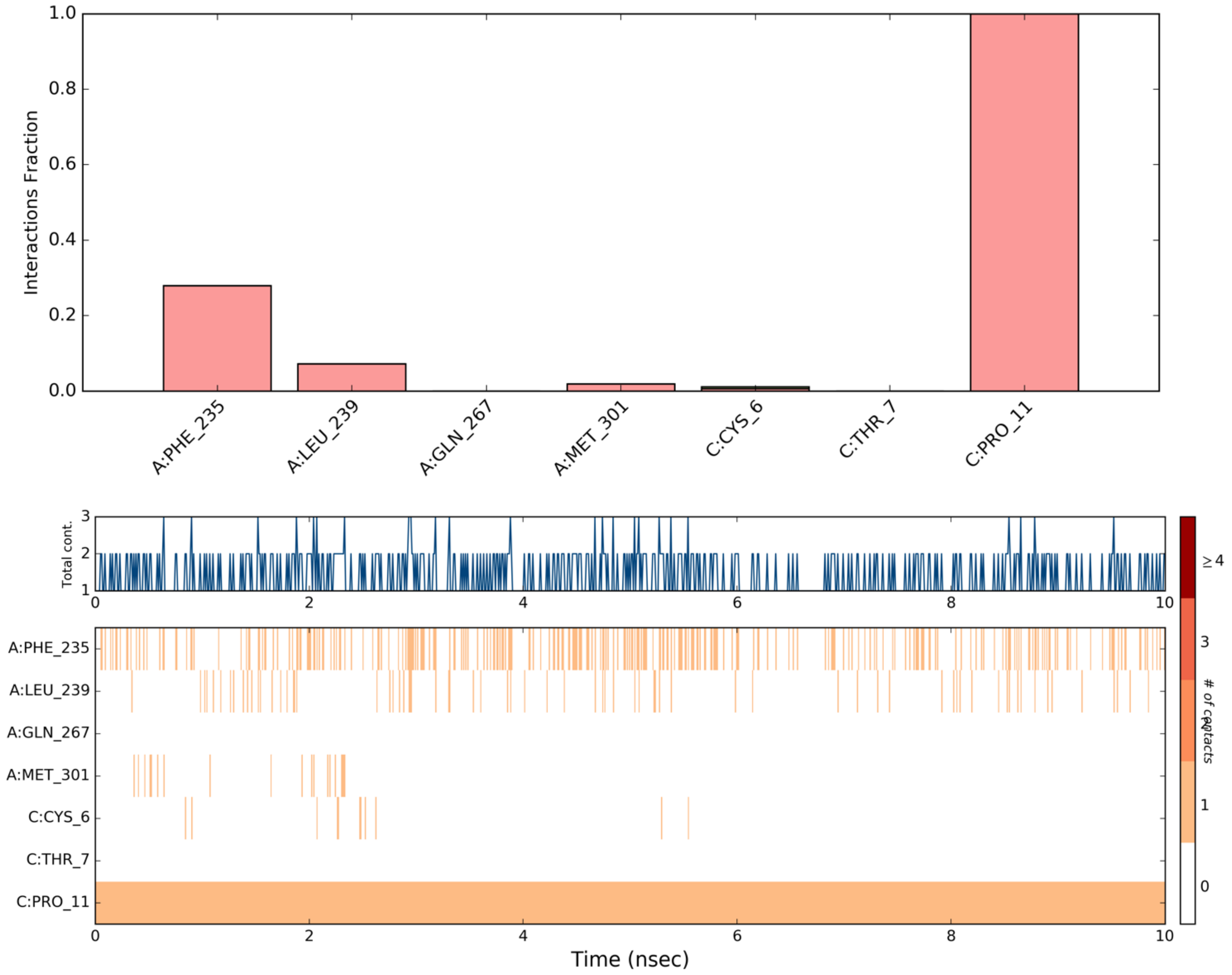

Interactions Fraction
1.0
0.8
0.6
0.4
0.2
0.0
A:PHE_235
A:LEU_239
A:GLN_267
A:MET_301
C:CYS_6
C:THR_7
C:PRO_11
Total cont.
A:PHE_235
A:LEU_239
A:GLN_267
A:MET_301
C:CYS_6
C:THR_7
C:PRO_11
Time (nsec)
# of contacts
≥ 4
3
1
0

S2P04-5-tBuK

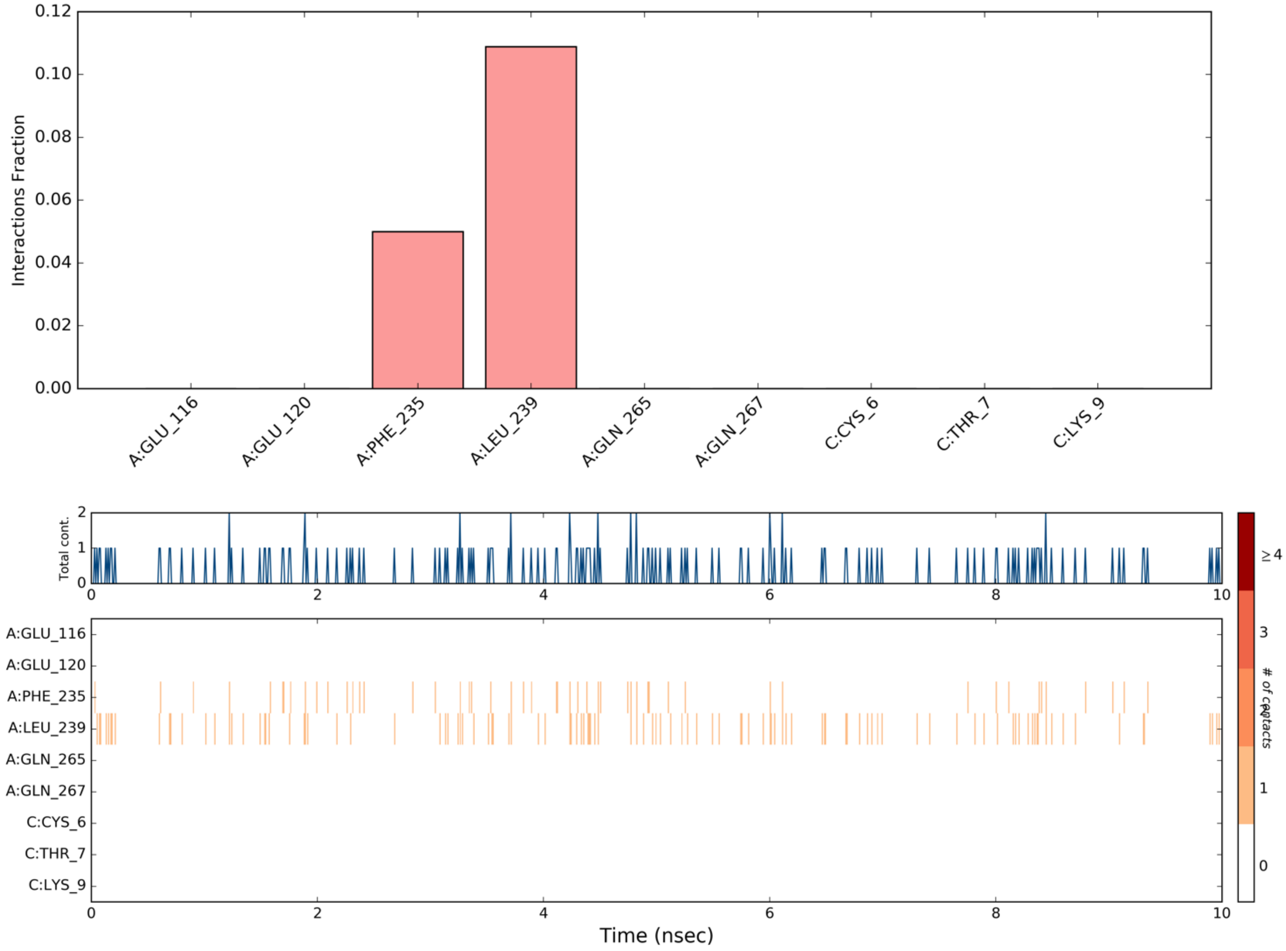

Interactions Fraction
0.12
0.10
0.08
0.06
0.04
0.02
0.00
A:GLU_116
A:GLU_120
A:PHE_235
A:LEU_239
A:GLN_265
A:GLN_267
C:CYS_6
C:THR_7
C:LYS_9
Total cont.
Time (nsec)
# of contacts
≥ 4

Ac-S2P04-5-tBuK

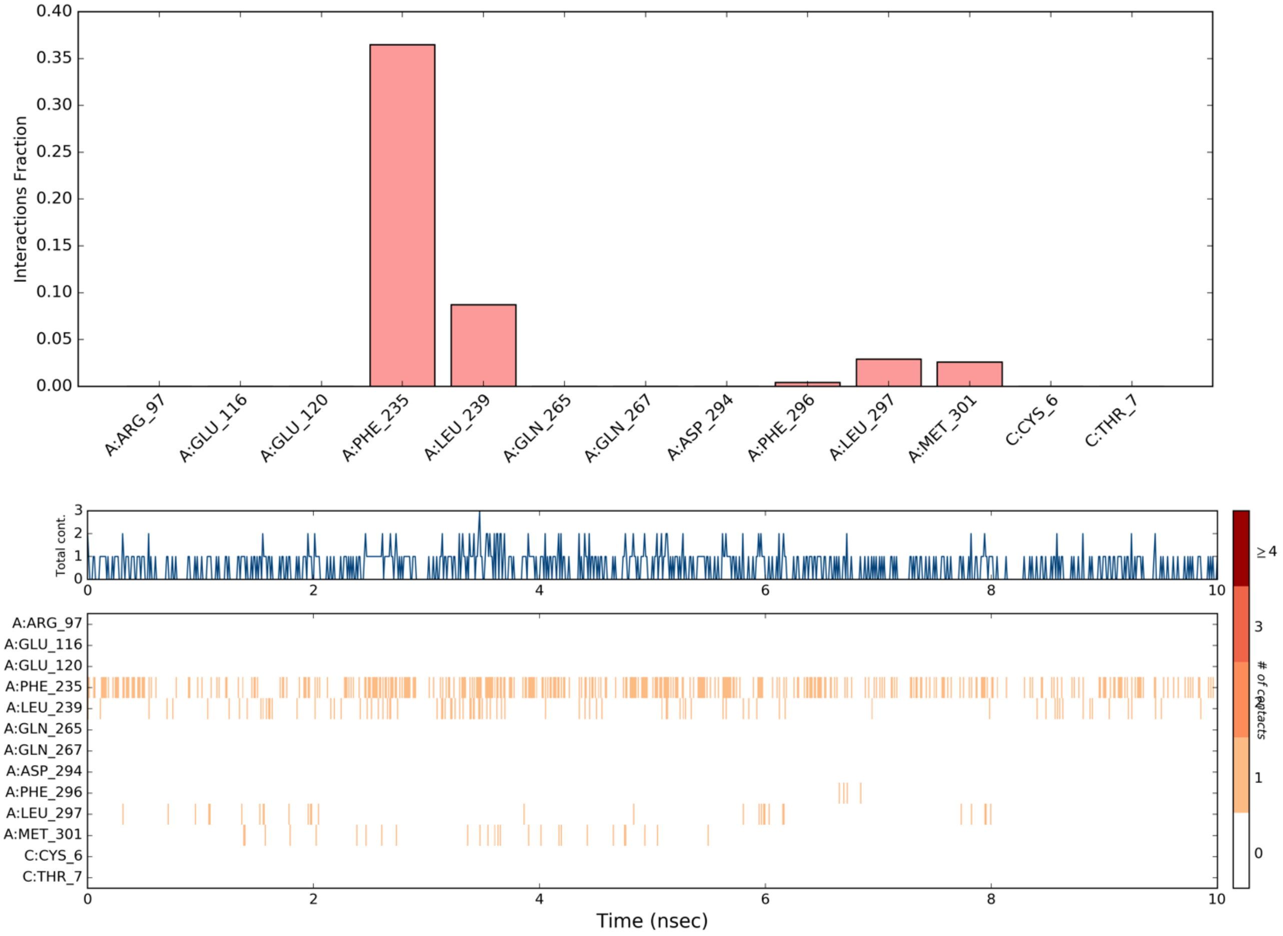

Supplementary Fig. 32 | Histograms displaying hydrophobic interactions between SIRT2 and peptide inhibitors. The histograms (top) indicate which residues in the protein (chain A) or peptide (chain C) interact with the residues adjacent to tBuK in the peptide ligand. The bottom panels display each frame that the interaction was observed over the 1000-frame simulation.

^{1}H NMR spectrum of compounds **13**

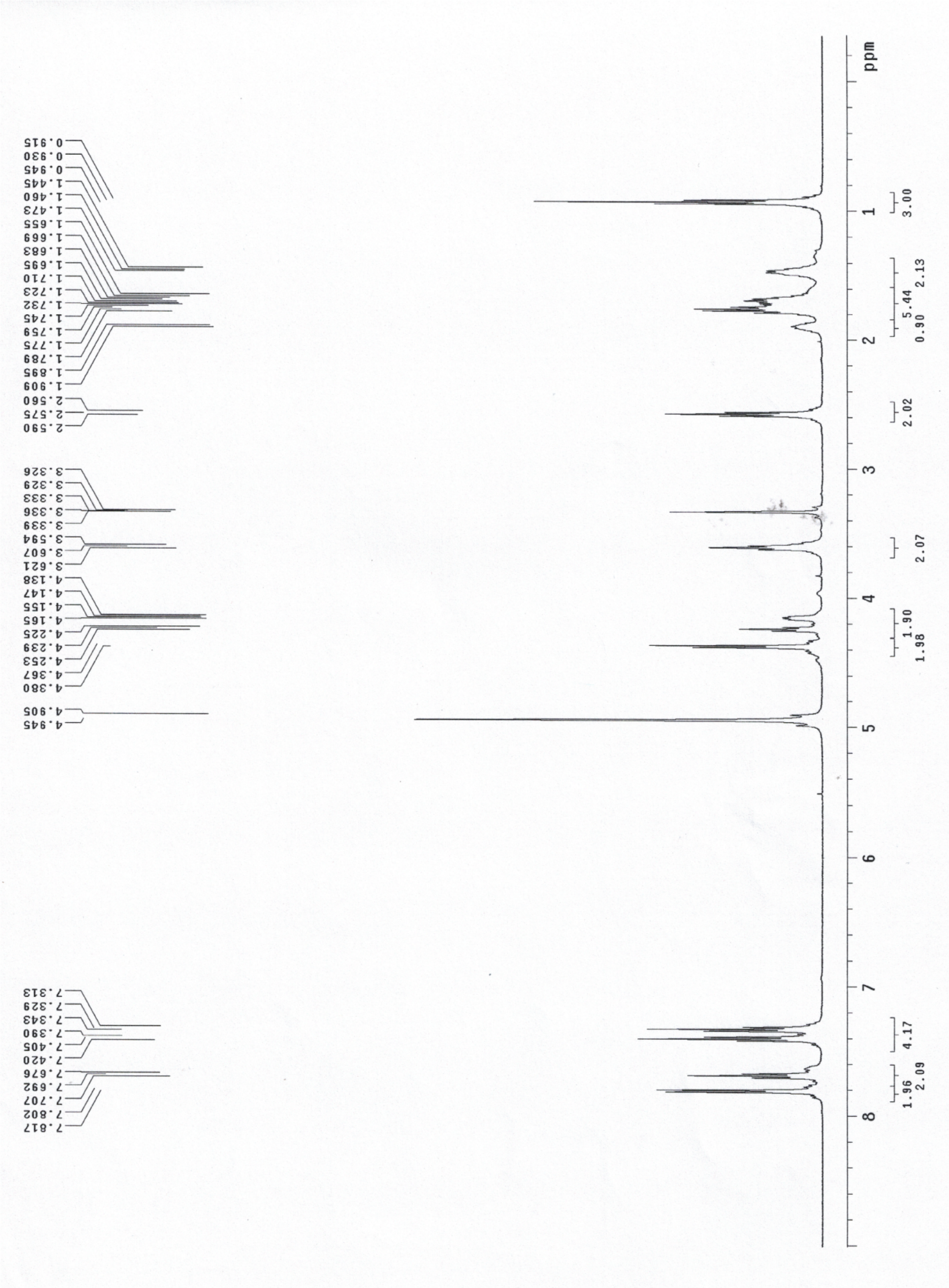

^{13}C NMR spectrum of compound **13**

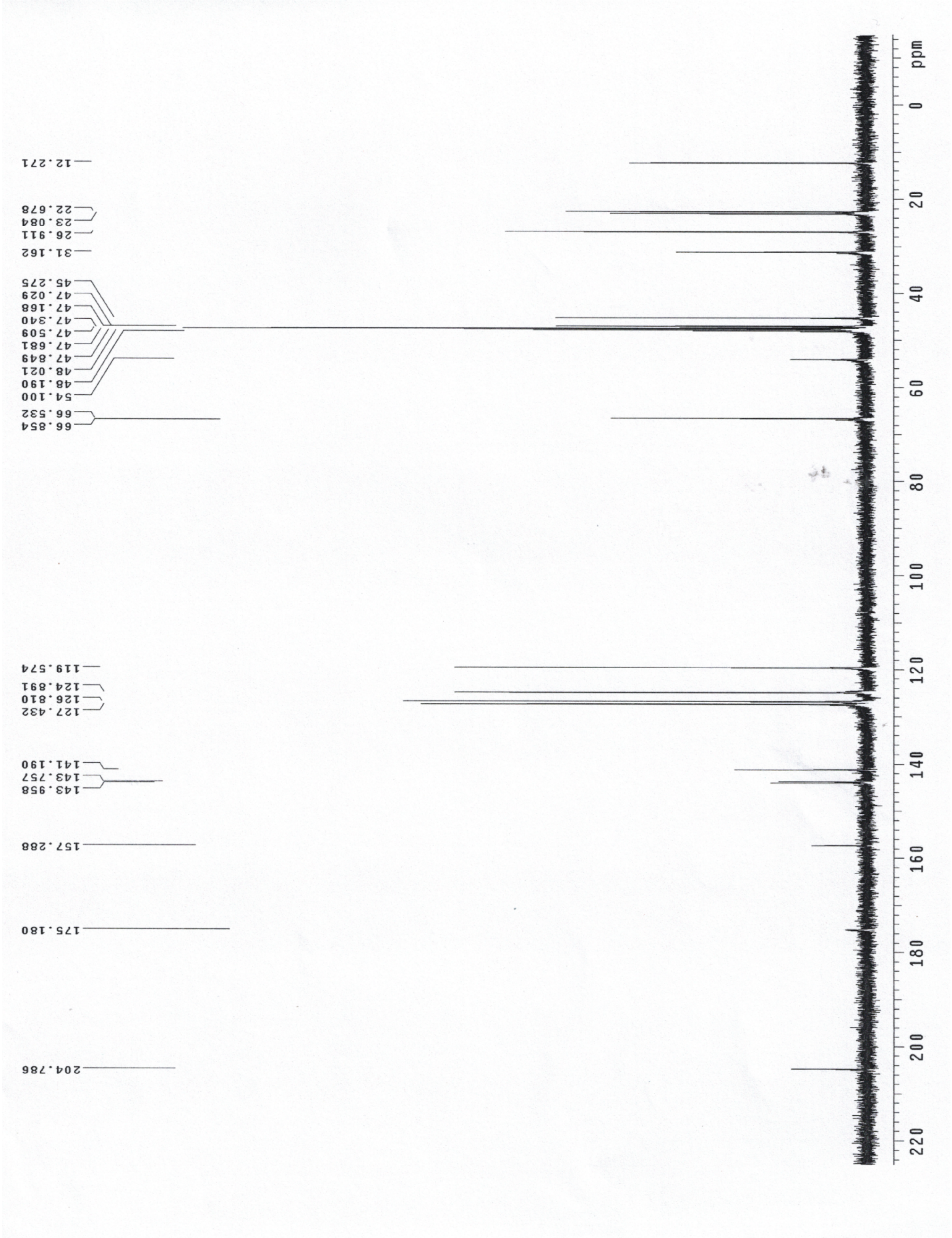

^{1}H NMR spectrum of compound **19**

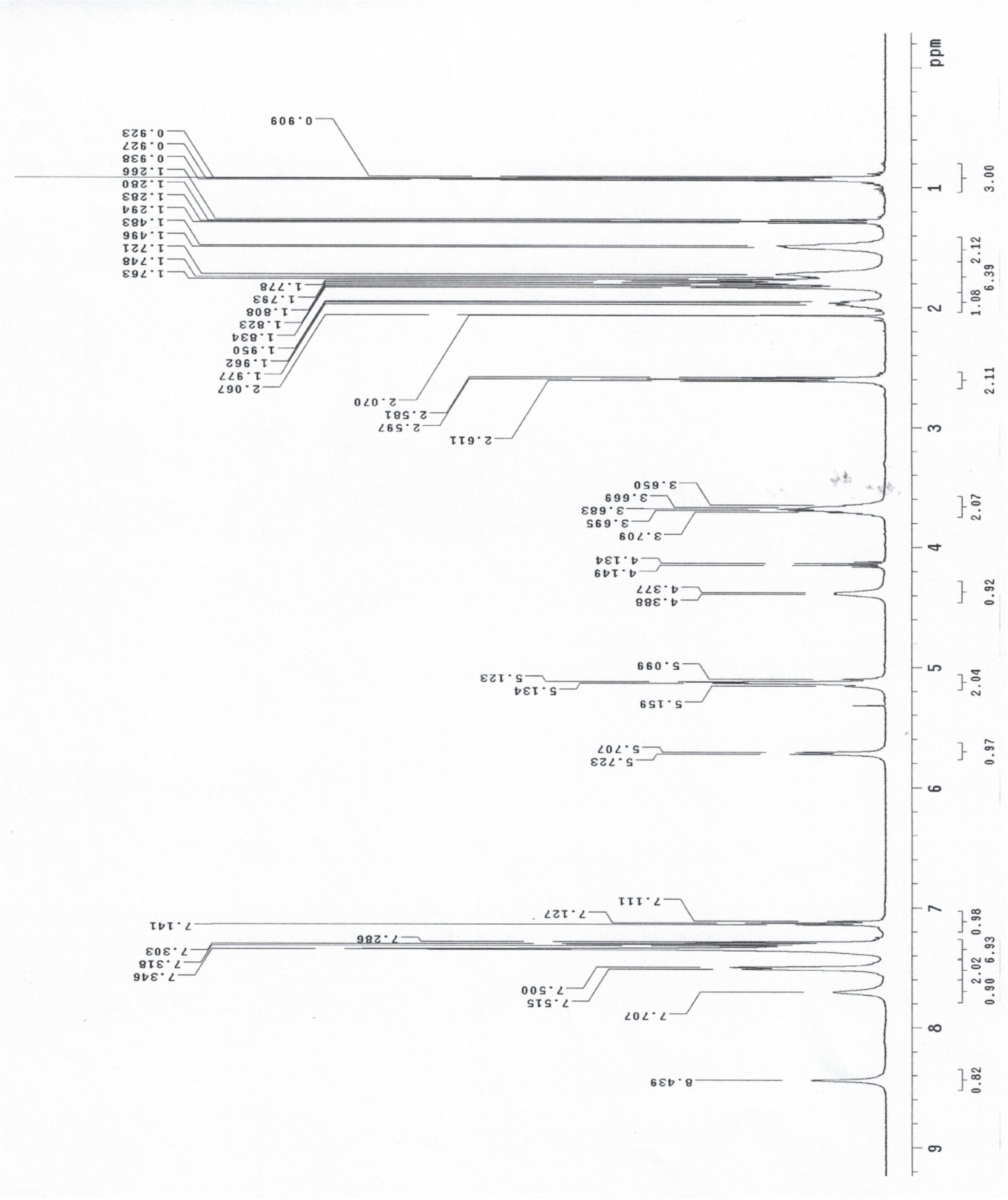

^{13}C NMR spectrum of compound **19**

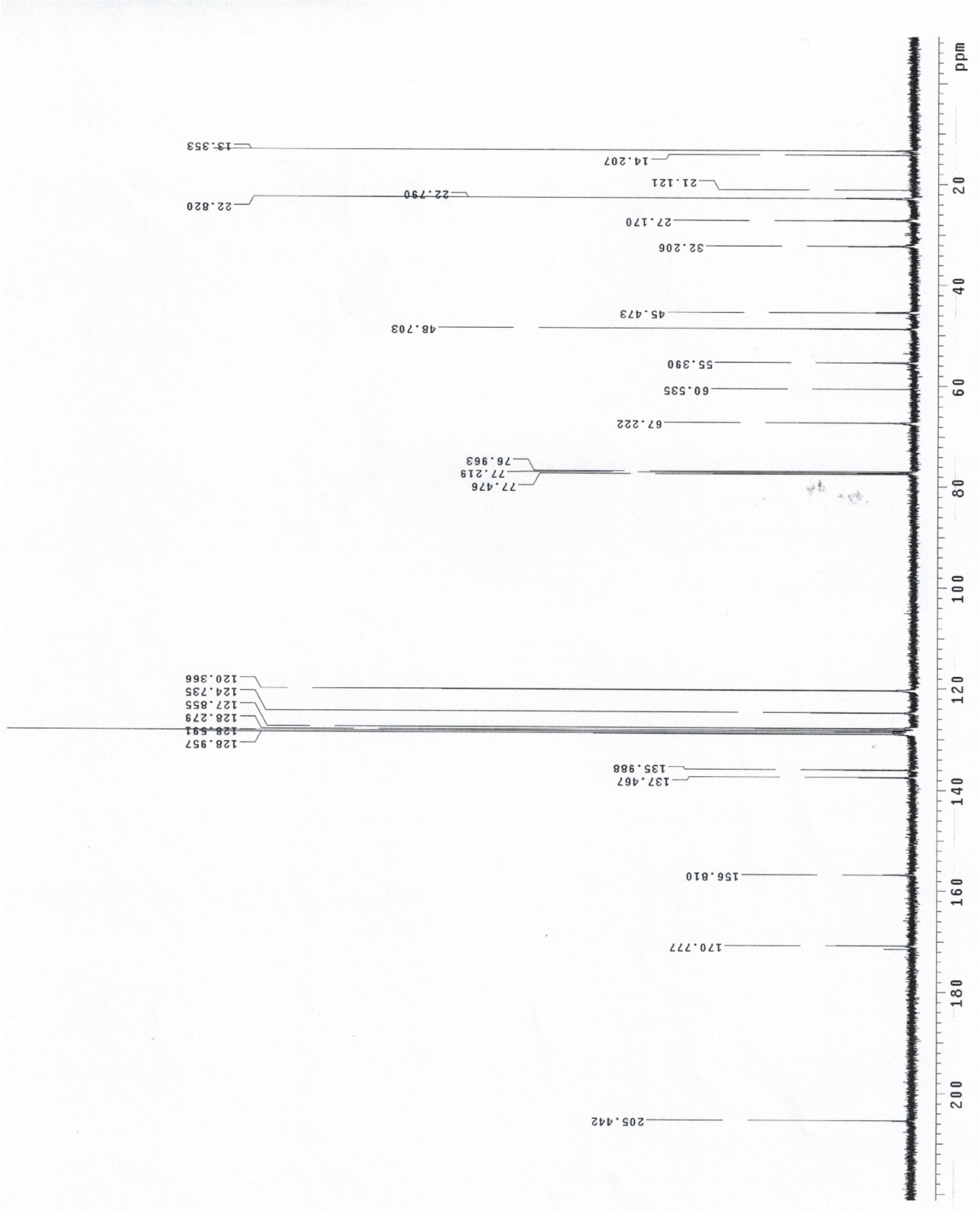

^{1}H NMR spectrum of compound **21**

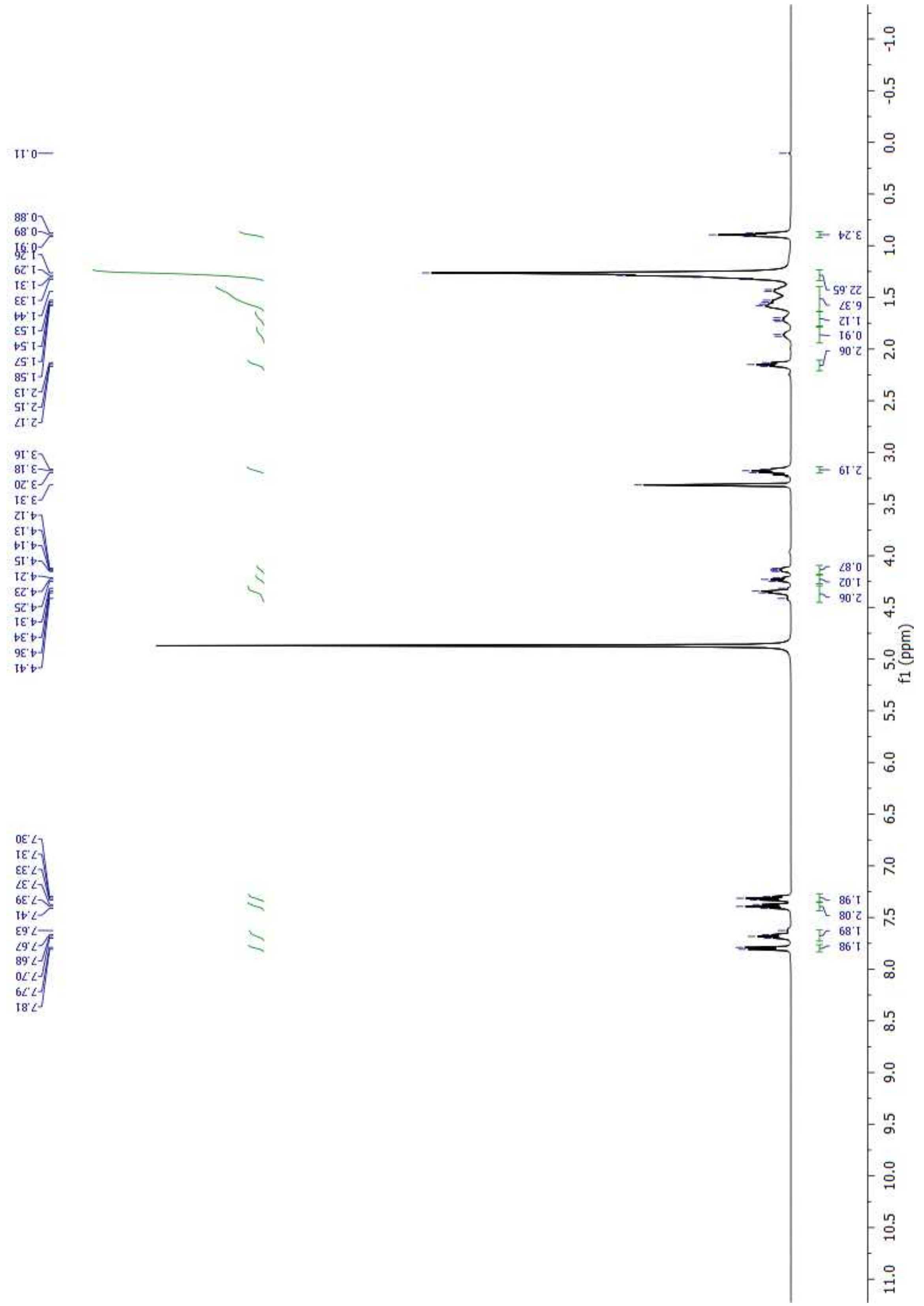

^{1}H NMR spectrum of compound **22**

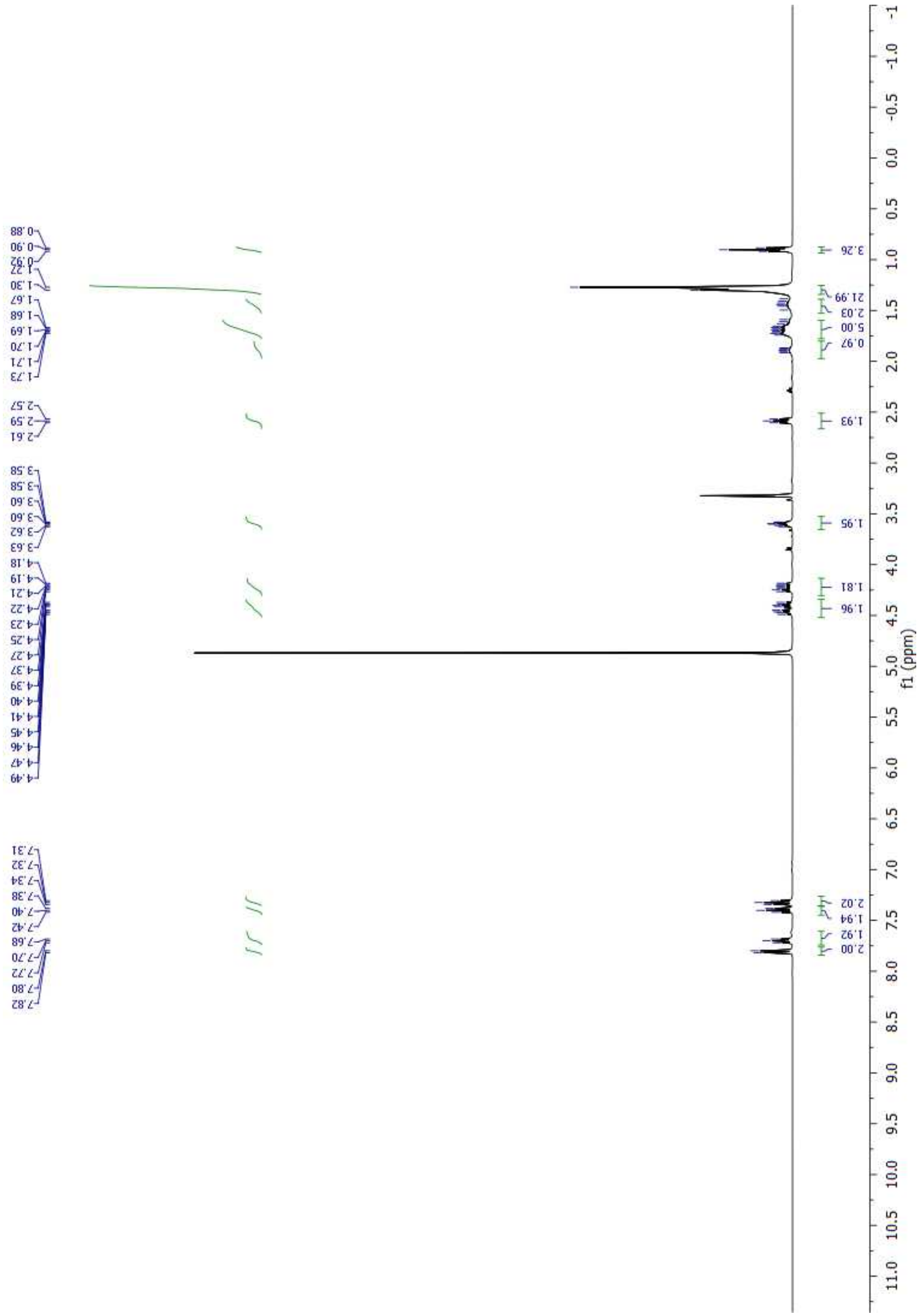

SUPPLEMENTARTY R SCRIPTS

Suppementary R Script 1

Amino Acid Analysis.R

```
library(microseq)
library(RColorBrewer)
library(dplyr)
library(stringr)
library(gplots)
NNK7Ffilt <- readFastq("/Users/traehampton/Desktop/NGS
FastQ/filtered/Users/traehampton/Desktop/NGS FastQ/1_S1_L001_R1_001.fastq_F2_filt2.fastq")
NNK7Rfilt <- readFastq("/Users/traehampton/Desktop/NGS
FastQ/filtered/Users/traehampton/Desktop/NGS FastQ/1_S1_L001_R2_001.fastq_R2_filt2.fastq")
#Define the following variables
libraryseq <- "GCCCAG.{33}GCGGCG.{6}" #change this regex to match specific library
beginning <- 19 #beginning of library in DNA string
end <- 40 #end of library in DNA string
initialcodon <- 23 #initial position of library with spaces between codons
endcodon <- 51 #terminal position of library with spaces between codons
lib <- 7 #number of codons in the library region
del <- 6 #number of codons before library


#slices out matches that contain start followed by 24 bases to reverse primer
NNK7Ffilt21 <- gregexpr(libraryseq,NNK7Ffilt[,2],extract = TRUE)
NNK7Rrevcomp <- reverseComplement(NNK7Rfilt[,2],reverse = TRUE) #gives reverse complement
of reverse reads
NNK7Rcompfilt21 <- gregexpr(libraryseq,NNK7Rrevcomp,extract = TRUE)

#this compares the forward and reverse strands, only allowing for one mismatch in the
primers, no mismatches allowed in the library region
n <- length(NNK7Ffilt21)
NNK7Fgood <- vector()
for(i in c(1:n)){
  if(NNK7Ffilt21[[i]][1] == NNK7Rcompfilt21[[i]][1]){
    NNK7Fgood[i] <- NNK7Ffilt21[[i]][1]
  }
  else{
    split <- strsplit(c(NNK7Ffilt21[[i]][1],NNK7Rcompfilt21[[i]][1]), split = "")
    diff <- which(split[[1]] != split[[2]])
    if(length(diff) < 2 && length(diff) > 0){
      for(x in c(1:length(diff))){
        if(diff[[x]] < beginning || diff[[x]] > end){
          NNK7Fgood[i] <- NNK7Ffilt21[[i]][1]
        }
        else{
          NNK7Fgood[i] <- ""
        }
      }
    }
    else{
      NNK7Fgood[i] <- ""
    }
  }
}
NNK7Fgood <- as.data.frame(NNK7Fgood)
NNK7Fgood <- NNK7Fgood[!apply(is.na(NNK7Fgood) | NNK7Fgood == "", 1, all),]

#this separates nucleotides into codons
codons <- gsub("(...)", "\\1 \\2", NNK7Fgood)

#this creates dataframe of sequences with reads organized by frequency
seqcount <- as.data.frame(sort(table(codons), decreasing = TRUE))
```

```
#this generates a matrix that contains amino acids in library region
l <- length(codons)
AAs <- matrix(0,l,lib)
Phereg <- gregexpr("\\s(TT[TC])",codons,useBytes = FALSE)
  lphe <- length(Phereg)
  for(a in c(1:lphe)){
    lphee <- length(Phereg[[a]])
    for(b in c(1:lphee)){
      value <- Phereg[[a]][b]
      if(value > initialcodon && value < endcodon){
        AAs[a,(value%/%4 - (del-1))] <- "F"
      }
    }
  }
Leureg <- gregexpr("(\\sTT[AG])|(\\sCT[GACT])",codons,useBytes = FALSE)
  lleu <- length(Leureg)
  for(a in c(1:lleu)){
    lleuu <- length(Leureg[[a]])
    for(b in c(1:lleuu)){
      value <- Leureg[[a]][b]
      if(value > initialcodon && value < endcodon){
        AAs[a,(value%/%4 - (del-1))] <- "L"
      }
    }
  }
Serreg <- gregexpr("(\\sTC[GCAT])|(\\sAG[TC])",codons,useBytes = FALSE)
  lser <- length(Serreg)
  for(a in c(1:lser)){
    lserr <- length(Serreg[[a]])
    for(b in c(1:lserr)){
      value <- Serreg[[a]][b]
      if(value > initialcodon && value < endcodon){
        AAs[a,(value%/%4 - (del-1))] <- "S"
      }
    }
  }
Tyrreg <- gregexpr("\\sTA[TC]",codons,useBytes = FALSE)
  ltyr <- length(Tyrreg)
  for(a in c(1:ltyr)){
    ltyrr <- length(Tyrreg[[a]])
    for(b in c(1:ltyrr)){
      value <- Tyrreg[[a]][b]
      if(value > initialcodon && value < endcodon){
        AAs[a,(value%/%4 - (del-1))] <- "Y"
      }
    }
  }
TAGgreg <- gregexpr("\\sTAG",codons,useBytes = FALSE)
  ltag <- length(TAGgreg)
  for(a in c(1:ltag)){
    ltagg <- length(TAGgreg[[a]])
    for(b in c(1:ltagg)){
      value <- TAGgreg[[a]][b]
      if(value > initialcodon && value < endcodon){
        AAs[a,(value%/%4 - (del-1))] <- "TAG"
      }
    }
  }
TAAgreg <- gregexpr("\\sTAA",codons,useBytes = FALSE)
  ltaa <- length(TAAgreg)
  for(a in c(1:ltaa)){
    ltaaa <- length(TAAgreg[[a]])
    for(b in c(1:ltaaa)){
      value <- TAAgreg[[a]][b]
      if(value > initialcodon && value < endcodon){
```

```
        AAs[a,(value%/%4 - (del-1))] <- NA
      }
    }
  }
Cysreg <- gregexpr("\\sTG[TC]",codons,useBytes = FALSE)
  lcys <- length(Cysreg)
  for(a in c(1:lcys)){
    lcyss <- length(Cysreg[[a]])
    for(b in c(1:lcyss)){
      value <- Cysreg[[a]][b]
      if(value > initialcodon && value < endcodon){
        AAs[a,(value%/%4 - (del-1))] <- "C"
      }
    }
  }
TGAgreg <- gregexpr("\\sTGA",codons,useBytes = FALSE)
  ltga <- length(TGAgreg)
  for(a in c(1:ltga)){
    ltgaa <- length(TGAgreg[[a]])
    for(b in c(1:ltgaa)){
      value <- TGAgreg[[a]][b]
      if(value > initialcodon && value < endcodon){
        AAs[a,(value%/%4 - (del-1))] <- NA
      }
    }
  }
Trpgreg <- gregexpr("\\sTGG",codons,useBytes = FALSE)
  ltrp <- length(Trpgreg)
  for(a in c(1:ltrp)){
    ltrpp <- length(Trpgreg[[a]])
    for(b in c(1:ltrpp)){
      value <- Trpgreg[[a]][b]
      if(value > initialcodon && value < endcodon){
        AAs[a,(value%/%4 - (del-1))] <- "W"
      }
    }
  }
Progreg <- gregexpr("\\sCC[GCAT]",codons,useBytes = FALSE)
  lpro <- length(Progreg)
  for(a in c(1:lpro)){
    lproo <- length(Progreg[[a]])
    for(b in c(1:lproo)){
      value <- Progreg[[a]][b]
      if(value > initialcodon && value < endcodon){
        AAs[a,(value%/%4 - (del-1))] <- "P"
      }
    }
  }
Hisgreg <- gregexpr("\\sCA[CT]",codons,useBytes = FALSE)
  lhis <- length(Hisgreg)
  for(a in c(1:lhis)){
    lhiss <- length(Hisgreg[[a]])
    for(b in c(1:lhiss)){
      value <- Hisgreg[[a]][b]
      if(value > initialcodon && value < endcodon){
        AAs[a,(value%/%4 - (del-1))] <- "H"
      }
    }
  }
Glngreg <- gregexpr("\\sCA[AG]",codons,useBytes = FALSE)
  lgln <- length(Glngreg)
  for(a in c(1:lgln)){
    lglnn <- length(Glngreg[[a]])
    for(b in c(1:lglnn)){
      value <- Glngreg[[a]][b]
```

```
      if(value > initialcodon && value < endcodon){
        AAs[a,(value%/%4 - (del-1))] <- "Q"
      }
    }
  }
Arggreg <- gregexpr("(\\sCG[GCAT])|(\\sAG[GA])",codons,useBytes = FALSE)
  larg <- length(Arggreg)
  for(a in c(1:larg)){
    largg <- length(Arggreg[[a]])
    for(b in c(1:largg)){
      value <- Arggreg[[a]][b]
      if(value > initialcodon && value < endcodon){
        AAs[a,(value%/%4 - (del-1))] <- "R"
      }
    }
  }
Ilegreg <- gregexpr("\\sAT[CAT]",codons,useBytes = FALSE)
  lile <- length(Ilegreg)
  for(a in c(1:lile)){
    lilee <- length(Ilegreg[[a]])
    for(b in c(1:lilee)){
      value <- Ilegreg[[a]][b]
      if(value > initialcodon && value < endcodon){
        AAs[a,(value%/%4 - (del-1))] <- "I"
      }
    }
  }
Metgreg <- gregexpr("\\sATG",codons,useBytes = FALSE)
  lmet <- length(Metgreg)
  for(a in c(1:lmet)){
    lmett <- length(Metgreg[[a]])
    for(b in c(1:lmett)){
      value <- Metgreg[[a]][b]
      if(value > initialcodon && value < endcodon){
        AAs[a,(value%/%4 - (del-1))] <- "M"
      }
    }
  }
Thrgreg <- gregexpr("\\sAC[GCAT]",codons,useBytes = FALSE)
  lthr <- length(Thrgreg)
  for(a in c(1:lthr)){
    lthrr <- length(Thrgreg[[a]])
    for(b in c(1:lthrr)){
      value <- Thrgreg[[a]][b]
      if(value > initialcodon && value < endcodon){
        AAs[a,(value%/%4 - (del-1))] <- "T"
      }
    }
  }
Asngreg <- gregexpr("\\sAA[CT]",codons,useBytes = FALSE)
  lasn <- length(Asngreg)
  for(a in c(1:lasn)){
    lasnn <- length(Asngreg[[a]])
    for(b in c(1:lasnn)){
      value <- Asngreg[[a]][b]
      if(value > initialcodon && value < endcodon){
        AAs[a,(value%/%4 - (del-1))] <- "N"
      }
    }
  }
Lysgreg <- gregexpr("\\sAA[AG]",codons,useBytes = FALSE)
  llys <- length(Lysgreg)
  for(a in c(1:llys)){
    llyss <- length(Lysgreg[[a]])
    for(b in c(1:llyss)){
```

```
      value <- Lysgreg[[a]][b]
      if(value > initialcodon && value < endcodon){
        AAs[a,(value%/%4 - (del-1))] <- "K"
      }
    }
  }
Valgreg <- gregexpr("\\sGT[GACT]",codons,useBytes = FALSE)
  lval <- length(Valgreg)
  for(a in c(1:lval)){
    lvall <- length(Valgreg[[a]])
    for(b in c(1:lvall)){
      value <- Valgreg[[a]][b]
      if(value > initialcodon && value < endcodon){
        AAs[a,(value%/%4 - (del-1))] <- "V"
      }
    }
  }
Alagreg <- gregexpr("\\sGC[GACT]",codons,useBytes = FALSE)
  lala <- length(Alagreg)
  for(a in c(1:lala)){
    lalaa <- length(Alagreg[[a]])
    for(b in c(1:lalaa)){
      value <- Alagreg[[a]][b]
      if(value > initialcodon && value < endcodon){
        AAs[a,(value%/%4 - (del-1))] <- "A"
      }
    }
  }
Aspgreg <- gregexpr("\\sGA[TC]",codons,useBytes = FALSE)
  lasp <- length(Aspgreg)
  for(a in c(1:lasp)){
    laspp <- length(Aspgreg[[a]])
    for(b in c(1:laspp)){
      value <- Aspgreg[[a]][b]
      if(value > initialcodon && value < endcodon){
        AAs[a,(value%/%4 - (del-1))] <- "D"
      }
    }
  }
Glugreg <- gregexpr("\\sGA[AG]",codons,useBytes = FALSE)
  lglu <- length(Glugreg)
  for(a in c(1:lglu)){
    lgluu <- length(Glugreg[[a]])
    for(b in c(1:lgluu)){
      value <- Glugreg[[a]][b]
      if(value > initialcodon && value < endcodon){
        AAs[a,(value%/%4 - (del-1))] <- "E"
      }
    }
  }
Glygreg <- gregexpr("\\sGG[GACT]",codons,useBytes = FALSE)
  lgly <- length(Glygreg)
  for(a in c(1:lgly)){
    lglyy <- length(Glygreg[[a]])
    for(b in c(1:lglyy)){
      value <- Glygreg[[a]][b]
      if(value > initialcodon && value < endcodon){
        AAs[a,(value%/%4 - (del-1))] <- "G"
      }
    }
  }
AAs <- as.data.frame(AAs)
countAAs <- apply(AAs, 2, table)
```

```
#this counts sequences that have TAG codons, sequences that have more than one are only
counted once
TAGreg <- regexpr("\\sTAG",codons)
TAGtable <- table(TAGreg)
percentTAG <- sum(TAGtable[2:length(TAGtable)])/length(codons)*100#percent of sequences
containing TAG

#this creates heatmap for amino acid frequency per library position, change scale according
to values
AAtable <- apply(AAs, 2, table)
AAtable <- as.matrix(AAtable/length(codons))
colnames(AAtable) <- c(1:lib)
heatmapcolors <- colorRampPalette(brewer.pal(9,"Blues"))(100)
sc <- seq(0.0,0.3,by=0.003)
AAheatmap <- heatmap.2(AAtable, Rowv = NA, Colv = NA, col = heatmapcolors, density.info =
"none", scale = "none", trace = "none", breaks = sc,  xlab = "Position in Library", ylab =
"Codon", margins = c(3,4), dendrogram = "none")

#this creates projected heatmap based on NNK randomized codons
randomAAs <- matrix(0,21,lib,dimnames = list(rownames(AAtable),c(1:7)))
randomAAs[c("A","G","P","T","V"),] <- 2/32
randomAAs[c("C","H","Q","N","K","Y","D","E","W","I","M","TAG","F"),] <- 1/32
randomAAs[c("L","S","R"),] <- 3/32
NNKheatmap <- heatmap.2(randomAAs, Rowv = NA, Colv = NA, col = heatmapcolors, density.info
= "none", scale = "none", trace = "none", breaks = sc,  xlab = "Position in Library", ylab
= "Codon", margins = c(3,4), dendrogram = "none")

#this creates heatmap showing bias from random, change scale with respect to range of
values
lscale <- seq(-1,4,by=5/100)
librarybias <- (AAtable - randomAAs)/randomAAs
Biasheatmap <- heatmap.2(librarybias, Rowv = NA, Colv = NA, col = heatmapcolors,
density.info = "none", scale = "none", trace = "none", breaks = lscale,  xlab = "Position
in Library", ylab = "Codon", margins = c(3,4), dendrogram = "none")
```

Suppementary R Script 2

NNK Analysis.R

```
#NNK Analysis of Sequences in library,ignoring TAG codons, do this after defining TAG
positions in AAs
#Make sure variables are defined from the AA analysis
l <- length(codons)
matrixseq <- matrix(0,l,3*lib)
Ggreg <- gregexpr("G",codons,useBytes = FALSE)
  lg <- length(Ggreg)
  for(a in c(1:lg)){
    lgg <- length(Ggreg[[a]])
    for(b in c(1:lgg)){
      value <- Ggreg[[a]][b]
      if(value > initialcodon && value < endcodon+1){
        if(AAs[a,(value%/%4 - (del-1))] != "TAG" | is.na(AAs[a,(value%/%4 - (del-1))])){
          matrixseq[a,(value-del*3-(value%/%4))] <- "G"
        }
      }
    }
  }
Cgreg <- gregexpr("C",codons,useBytes = FALSE)
  lc <- length(Cgreg)
  for(a in c(1:lc)){
    lcc <- length(Cgreg[[a]])
    for(b in c(1:lcc)){
      value <- Cgreg[[a]][b]
      if(value > initialcodon && value < endcodon+1){
        if(AAs[a,(value%/%4 - (del-1))] != "TAG" | is.na(AAs[a,(value%/%4 - (del-1))])){
          matrixseq[a,(value-del*3-(value%/%4))] <- "C"
        }
      }
    }
  }
Agreg <- gregexpr("A",codons,useBytes = FALSE)
  la <- length(Agreg)
  for(a in c(1:la)){
    laa <- length(Agreg[[a]])
    for(b in c(1:laa)){
      value <- Agreg[[a]][b]
      if(value > initialcodon && value < endcodon+1){
        if(AAs[a,(value%/%4 - (del-1))] != "TAG" | is.na(AAs[a,(value%/%4 - (del-1))])){
          matrixseq[a,(value-del*3-(value%/%4))] <- "A"
        }
      }
    }
  }
Tgreg <- gregexpr("T",codons,useBytes = FALSE)
  lt <- length(Tgreg)
  for(a in c(1:lt)){
    ltt <- length(Tgreg[[a]])
    for(b in c(1:ltt)){
      value <- Tgreg[[a]][b]
      if(value > initialcodon && value < endcodon+1){
        if(AAs[a,(value%/%4 - (del-1))] != "TAG" | is.na(AAs[a,(value%/%4 - (del-1))])){
          matrixseq[a,(value-del*3-(value%/%4))] <- "T"
        }
      }
    }
  }
matrixseq <- as.data.frame(matrixseq)

#counts nucleotides for all of the positions in matrixseq, returns a table with amount of
nucleotides at each position
count <- apply(matrixseq, 2, table)
```

```
View(count)
```

Suppementary R Script 3

Filter Reads with Dada Platform.R

```
library(dada2)
#create path to unzipped NGS fastq files
path <- "/Users/traehampton/Desktop/NGS FastQ"
NNK7F <- "/Users/traehampton/Desktop/NGS FastQ/1_S1_L001_R1_001.fastq"
NNK7R <- "/Users/traehampton/Desktop/NGS FastQ/1_S1_L001_R2_001.fastq"
plotQualityProfile(NNK7F)
plotQualityProfile(NNK7R)
#creates directory for filtered files
filtFs <- file.path(path, "filtered", paste0(NNK7F, "_F2_filt.fastq"))
filtRs <- file.path(path, "filtered", paste0(NNK7R, "_R2_filt.fastq"))

#this filters the reads, set trim length corresponding to poor sequence quality from plots
out <- filterAndTrim(NNK7F, filtFs, NNK7R, filtRs, truncLen=c(120,95),
                     maxN=1, maxEE=c(1,1), rm.phix=FALSE,
                     compress=FALSE, multithread=TRUE) # On Windows set multithread=FALSE
head(out)
```

SUPPLEMENTARY REFERENCES

1. Lee, Y. J., Wu, B., Raymond, J. E., Zeng, Y., Fang, X. Q., Wooley, K. L., and Liu, W. R. A Genetically Encoded Acrylamide Functionality, *Acs Chem Biol* **8**, 1664-1670 (2013).
