# Supplementary material for: An amber obligate active site-directed ligand evolution technique for phage display
Source: Nat Commun. 2020 Mar 13;11:1392. doi: 10.1038/s41467-020-15057-7 (PMC7070036; doi:10.1038/s41467-020-15057-7)
Supplement: Supplementary file 7 — Reporting Summary [file 41467_2020_15057_MOESM7_ESM.pdf]

nature research

Corresponding author(s): Wenshe R. Liu and Shiqing Xu

Last updated by author(s): Jan 31, 2020

Reporting Summary

Nature Research wishes to improve the reproducibility of the work that we publish. This form provides structure for consistency and transparency in reporting. For further information on Nature Research policies, see Authors & Referees and the Editorial Policy Checklist.

Statistics

For all statistical analyses, confirm that the following items are present in the figure legend, table legend, main text, or Methods section.

n/a	Confirmed	
☐	☒	The exact sample size (n) for each experimental group/condition, given as a discrete number and unit of measurement
☐	☒	A statement on whether measurements were taken from distinct samples or whether the same sample was measured repeatedly
☒	☐	The statistical test(s) used AND whether they are one- or two-sided<br>*Only common tests should be described solely by name; describe more complex techniques in the Methods section.*
☐	☒	A description of all covariates tested
☐	☒	A description of any assumptions or corrections, such as tests of normality and adjustment for multiple comparisons
☐	☒	A full description of the statistical parameters including central tendency (e.g. means) or other basic estimates (e.g. regression coefficient) AND variation (e.g. standard deviation) or associated estimates of uncertainty (e.g. confidence intervals)
☒	☐	For null hypothesis testing, the test statistic (e.g. F, t, r) with confidence intervals, effect sizes, degrees of freedom and P value noted<br>*Give P values as exact values whenever suitable.*
☒	☐	For Bayesian analysis, information on the choice of priors and Markov chain Monte Carlo settings
☒	☐	For hierarchical and complex designs, identification of the appropriate level for tests and full reporting of outcomes
☒	☐	Estimates of effect sizes (e.g. Cohen's d, Pearson's r), indicating how they were calculated

Our web collection on statistics for biologists contains articles on many of the points above.

Software and code

Policy information about availability of computer code

Data collection	Schrodinger (Release 2017-4) and the Desmond module within Schrodinger were used to perform molecular dynamics simulations of the Sirtuin 2 Inhibitors.
Data analysis	GraphPad Prism (8.1.2) was used for fitting inhibition and binding curves of all peptides. For analysis of Illumina sequencing, R scripts were written. These are included in the supplementary information and have also been uploaded to github under the repository jhampton1/NNK7-Amber-Analysis.

For manuscripts utilizing custom algorithms or software that are central to the research but not yet described in published literature, software must be made available to editors/reviewers. We strongly encourage code deposition in a community repository (e.g. GitHub). See the Nature Research guidelines for submitting code & software for further information.

Data

Policy information about availability of data

All manuscripts must include a data availability statement. This statement should provide the following information, where applicable:

- Accession codes, unique identifiers, or web links for publicly available datasets
- A list of figures that have associated raw data
- A description of any restrictions on data availability

The data that support the findings of this study are available from the corresponding author upon reasonable request. Novel plasmids are available through Addgene with the following identification numbers shown in parentheses: pEVOL-pylT-N346A/C348A (127411), M13K07-g3TAA (127414), pEDF-PhdRS (127445). Given the large size of the Illumina sequencing results and the molecular dynamics simulations, these data are available upon reasonable request from the corresponding author. The source data underlying Fig. 4, 5a-c, and 6 and Supplementary Fig. 28 and 29 are provided as a Source Data file.

Field-specific reporting

Please select the one below that is the best fit for your research. If you are not sure, read the appropriate sections before making your selection.

☒ Life sciences ☐ Behavioural & social sciences ☐ Ecological, evolutionary & environmental sciences

For a reference copy of the document with all sections, see nature.com/documents/nr-reporting-summary-flat.pdf

Life sciences study design

All studies must disclose on these points even when the disclosure is negative.

Sample size	No sample size calculations were performed. N values are given for all experiments in the main text and supplementary information. Sample sizes were determined to ensure reproducibility of all experiments. All experiments were repeated to give confidence in the obtained results. As all sample sizes afforded reproducible results, we deemed these to be sufficient in making conclusions.
Data exclusions	No data was excluded from the experiments reported in the draft.
Replication	All experiments were replicated at least twice (n values for each experiment is given in the main text and supplementary information) to ensure the data obtained was reproducible. All attempts at replication were successful.
Randomization	This study was done against a targeted recombinantly expressed protein. Therefore, randomization of elements was not relevant to experimental design.
Blinding	Blinding is not relevant in our study, as there was no grouping of organisms/samples that may cause bias in measurements.

Reporting for specific materials, systems and methods

We require information from authors about some types of materials, experimental systems and methods used in many studies. Here, indicate whether each material, system or method listed is relevant to your study. If you are not sure if a list item applies to your research, read the appropriate section before selecting a response.

Materials & experimental systems

n/a	Involved in the study	
☒	☐	Antibodies
☒	☐	Eukaryotic cell lines
☒	☐	Palaeontology
☒	☐	Animals and other organisms
☒	☐	Human research participants
☒	☐	Clinical data

Methods

n/a	Involved in the study	
☒	☐	ChIP-seq
☒	☐	Flow cytometry
☒	☐	MRI-based neuroimaging
