# Supplementary material for: An amber obligate active site-directed ligand evolution technique for phage display
Source: Nat Commun. 2020 Mar 13;11:1392. doi: 10.1038/s41467-020-15057-7 (PMC7070036; doi:10.1038/s41467-020-15057-7)
Supplement: Supplementary file 8 — Description of Additional Supplementary Files [file 41467_2020_15057_MOESM8_ESM.pdf]

Title: Supplementary Video 1
Description: Molecular Simulations for Interactions of S2P03 with SIRT2. This video is composed of a selection of frames from the simulations, highlighting hydrophobic interactions between the valine and threonine adjacent to tBuK and F235, L239, and F244 of SIRT2.

Title: Supplementary Video 2
Description: Molecular Simulations for Interactions of S2P04 with SIRT2. This video is composed of a selection of frames from the simulations, highlighting hydrophobic interactions between the valine and isoleucine adjacent to tBuK and F235 and L239 of SIRT2.

Title: Supplementary Video 3
Description: Molecular Simulations for Interactions of S2P07 with SIRT2. This video is composed of a selection of frames from the simulations, highlighting hydrophobic interactions between the phenylalanine and valine adjacent to tBuK and L239 and F235 of SIRT2, respectively.

Title: Supplementary Video 4
Description: Molecular Simulations for Interactions of S2P04-5 with SIRT2. This video is composed of a selection of frames from the simulations, highlighting hydrophobic interactions between the valine and isoleucine adjacent to tBuK and L239 and F235 of SIRT2, respectively. The truncation of the S2P04 peptide results in less interactions with the two residues of SIRT2 (See Supplementary Fig. 32).

Title: Supplementary Video 5
Description: Molecular Simulations for Interactions of Ac-S2P04-5 with SIRT2. This video is composed of a selection of frames from the simulations, highlighting hydrophobic interactions between the valine and isoleucine adjacent to tBuK and L239 and F235 of SIRT2, respectively. The acetylated N-terminus results less interactions with L239 compared to the S2P04 (See Supplementary Fig. 32).
